# Structurally Uncommon Secondary Metabolites Derived from Endophytic Fungi

**DOI:** 10.3390/jof7070570

**Published:** 2021-07-17

**Authors:** Humberto E. Ortega, Daniel Torres-Mendoza, Zuleima Caballero E., Luis Cubilla-Rios

**Affiliations:** 1 Laboratory of Tropical Bioorganic Chemistry, Faculty of Natural, Exact Sciences and Technology, University of Panama, Panama City 0824, Panama; humberto.ortegad@up.ac.pa (H.E.O.); daniel-t.torres-m@up.ac.pa (D.T.-M.); 2 Department of Organic Chemistry, Faculty of Natural, Exact Sciences and Technology, University of Panama, Panama City 0824, Panama; 3 Vicerrectoría de Investigación y Postgrado, Universidad de Panamá, Panama City 0824, Panama; 4 Center of Cellular and Molecular Biology of Diseases, Institute for Scientific Research and Technology Services (INDICASAT-AIP), Clayton 0843-01103, Panama; zcaballero@indicasat.org.pa

**Keywords:** endophytic fungi, uncommon secondary metabolites, biological activity

## Abstract

Among microorganisms, endophytic fungi are the least studied, but they have attracted attention due to their high biological diversity and ability to produce novel and bioactive secondary metabolites to protect their host plant against biotic and abiotic stress. These compounds belong to different structural classes, such as alkaloids, peptides, terpenoids, polyketides, and steroids, which could present significant biological activities that are useful for pharmacological or medical applications. Recent reviews on endophytic fungi have mainly focused on the production of novel bioactive compounds. Here, we focus on compounds produced by endophytic fungi, reported with uncommon bioactive structures, establishing the neighbor net and diversity of endophytic fungi. The review includes compounds published from January 2015 to December 2020 that were catalogued as unprecedented, rare, uncommon, or possessing novel structural skeletons from more than 39 different genera, with *Aspergillus* and *Penicillium* being the most mentioned. They were reported as displaying cytotoxic, antitumor, antimicrobial, antiviral, or anti-inflammatory activity. The solid culture, using rice as a carbon source, was the most common medium utilized in the fermentation process when this type of compound was isolated.

## 1. Introduction

Endophytic fungi colonize the internal tissue of plants without causing harm or disease [1]. They can offer protection against predators, pathogens, and abiotic stresses to their host plant [2,3,4,5]. In the past decade, the number of patents has increased to apply endophytic fungi for agricultural, bio-, and phytoremediation purposes, and for the production of active natural products with biomedical applications [6,7].

Endophytic fungi are an important source of active natural products with great chemical diversity which is largely untapped. This biosynthetic capacity and induction for producing secondary metabolites could be related to the activation of genes [8,9]. Many of these compounds possess novel skeletons with antibacterial, antifungal, antiviral, anti-inflammatory, antitumor, antimalarial, and other activities, and belong to different classes, such as alkaloids, terpenoids, flavonoids, phenolic compounds, and steroids [10].

Some endophytic fungi can produce the same secondary metabolites of their host plants that are medically important drugs, such as taxol^®^, increasing the likelihood of using such endophytes as an alternative and sustainable source for producing these compounds faster than in plants [10].

The production of secondary metabolites by endophytes could be impacted by biotic and abiotic factors, but under lab conditions, due to the selective variation of parameters during cultivation, the culture media and the induction of stress through competition with microorganisms in cocultures represent interesting ways to generate biological activity, chemical diversity, and/or novel uncommon molecules [11,12].

Among the endophytic fungi described in the literature, the genus *Aspergillus* is the most dominant, representing a rich source of diversity of bioactive natural products [13]. In this review, we cover secondary metabolites from endophytic fungus with uncommon skeletons published from 2015 to 2020. Genera with three or more references comprised the species of *Aspergillus*, *Penicillium*, *Trichoderma*, *Chaetomium*, *Xylaria*, *Phomopsis*, *Pestalotiopsis,* and *Talaromyces*. Most of the selected articles showed species characterized through morphological and genetic aspects, mostly using the ITS region and to a lesser extent the 18S region of the ribosomal gene. We use this information to highlight the genetic diversity of endophytes and their ability to produce secondary metabolites with uncommon chemical structures.

## 2. Materials and Methods

The search was initially conducted in Scifinder^®^ using the term “endophytic fungi”, and 5942 references were found. Removing duplicates, and refining papers by year (2015–2020), by document type (journal, letter, and review), and by research topic “natural products” resulted in 654 references. Then, we selected 90 articles based on the rareness, uncommonness, unprecedentedly, or novelty on the structure or skeleton of the compounds.

The homologous sequences of the internal transcribed spacer (ITS1) of the ribosomal gene of different species of endophytic fungi were retrieved from the sequence banks of the National Center for Biotechnology Information (NCBI) through the BLAST^®^ tool (https://blast.ncbi.nlm.nih.gov/Blast.cgi, accessed on 14 July 2021). The search for sequences in the NCBI databases followed the rules recommended by expert mycologists to work with ITS data [14]. The sequences were aligned using the GeneDoc v2.7 program and subsequently analyzed using the neighbor-net phylogenetic-network method with the Kimura 2-parameter model applied in the Splits Tree4 v4.16.2 as previously described (http://nrbsc.org/gfx/genedoc/, accessed on 14 July 2021). Support values were estimated by applying 100 bootstrap replicates. A total of 67 sequences were analyzed, where 32 corresponded to the fungal species described in this review, and 35 were included to represent species of which the genera did not have available sequences in the sequence bank (Table S1). Analysis was also reinforced with a greater representation of taxa.

## 3. Results

Since the production of secondary metabolites is dependent on culture settings, around 55% of the articles analyzed here showed the use of rice soaked in distilled water as the main culture medium for fermentation, but it was seeded with a plethora of short-period liquid-culture conditions. Modifications to the solid medium included the addition of natural seawater, saline water, and peptone. Fermentation on a solid rice medium was for long periods (45 days, average) in static conditions in dark or 12/12 h light/dark periods at room temperature. The PDA/PDB medium was the second most used (20%) for fermentation. The rest of the described media were malt extract (MEA), Czapek, and Peptone–Yeast–Glucose (PYG), among others. As opposed to solid fermentations, liquid ones were carried out in rotary shakers at 120 rpm at short periods (15 days, average), controlled pH, and varied conditions of complete dark, light, or 12/12 h dark/light cycles at room temperature.

In total, 202 novel natural products were selected based on their structure skeleton. They were classified as alkaloids (cytochalasans, indol alkaloids, isoindole, pyrrolidone, pyridone, pyridinyl, diketopiperazines derivatives, and other nitrogen-containing compounds), peptides, terpenoids (including sesquiterpenoids, diterpenoids, sesterterpenoids, and meroterpenoids), polyketides, and steroids.

### 3.1. Alkaloids

#### 3.1.1. Cytochalasans

Cytochalasans comprise a structurally diverse polyketide–nonribosomal peptide group of secondary metabolites that feature a substituted isoindole scaffold fused with a macrocyclic ring and show a broad range of biological activities [15]. 

**Figure 1:**
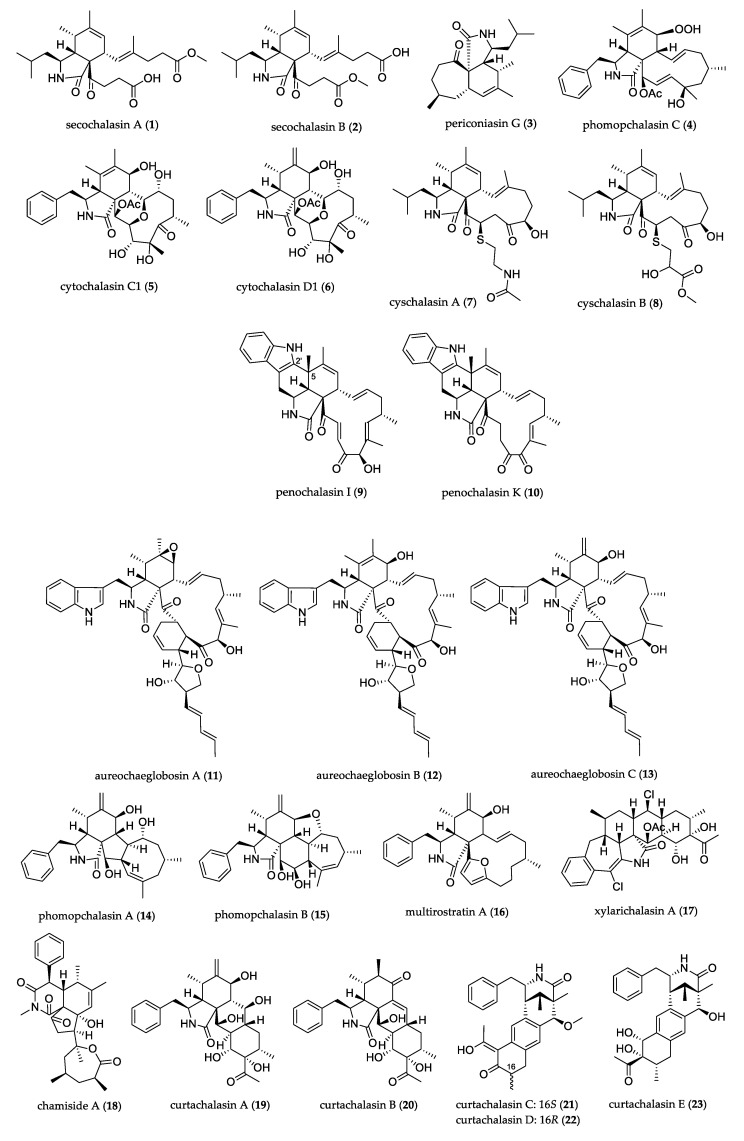
Structure of cytochalasans.

Normally, the cytochalasan structure includes tricyclic or tetracyclic ring systems and contains a 9–15-membered macrocyclic system. Figure 1 shows the structures of uncommon cytochalasans; secochalasins A (**1**) and B (**2**) isolated from *Aspergillus micronesiensis* represent a new undescribed skeleton of bicyclic 17, 18-*seco*-aspochalasins [16]. Periconiasin G (**3**) possesses a unique 7/6/5 tricyclic ring system, in which the seven-membered ring is the smallest carbocyclic ring in typical tricyclic cytochalasans. It was isolated from *Periconia* sp. and showed weak anti-HIV activity [17].

Among 11-member macrocyclic system compounds, phomopchalasin C (**4**) isolated from *Phomopsis* sp. possesses a distinguished rare peroxide functionality and shows moderate cytotoxicity against H-60, SMMC-7721, and A-549 cell lines, and inhibitory activity against NO production in LPS-activated RAW 264.7 macrophages [18]. Cytochalasins C1 (**5**) and D1 (**6**), isolated from *Xylaria* cf. *curta*, featured a unique macrocycle with an oxygen bridge. These two compounds showed moderate cytotoxicity against the HL-60 cell line [19]. Cyschalasins A (**7**) and B (**8**) were formed by the fusion of an aspochalasin with a modified cysteine residue, connected to C-20 of the aspochalasin via a sulfur atom. Cytochalasans containing sulfur are rare in nature. The two compounds were isolated along with **1** and **2**. Compounds **7** and **8** showed antimicrobial activity against Gram-positive bacteria and fungi (*Staphylococcus aureus*, MRSA, and *Candida albicans*), and moderate cytotoxicity against cancer cells (HL-60, A-549, Hep3B, MCF-7, and SW-480), while **1** and **2** were inactive [16].

Among the 13-member macrocyclic system compounds, penochalasin I (**9**) and penochalasin K (**10**) possess an unprecedented six-cyclic 6/5/6/5/6/13 fused ring; they are the first example of a system formed by the connection of C-5 and C-2’ of the chaetoglobosin class. These compounds were isolated from *Penicillium chrysogenum* and showed cytotoxicity against MDA-MB-435 and SGC-7901 cells, and inhibitory activity against *Rhizoctonia solani* and *Colletotrichum gloeosporioides* [20,21]. Aureochaeglobosins A–C (**11**–**13**) were isolated from *Chaetomium globosum*. They represent the first adduct examples of chaetoglobosins where, in an aureonitol derivative fused via [4 + 2], cycloaddition is present. Compounds **12**–**13** showed significant cytotoxicity against MDA-MB-231 cancer cells [22]. Phomopchalasin A (**14**), a 5/6/5/8 fused tetracyclic ring system, and phomopchalasin B (**15**), a 5/6/6/7/5 joined pentacyclic skeleton, were isolated from *Phomopsis* sp. Compound **15** exhibited an antimigratory effect against MDA-MB-231 in vitro [18]. Multirostratin A (**16**) is the first cytochalasin with a macrocyclic ring including a furan moiety, isolated from *Phoma multirostrata*. It showed cytotoxicity against the HL-60, A-54, SMMC-7721, MCF-7, and SW-480 tumor cell lines [23].

An atypical cytochalasan compound, xylarichalasin A (**17**) possesses a 6/7/5/6/6/6 bonded polycyclic structure with two chlorine substitutions representing a new class of cytochalasan with a unique benzo[7]annulene/pyrrolidine/perhydroanthracene fused core. Compound **17,** isolated from *Xylaria* cf. *curta*, showed cytotoxicity against SMMC-7721 and MCF-7 cell lines better than that of the drug cisplatin [24]. The novel chamiside A (**18**) presented a novel 6/6/5 fused tricyclic core skeleton bearing a benzene ring and a rare seven-member lactone; it was isolated from *Chaetomium nigricolor* and exhibited antibacterial activity against *S. aureus* [25]. Five compounds (**19**–**23**) were isolated from endophyte *Xylaria* cf. *curta***.** Curtachalasins A (**19**) and B (**20**) were the first reported tetracyclic 10-pheylcytochalasans with a pyrrolidine/perhydroanthracene (5/6/6/6) fused core, and they showed weak antifungal activity against *Microsporum gypseum* [26]. Curtachalasins C-E (**21**–**23**) possesses an unprecedented bridge lactam 6/6/6/6 ring system. Compound **21** showed significant resistance reversal activity against fluconazole-resistant *C. albicans* [27].

#### 3.1.2. Indole Alkaloids

**Figure 2:**
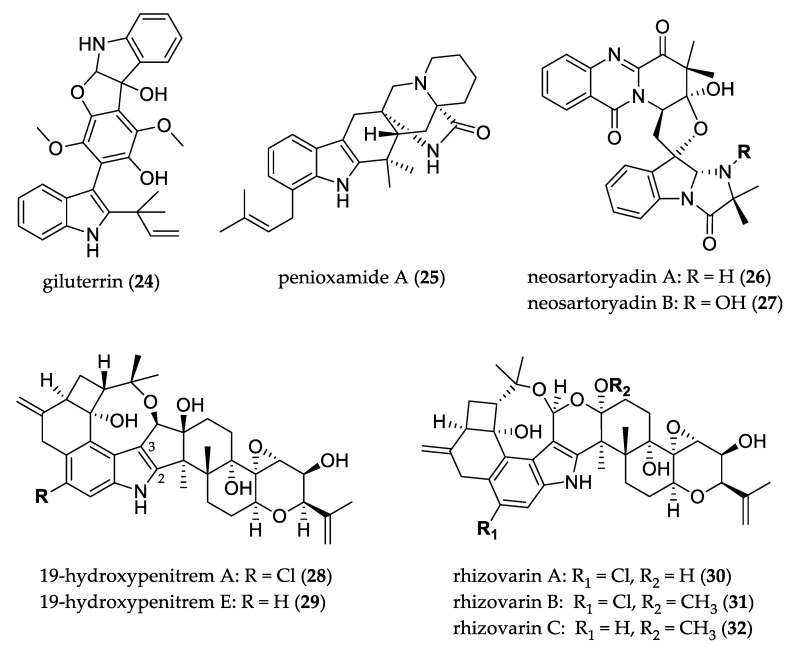
Structure of indole alkaloids.

Indole alkaloids constitute a group of nitrogen-containing secondary metabolites with interesting chemical structures and diverse biological activities [28]. Figure 2 shows the structures of uncommon indole alkaloids. Giluterrin (**24**), a prenylated indole alkaloid that presented an unprecedented carbon skeleton, was isolated from endophytic fungus *Aspergillus terreus* and presented an antiproliferative profile for prostate (PC-3) and kidney (786-0) cancer cell lines [29]. Another rare prenylated indole alkaloid is penioxamide A (**25**), possessing a piperidine moiety and bearing an antirelative configuration in the bicyclo[2.2.2]diazaoctane ring, and was isolated from *Penicillium oxalicum* and showed potent brine-shrimp lethality with LD_50_ value of 5.6 μM [30].

Neosartoryadin A (**26**) and B (**27**), quinazoline-containing indole alkaloids with a 6/6/6/5 quinazoline ring system connected directly to a 6/6/6 imidazoindolone ring system, were isolated from mangrove-derived fungus *Neosartorya udagawae*. Both compounds displayed anti-influenza virus A (H1N1) activity exhibiting inhibitory effects with values of 66 and 58 μM, respectively [31].

Penitrems are rare indole alkaloids that feature a C19 skeleton fused to positions C-2 and C-3 of the indole ring; 19-hydroxypenitrem A (**28**) and its dechlorinated derivative 19-hydroxypenitrem E (**29**) were isolated from algal *Aspergillus nidulans* and showed cytotoxic activity against brine shrimp, and antimicrobial activity against both human (*E. coli*, *S. aureus*) and aqua pathogens (*Edwardsiella tarda*, *Vibrio anguillarum*) [32]. An investigation on mangrove-derived fungus *Mucor irregularis* led to the isolation of indole diterpenes; rhizovarins A–C (**30**–**32**) represent complex members featuring an unusual acetal linked to a hemiketal or ketal in an unprecedented 4/6/6/8/5/6/6/6/6-fused indole–diterpene ring system. These compounds possess an eight-membered cyclic ether system coupled with five other rings with cyclobutane, methylenecyclohexane, indole, and 3,6-dihydro-2*H*-pyran motifs. Compounds **30**–**31** showed activity against the human A-549 and HL-60 cancer cell lines, and **32** showed weak or no activity [33].

#### 3.1.3. Isoindole Derivatives

**Figure 3:**
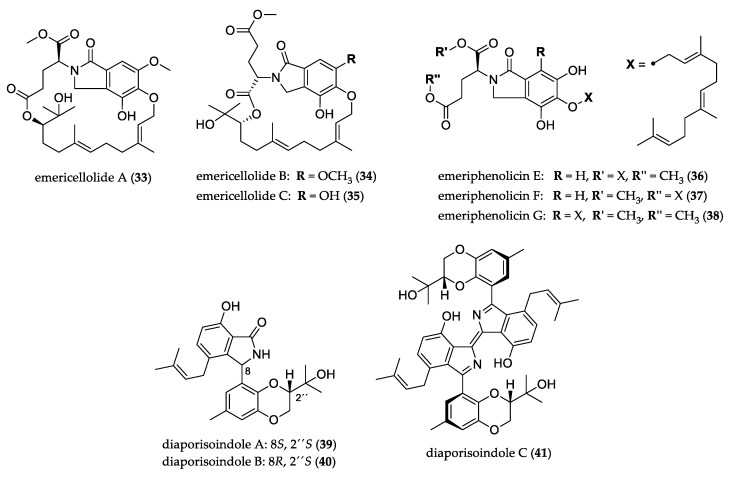
Structure of isoindole derivatives.

Isoindole, a fused benzopyrrole ring system, is the regioisomer of indole heterocycle. Its derivatives have attracted scientific attention for decades, and can be found in natural and pharmaceutical products such as indolocarbazoles, macrocyclic polyketides, alkaloids, and meroterpenoids [34,35,36]. Figure 3 shows the structures of uncommon isoindole derivatives. Emericellolides A–C (**33**–**35**) feature an unprecedented macrolide skeleton with an unusual L-glutamate fragment, isoindolone unit, and a sesquiterpene moiety, while emeriphenolicins E–G (**36**–**38**) showed two farnesyl groups attached to one isoindolone unit. Both classes were isolated from *Emericella nidulans*. Only compound **36** showed cytotoxicity against the HeLa, A-549, and HCT-116 human cancer cell lines [37].

Diaporisoindoles A–B (**39**–**40**), two novel isoprenylisoindoles with rare 1,4-benzodioxan moiety, and diaporisoindole C (**41**), having an unprecedented diisoprenylisoindole dimer skeleton, were isolated from *Diaporthe* sp. Compound **41** exhibited inhibitory activity against *Mycobacterium tuberculosis* protein tyrosine phosphatase B [38].

#### 3.1.4. Pyrrolidone Derivatives

**Figure 4:**
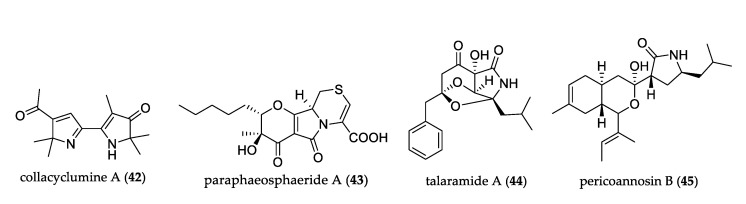
Structure of pyrrolidone derivatives.

Pyrrolidine alkaloids are widely distributed in nature and display some interesting biological activities, such antitumor and anti-inflammatory, but microbe-derived ones are rare. Figure 4 shows the structures of uncommon pyrrolidine alkaloids. Collacyclumine A (**42**) was the first case of a dimeric pyrrolidine alkaloid in nature, and it was isolated from mangrove-derived *Colletotrichum salsolae* but did not show cytotoxic activity [39]. Paraphaeosphaeride A (**43**), which possesses a 4-pyranone-γ-lactam-1,4-thiazine moiety, was isolated from endophytic fungus *Paraphaeosphaeria neglecta*. This compound might provide a new target for synthesis or biosynthetic investigation [40]. Talaramide A (**44**) is the second example of an alkaloid that possesses a unique oxidized tricyclic system. It was isolated from *Talaromyces* sp. and displayed promising inhibition of mycobacterial PknG activity [41]. Pericoannosin B (**45**) was isolated from *Periconia* sp. and featured the uncommon hexahydro-1*H*-isochromen-5-isobutylpyrrolidin-2-one skeleton [42].

#### 3.1.5. Pyridone and Pyridinyl Derivatives

Pyridone alkaloid asperpyridone A (**46**) possesses the unusual pyrano[3,2-*c*]pyridine scaffold and was isolated from *Aspergillus* sp. This compound demonstrated potential use to develop agents for diabetes treatment due to the pronounced glucose uptake effect on liver HepG2 cells under normal and insulin-resistant conditions [43]. Pair of epimers campyridones A–B (**47**–**48**) and C–D (**49**–**50**) were isolated from *Campylocarpon* sp. and featured spiro-furanone or γ-pyrone moieties, which represent a new family of 4-hydroxy-3-pyridone alkaloids. Only compound **50** showed cytotoxicity against HeLa cells [44].

**Figure 5:**
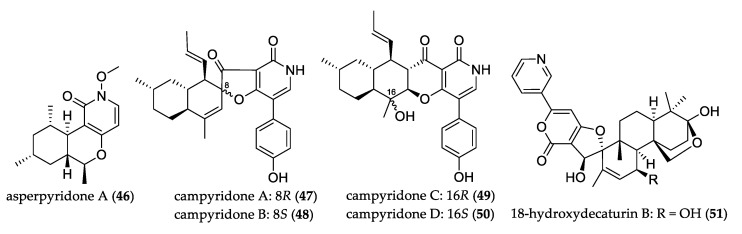
Structure of pyridone and pyridinyl derivatives.

The 18-hydroxydecaturin B (**51**) is a novel pyridinyl-α-pyrone that is rare among natural products, and it was isolated from *Penicillium oxalicum* and showed brine-shrimp lethality with LD_50_ value of 2.3 μM [30]. Figure 5 shows the structure of uncommon pyridone and pyridinyl derivatives.

#### 3.1.6. Diketopiperazine Derivatives

**Figure 6:**
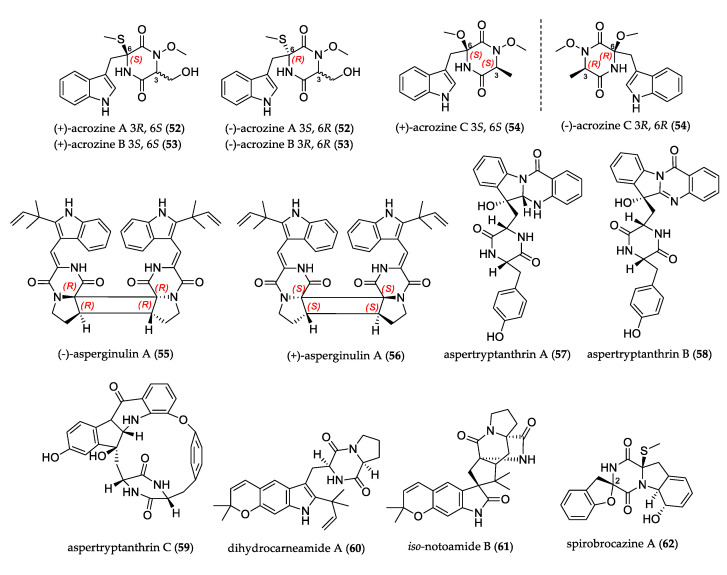

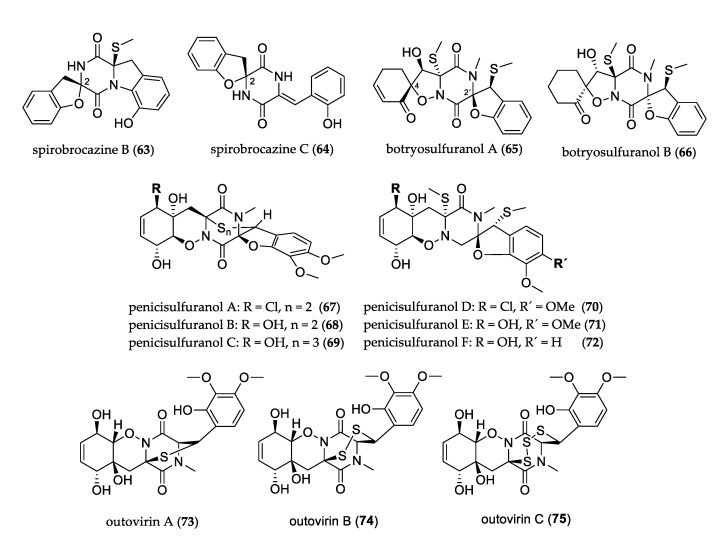
Structure of diketopiperazine derivatives.

2,5-Diketopiperazines are cyclopeptides formed by the condensation of two amino acids such as serine, tyrosine, tryptophan, proline, phenylalanine, leucine, isoleucine, histidine, or alanine. They are abundant in nature and are found alone or embedded in larger and complex structures from fungi, bacteria, plants, and mammals [45,46]. Figure 6 shows the structures of uncommon diketopiperazine derivatives.

Acrozines A–C (**52**–**54**) are three pairs of indole diketopiperazine enantiomers containing the uncommon N-methoxy moiety. They were isolated from the culture of the green alga-endophytic *Acrostalagmus luteoalbus*. Pair compounds (+)–**53**/(–)–**53** showed antifungal activity, and (+)–**52** displayed better inhibitory activity against acetylcholinesterase than (–)–**52** did [47]. Enantiomers (+)– and (–)–asperginulin A (**55**–**56**) were isolated from mangrove-derived *Aspergillus* sp. These indole diketopiperazines featured an unprecedented 6/5/4/5/6 pentacyclic skeleton. Compound **55** showed antifouling activity against *Balanus reticulatus* [48]. Aspertryptanthrins A–C (**57**–**59**), isolated from *Aspergillus* sp., have a tryptanthrin skeleton formed by a tryptophan moiety and anthranilate unit; compound **59** also featured a rare 16-membered ring. They were evaluated against the U-20S, MCF-7, HepG2, and HeLa cell lines, but did not show activity [49]. Prenylated indole diketopiperazines, dihydrocarneamide A (**60**), and *iso*-notoamide B (**61**) were isolated from *Paecilomyces variotii* and represented rare examples of C-5 prenylation, forming a fused dimethyldihydropyran ring at C-5 and C-6 of the indole ring. Both compounds displayed weak activity against the NCI-H460 cell line [50].

Spirobrocazines A–C (**62**–**64**) with a rare C-2 spirocyclic skeleton were isolated from *Penicillium brocae* and presented a 6/5/6/5/6 cyclic system. Compound **62** exhibited moderate antibacterial activities against *E. coli*, *S. aureus,* and *Vibrio harveyi*; compound **64** showed moderate activity against A-2780 cells, and antibacterial activities against *E. coli*, *Aeromonas hydrophilia,* and *V. harveyi* [51]. Botryosulfuranol A (**65**) and B (**66**) presented sulfur atoms on the α and β positions of phenylalanine-derived residues of spirocyclic centers at C-4 and C-2′ of the unprecedented spiro[isoxazolidino-cyclohexenone] scaffold. They were isolated from *Botryosphaeria mamane* and evaluated for the cell-growth inhibition of HepG2, HT-2, Caco-2 and HeLa cells. Compound **65** was the most active [52]. The investigation on mangrove endophytic fungus *Penicillium janthinellum* led to the isolation of six new penicisulfuranols, A–F (**67**–**72**), that contain sulfur atoms on both the α and β position of amino acid residues, a rare 1,2-oxazadecaline core moiety, and a rare spiro-furan ring. Compounds **67**–**69** showed cytotoxicity against the HeLa and HL-60 cell lines [53].

Epithiodiketopiperazines are cyclic dipeptides containing inter-residual polysulfide bridges between two α carbons. Outrovirin A–C (**73**–**75**), gliovirin-like compounds, were identified from *Penicillium raciborskii*. The sulfide bridge was located between α and β carbons. Compound **75** was the first reported trisulfide gliovirin-like, and it showed antifungal activity against *Botrytis cinerea* and *Verticillium dahliae* [54].

#### 3.1.7. Other Nitrogen-Containing Compounds (Amides and Amines)

The fermentation of endophytic fungus *Trichoderma atroviride* S361 led to the isolation of a pair of N-furanone amide enantiomers, (–)-trichodermadione A (**76**) and (+)–trichodermadione A (**77**). The two enantiomers did not show activity in anti-inflammatory assay or cytotoxicity test against human prostate cancer cell lines DU-145 and PC-3 [55]. The culture of *Paraconiothynium brasiliense* produced four brasilamides K–N (**78**–**81**), with rare bergamotane sesquiterpenoids with 4-oxatricyclo (3.3.1.0^2,7^) nonane (in **78)** and 9–oxatricyclo (4.3.0.0^4,7^) nonane (in **79**–**81**) skeletons. These compounds were tested against eight human tumor cell lines but did not show detectable cytotoxicity [56]. All these compounds possess a functionalized pentanamide moiety linked to a cyclic unit by a C–N or C–C bond.

**Figure 7:**
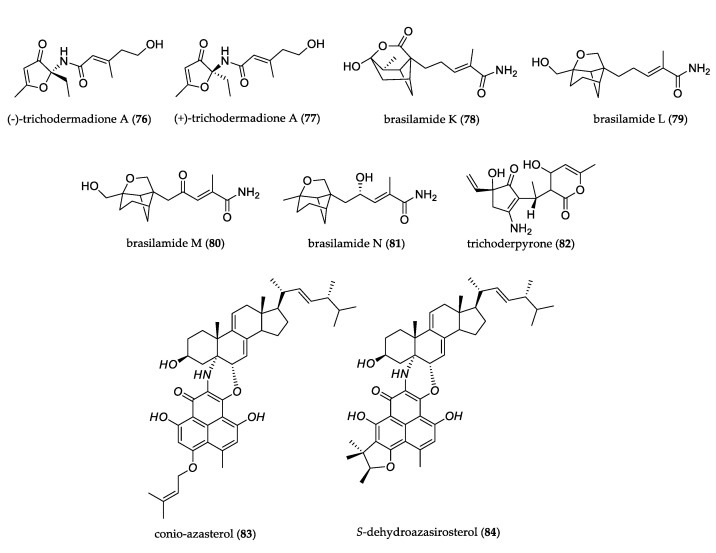
Structure of other nitrogen-containing compounds.

Compound trichoderpyrone (**82**) was isolated from *Trichoderma gamsii*. It contains a unique cyclopentenone–pyrone hybrid skeleton. This compound displayed weak cytotoxicity against the A-549, HepG2, and HeLa cancer cell lines [57]. Conio-azasterol (**83**) and *S*-dehydroazasirosterol (**84**) were isolated from *Coniothyrium cereale,* which represented two unusual nitrogen-containing compounds with a sterol portion condensed via two bonds to phenalenone derivatives [58]. Figure 7 shows the structure of other uncommon nitrogen-containing compounds.

#### 3.1.8. Other Types of Alkaloids

**Figure 8:**
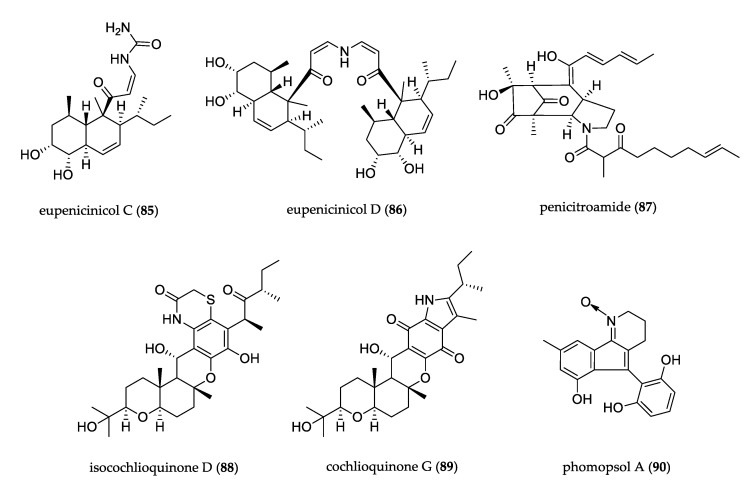
Structure of other alkaloids.

The structures of other uncommon alkaloids are shown in Figure 8. The fermentation of endophytic fungus *Eupenicillium* sp. by epigenetic stimulation led to the enhanced production of new decalin-containing eupenicicols C (**85**) and D (**86**), which displayed antimicrobial activity against *S. aureus* and cytotoxicity against the THP-1 cell line [59]. Penicitroamide (**87**) was isolated from the culture of *Penicillium* sp.; its structure contains a bicyclo[3.2.1]octane core with a high degree of carbonylization, and it displayed antibacterial activity against *Erwinia carotovora* and *Sclerotium rolfsii* [60]. The liquid culture of *Bipolaris sorokiniana* resulted in the isolation of isocochlioquinone D (**88**) and cochlioquinone G (**89**). Compound **88** featured a rare benzothiazin-3-one moiety, and compound **89** is the first example of cochlioquinones bearing an indole-4,7-dione fragment. Compounds **88** and **89** showed cytotoxic effects against the SF-268, MCF-7, NCI-H460 and HepG-2 cell lines [61]. Phomopsol A (**90**) was isolated from *Phomopsis* sp. and presented a highly oxidized polyketide containing the unique 3,4-dihydro-2*H*-indeno[1,2-*b*]pyridine 1-oxide motif. The compound showed neuroprotective effects against corticosterone-induced injury in PC12 cells [62].

### 3.2. Peptides

**Figure 9:**
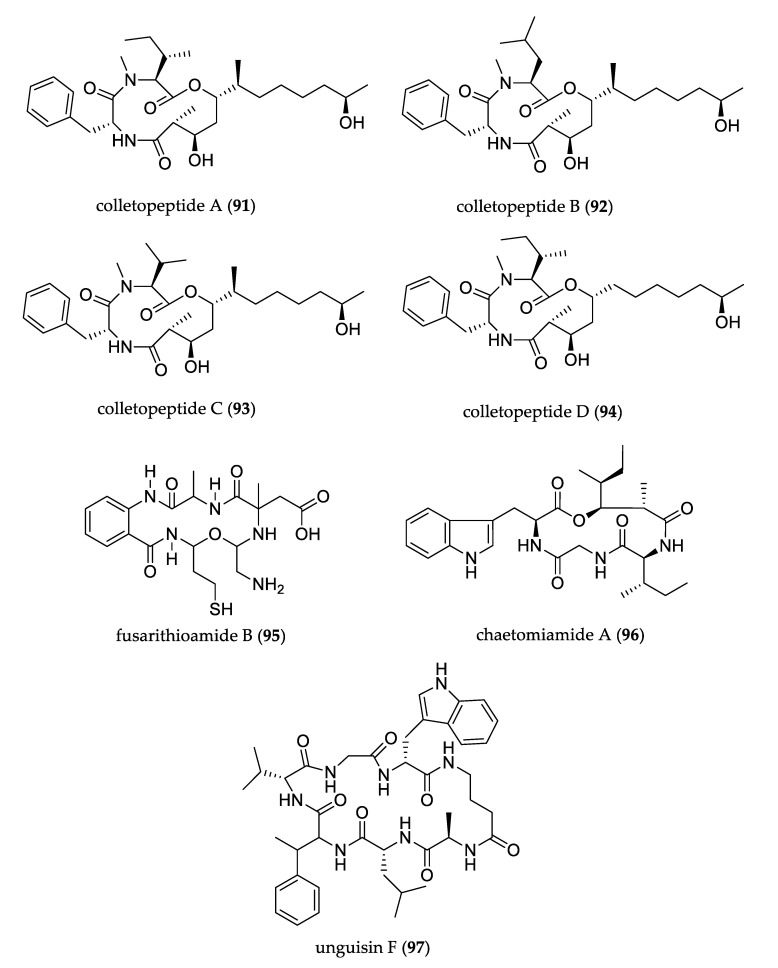
Structure of peptides.

Four new hybrid peptide–polyketide cyclic depsipeptides, colletopeptides A–D (**91**–**94**), were isolated from the culture of endophytic fungus *Colletotrichum sp*. Their structure featured a rare natural 12-membered cyclic tridepsipeptide containing a 3,5,11-trihydroxy-2-methyl dodecanoic acid unit that represented the first cyclic depsipeptide from the *Colletotrichum* genus. These compounds exhibited anti-inflammatory activity by inhibiting the production of nitric oxide in RAW264.7 macrophages induced by lipopolysaccharide (LPS); compound **91** also inhibited the production of inflammatory factors IL-6 and TNF-α, and decreased the phosphorylation of NF-κB-associated proteins IκBα and p65 [63]. Fusarithioamide B (**95**), a new aminobenzamide derivative, was isolated from *Fusarium chlamydosporium* and showed selective antifungal activity against *C. albicans,* and moderate activity against *Geotrichum candidum*. Moreover, it displayed high antibacterial potential towards *E. coli*, *B. cereus,* and *S. aureus*, a selective and potent effect towards BT-549, MCF-7, SKV-3 and HCT-116, and moderate activity towards the KB and SK-MEL cell lines [64]. Rare depsipeptide chaetomiamide A (**96**) was isolated from *Chaetomium* sp. and presented a skeleton with a 13-membered ring system [65]. Unguisin E (**97**) was obtained from cultures of *Mucor irregularis*; its structure featured a new γ-aminobutyric acid-containing cyclic peptide. It did not show biological activity in an antibacterial assay [66]. Figure 9 shows the structures of uncommon peptides.

### 3.3. Terpenoids

#### 3.3.1. Sesquiterpenoids

**Figure 10:**
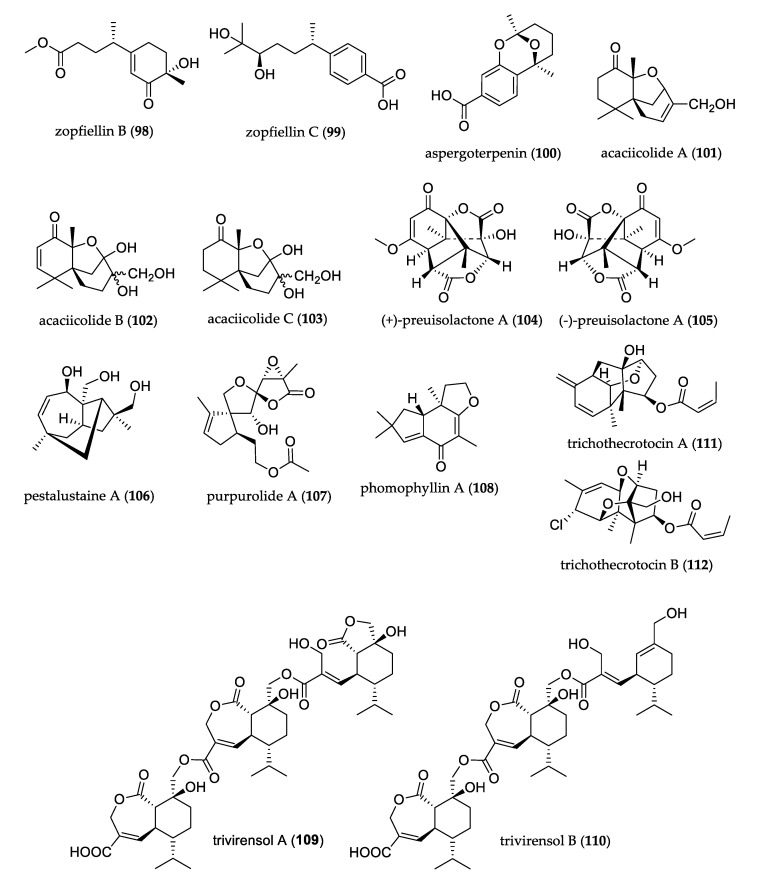
Structure of sesquiterpenoids.

The structures of uncommon sesquiterpenoids are shown in Figure 10. A culture of endophytic fungus *Zopfiella* sp. led to the isolation of undescribed bisabolane sesquiterpenoids zopfiellin B (**98**) and C (**99**); the former is a rare trinor-bisabolane sesquiterpenoid, and the latter possesses an unusual aromatic core. These compounds displayed mild cytotoxicity against the A-549, MCF-7, and HeLa cell lines [67]. Aspergoterpenin A (**100**), isolated from *Aspergillus versicolor*, is the first example of a ketal-bridged-ring part in the degraded natural bisabolane-type sesquiterpene structure. This compound displayed antimicrobial activity against *Erwinia carotovora* sub sp. [68].

Among obtained compounds from the culture of *Pseudolagarobasidium acaciicola*, acaciicolides A–C (**101**–**103**) possess a novel tricyclic ring system and did not show cytotoxic activity [69].

Norsesquiterpene enantiomers (+)–preusilactone A (**104**) and (–)–preusilactone A (**105**) were isolated from *Preussia isomera*, and featured an unprecedented 6/5/5/5/5 pentacyclic scaffold consisting of a caged tricyclo[4.4.0^1,6^.0^2,8^]decane carbon skeleton and two γ-lactone rings. Compounds **104** and **105** showed antibacterial activity against *Micrococcus luteus* [70]. Pestalustaine A (**106**) was isolated from *Pestalotiopsis adusta;* it is a unique sesquiterpene with an unusual 5/6/7-fused tricyclic ring system, and it exhibited weak-to-moderate cytotoxic activity against the HeLa, HCT-116, and A-549 cell lines [71]. Purpurolide A (**107**), a sesquiterpene lactone isolated from *Penicillium purpurogenum*, possesses a rarely encountered 5/5/5 spirocyclic skeleton; it displayed potent inhibition against pancreatic lipase [72]. Among compounds obtained from the culture of *Phomopsis* sp., phomophyllin A (**108**) is the first naturally occurring sesquiterpenoid containing an unusual 2,3-*seco*-protoilludane carbon scaffold. Compound **108** displayed β-site amyloid precursor protein-cleaving enzyme 1 (BACE1) inhibitory activity [73]. Trivirensol A (**109**) and B (**110**), isolated from endophyte *Trichoderma virens*, are unprecedented sesquiterpene trimers with three subunits connected by two ester bonds; both showed strong inhibitory activities against phytopathogenic fungi such as *Penicillium italicum*, *Fusarium oxysporum*, *Fusarium graminearun*, *Colletotrichum musae,* and *Colletotrichum gloeosporioides* [74]. Two sesquiterpenoids derived from the trichodiene precursor, trichothecrotocin A (**111**) and B (**112**), were isolated from potato endophytic fungus *Trichothecium crotocinigenum*. Compounds **111** and **112** are unusual trichothecenes that showed potent inhibitory effects against *Alternaria solani* and *Fusarium oxysporum* [75].

#### 3.3.2. Diterpenoids and Sesterterpenoids

**Figure 11:**
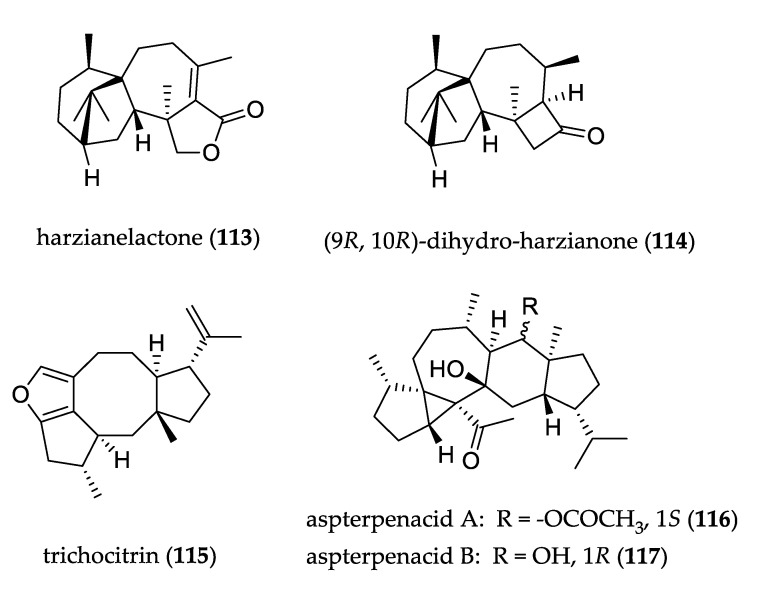
Structure of diterpenoids and sesterterpenoids.

The structures of uncommon diterpenoids and sesterterpenoids are shown in Figure 11. Harzianelactone (**113**) and (9*R*, 10*R*)-dihydro-harzianone (**114**), two harziane diterpenoids isolated from *Trichoderma* sp., contain unique 4/5/6/7 tetracyclic carbon rings. Compound **114** exhibited cytotoxicity against the HeLa and MCF-7 cell lines [76]. A new diterpene, trichocitrin (**115**), was isolated from alga-endophytic fungus *Trichoderma citrinoviride*. It represents the first furan-bearing fusicoccane diterpene derived from *Trichoderma* and it exhibited inhibition against *E. coli* [77].

Two new sesterterpenoids, aspterpenacids A (**116**) and B (**117**), featured an unusual pentacarbocyclic 5/3/7/6/5 ring system and were isolated from *Aspergillus terreus*. Both compounds were tested for antibacterial and cytotoxicity activity, but neither exhibited significant results [78].

#### 3.3.3. Meroterpenoids

**Figure 12:**
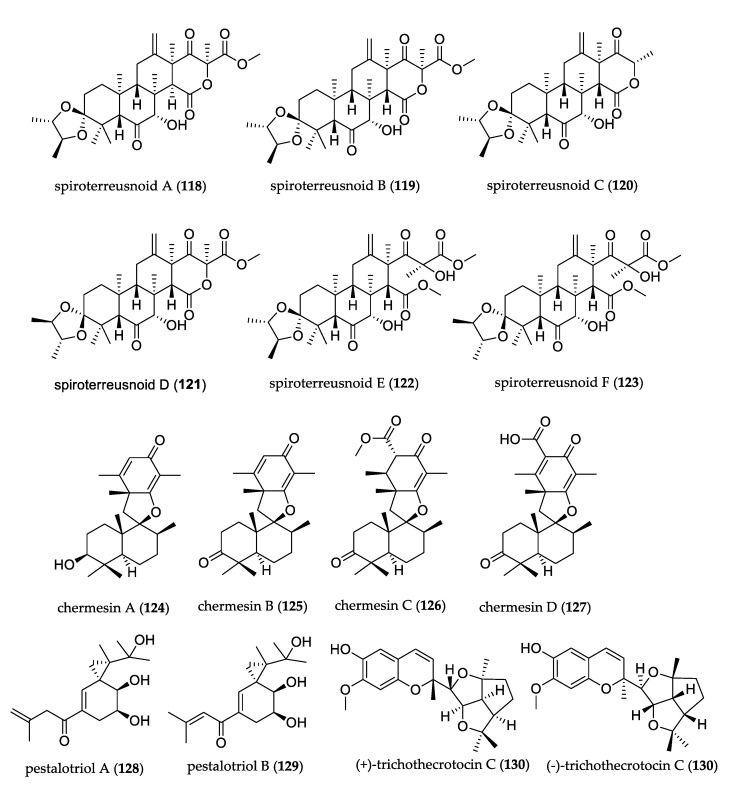

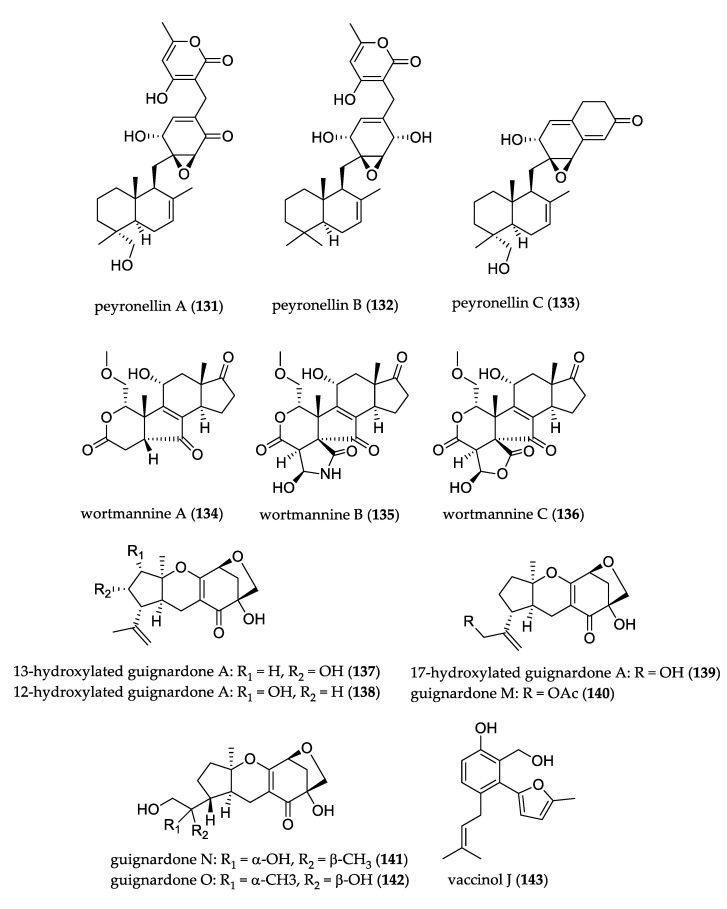

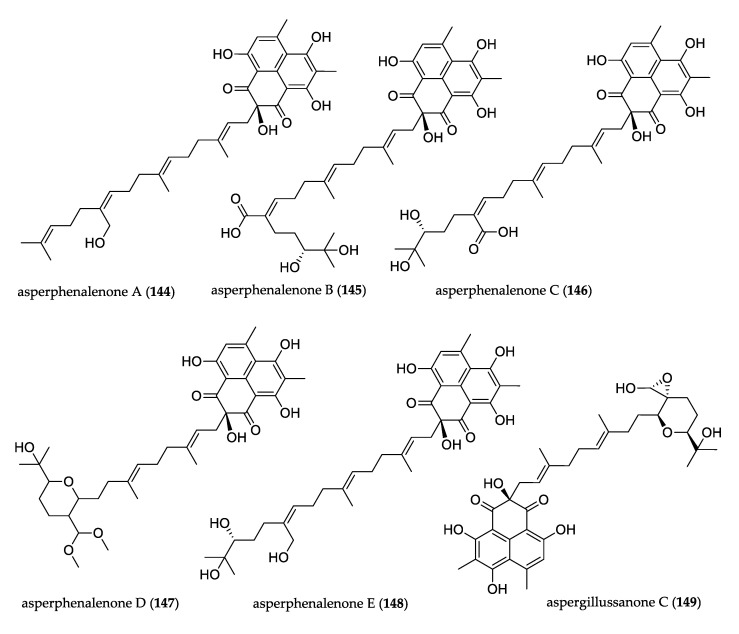
Structure of meroterpenoids.

Figure 12 shows the structures of uncommon meroterpenoids. Among compounds isolated from *Aspergillus terreus*, six new meroterpenoids, spiroterreusnoids A–F (**118**–**123**), were characterized. Their structures featured spiro-dioxolane-containing adducts bearing 3,5-dimethylorsellinic acid-based meroterpenoid and 2,3-butanediol moieties. These compounds exhibited potential abilities in inhibiting BACE1 and acetylcholinesterase (AchE) that might provide a new template for the development of new anti-Alzheimer´s disease drugs [79]. Chermesins A–D (**124**–**127**) were isolated from *Penicillium chermesinum* and represent the first report of spiromeroterpenoids that contain a drimane-type sesquiterpene skeleton with a rare cyclohexa-2,5-dienone unit. Compounds **124** and **125** exhibited antibacterial activity against *Micrococcus luteus* [80]. Pestalotriols A (**128**) and B (**129**) were isolated from *Pestalotiopsis fici,* and their structures presented a unique spiro[2.5]octane skeleton; compound **128** incorporated a spiro[2.5]oct-4-ene core that both connected to the 3-methylbut-3-enal unit at C-10 and joined to 2-hydroxypropan-2-yl at C-3. Only compound **127** showed weak cytotoxicity against HeLa cells [81].

The racemate mixture of (±)–trichothecrotocin C (**130**) was isolated from *Trichothecium crotocinigenum,* has a novel 6/6–5/5/5 fused ring system, showed potent inhibitory effects against *Alternaria solani*, *Fusarium oxysporum,* and weakly inhibited *Phytophthora infestans* and *Rhizoctonia solani* [75]. Peyronellins A–C (**131**–**133**) were isolated from *Peyronellea coffeae-arabicae* and represent unusual polyketide–sesquiterpene metabolites. Only compound **131** showed antiproliferative activity against the A-2780 and A-2780 CisR cancer cell lines [82].

The culture of *Talaromyces wortmanni* resulted in the isolation of wortmannin scaffold derivatives, all of which possess an unusual five-membered B ring as in wortmannin A (**134**); wortmannin B (**135**) and C (**136**), additional to ring B, have a lactam and a lactone E-ring, respectively. Compounds **134**–**136** were evaluated for cytotoxicity but showed no activity [83]. Among compounds isolated from the culture of *Guignardia* sp., a series of structurally similar meroterpenoids to uncommon tricycloalternarenes (**137**–**142**) were characterized to be containing an additional tetrahydrofuran ring. Compound **137** exhibited moderate antibacterial activity against *Pseudomonas aeruginosa*, while **141** showed prominent inhibition on the growth of *C. albicans* [84]. Vaccinol J (**143**) was isolated from the culture of *Pestalotiopsis vaccinni,* and it represents the first example of salicyloid derivative containing 2-methylfuran moiety. Compound **143** exhibited in vitro antienterovirus 71 activity that was 5.7 times greater than that of assayed positive-control ribavirin [85]. Asperphenalenones A–E (**144**–**148**) were isolated from *Aspergillus* sp. of the *Kadsura longipedunculata* plant. Compounds **144**–**148** represent an unusual linear diterpene derivative linked to a phenalenone derivative via a C–C bond. Compounds **144** and **147** exhibited anti-HIV activity [86]. Another example of a phenalenone derivative was aspergillussanone C (**149**), obtained from *Pinellia ternata* endophyte *Aspergillus* sp. It possesses differentiated acyclic diterpenoid oxidations resulting in an oxa-heterocyclic ring. Compound **149** did not show antimicrobial activity [87].

### 3.4. Polyketides

**Figure 13:**
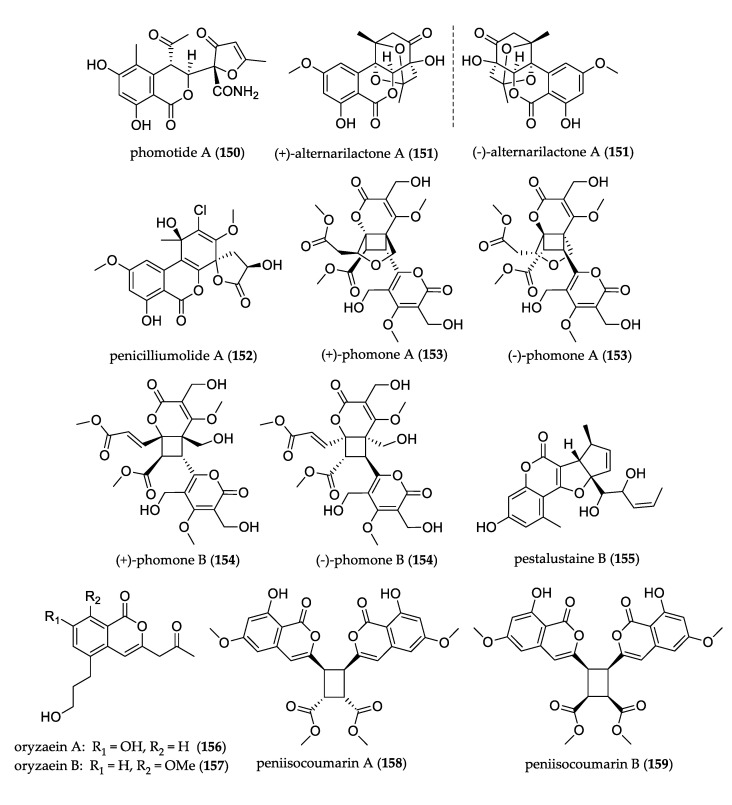

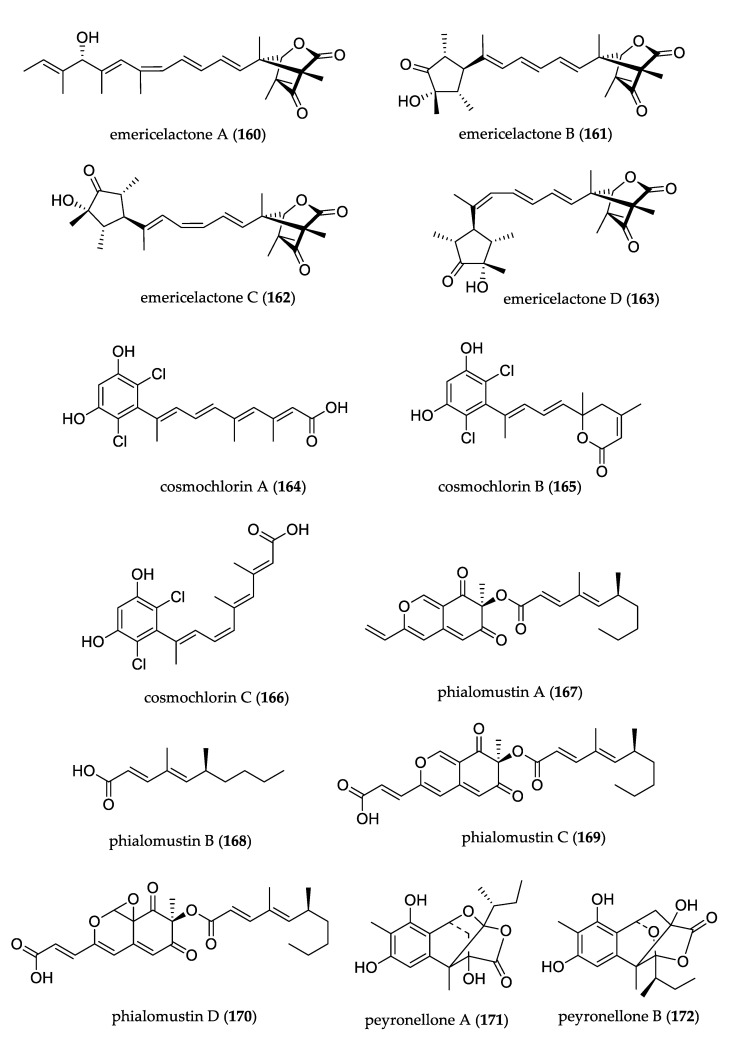

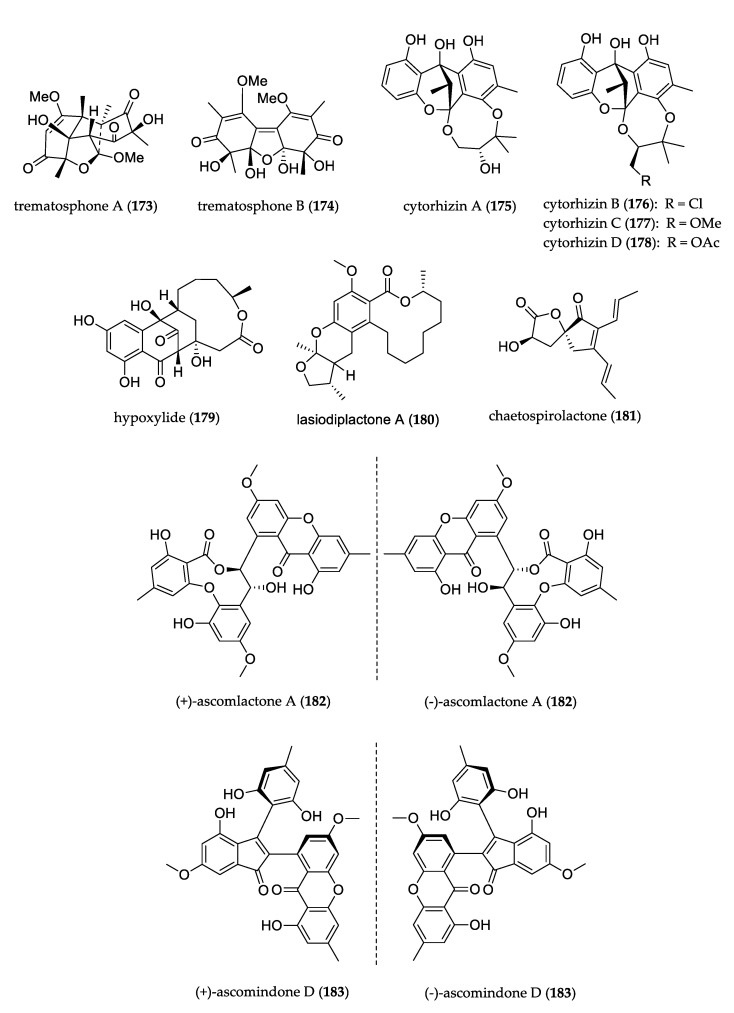

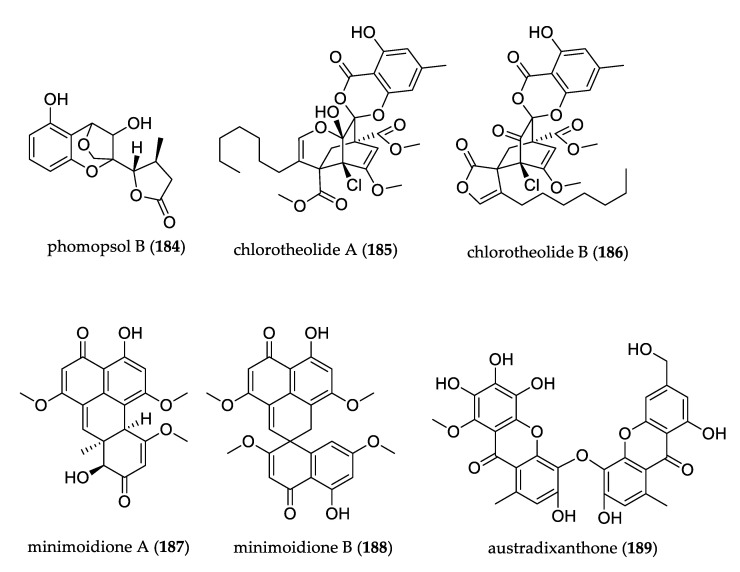
Structure of polyketides.

Figure 13 shows the structures of uncommon polyketides. Phomotide A (**150**), isolated from endophyte *Phomopsis* sp., is the first polyketide featuring an unprecedented C_12_–C_6_ carbon skeleton. The compound was tested for antibacterial activity but did not show any growth inhibition [88]. A pair of enantiomers, (+)– and (–)–alternarilactone A (**151**), were isolated from *Alternaria* sp. and represent the first example of dibenzo-α-pyrones bearing a diepoxy-cage-like moiety. This pair possessed weak protective activity against corticosterone-induced apoptosis in PC12 cells and did not show significant antibacterial or antifungal effects against some Gram-positive or -negative pathogens [89]. Penicilliumolide A (**152**) was obtained from the culture of *Penicillium chermesinum*, and it is a novel tetracyclic polyketide whose structure is uniquely spiro-attached with a γ-lactone ring. The compound did not show cytotoxicity activity [90].

A pair of enantiomers, (±)–phomone A (**153**) and (±)–phomone B (**154**), were isolated from the culture of *Phoma* sp., and both represent the first examples of 6-α,β-unsaturated ester-2-pyrone dimers; compound **153** also possessed a novel 6/4/5/6 tetracyclic ring system. Both compounds did not show cytotoxicity against three human cancer cells, but the acetylated product of **153** showed moderate cytotoxicity against the HL-60 cell line [91].

Pestalustaine B (**155**) is an unprecedented coumarin derivative bearing a 6/6/5/5-fused tetracyclic ring system isolated from *Pestalotiopsis adusta*, and it showed weak-to-moderate cytotoxic activity against the HeLa, HCT-116, and A-549 cell lines [71]. Oryzaeins A (**156**) and B (**157**) were isolated among metabolites from Aspergillus oryzae, and represented the first example of isocoumarins with an unusual 2-oxopropyl group and a rare 3-hydroxypropyl group. Both compounds displayed moderate antitobacco mosaic viral activities [92]. Among compounds isolated from *Penicillium commune*, peniisocoumarin A (**158**) and B (**159**) are unusual dimeric isocoumarins with a symmetric four-membered core at C-9/9′ and C-10/10′. These compounds showed an inhibitory effect against α-glucosidase values under 45% at 200 μM [93].

Through the OSMAC approach on *Emericella* sp., four emericelactones A–D (**160**–**163**) were isolated. Compound **160** possesses an unprecedented linear pentaene substructure that ended in an oxabicyclo[2.2.1]heptane moiety, and compounds **161**–**163** are epimers featuring a linear triene structure that ends in two cyclic moieties of oxabicyclo[2.2.1]heptane and a cyclopentan-1-one. All four compounds showed moderate antimicrobial activities against *Verticillium dahliae*, *R. solani*, *Gibberella saubinetii*, *Micrococcus lysodeikticus*, and *Salmonella typhi* [94]. Cosmochlorins A–C (**164**–**166**) were isolated from *Cosmopora vilior* and represented the first naturally occurring compounds containing a 3-(1,5-dihydroxy-2,4-dichloro)phenyl moiety. Compounds **164** and **165** partially restored the growth inhibition caused by hyperactivated Ca^2+^ signaling in mutant yeast, and showed glycogen synthase kinase (GSK)-3β inhibition activity. Additionally, compound **165** increased osteoclast formation in RAW264,7 cells compared to the receptor activator of nuclear factor-κβ ligand (RANKL) alone [95].

Phialomustins A–D (**167**–**170**) are four metabolites with an unprecedented azaphilone-derived skeleton isolated from endophyte Phialophora mustea. Compound **168** exhibited cytotoxic activity against the T47D cell line; compounds **169** and **170** showed promisory antifungal activity against C. albicans [96]. Peyronellones A (**171**) and B (**172**) are tetracyclic caged aducts of azaphilone with pyruvic acid obtained from the culture of *Peyronellaea glomerata*. Both compounds showed potent antioxidant effects, and compound **172** significantly protects hypoxia/reoxygenation (H/R)-treated human umbilical vein endothelial cells [97].

Trematosphones A (**173**) and B (**174**) are two dimers isolated from *Trematosphaeria terricola* that represent novel unique natural products. Compound **173** featured a 6/7/6/5-fused tetracyclic system with seven stereocenters and eight oxygen atoms forming groups such as carbonyl, ether, hydroxy, and methoxy, and it showed protective activity against corticosterone-induced damage in PC12 cells. Compound **174** possessed a highly axial symmetrical tricyclic structure with nine oxygen atoms and displayed no activity in the same assay [98]. Cytorhizins A–D (**175**–**178**) were obtained from *Cytospora rhizophorae*, and their structures represent a novel family of functionalized benzophenol derivatives with a cage-like scaffold that comprised a transannular benzophenol core and a highly oxygenated hemiterpene unit, and 6/6/5/6/7 or 6/6/5/6/8 fused ring systems. The compounds were evaluated for cytotoxicity against cancer cell lines, and compounds **176** and **178** showed weak cytoxicity against the HepG-2, MCF-7, SF-268, and A-549 cell lines [99]. Hypoxylide (**179**) was isolated from *Annulohypoxylon* sp. and possesses a trihydroxynaphthalene-dione moiety fused to a decalactone ring. Compound **179** did not show cytotoxic and antibacterial activity [100]. Lasiodiplactone A (**180**) was isolated from *Lasiodiplodia theobromae* and it is the first lactone with a 12/6/6/5 tetracyclic system (12-membered β-resorcylic acid lactone) with a pyran and a furan ring; it showed anti-inflammatory activity by inhibiting NO production in LPS-activated RAW264.7 cells [101]. Chaetospirolactone (**181**) was isolated from *Chaetomium* sp. featuring spiro-lactone with a 1-oxaspiro[4.4]non-7-ene-2,6-dione skeleton. Compound **181** was tested in an acetylcholinesterase inhibitory assay but did not show activity [102]. A pair of enantiomeric polyketide lactone dimers, (+)– and (–)–ascomlactone A (**182**), were isolated from mangrove-derived *Ascomycota* sp. Their structures possessed a nine-membered lactone ring between the monomers in an unprecedented polymerization way. Both enantiomers showed significant inhibition effects against α-glucosidase [103]. Another pair of enantiomers, (+)– and (–)– ascomindone D (**183**), were obtained from the same *Ascomycota* sp. These compounds are 2,3-diaryl indone derivatives and showed potential anti-inflammatory effects by inhibiting the production of NO in LPS-activated RAW246.7 cells [104]. Phomopsol B (**184**) was isolated together with the previously mentioned polyketide-derived alkaloid phomopsol A (**90**) from *Phomopsis* sp., and presented a novel 3,5-dihydro-2H-2,5-methanobenzo[e][1,4]dioxepine motif. Compound **184** did not show a neuroprotective effect [62].

Chlorotheolides A (**185**) and B (**186**) were isolated from *Pestalotiopsis theae*, which possesses spiroketals with [4,7]methanochromene and dispirotrione skeletons. Compound **186** showed an antiproliferative effect against HeLa and induced an autophagic process in the cells [105].

Minimoidiones A (**187**) and B (**188**) possess novel skeletons of benzo[de]anthracenedione and spiro[naphthalenephenalene]dione; they were obtained from *Preussia minimoides* and inhibited yeast α-glucosidase (αGHY) [106].

The axenic culture of *Aspergillus austroafricanus* produces highly oxygenated heterodimeric xanthone derivative austradixanthone (**189**). It did not show cytotoxicity or antibacterial activity [107].

### 3.5. Steroids

**Figure 14:**
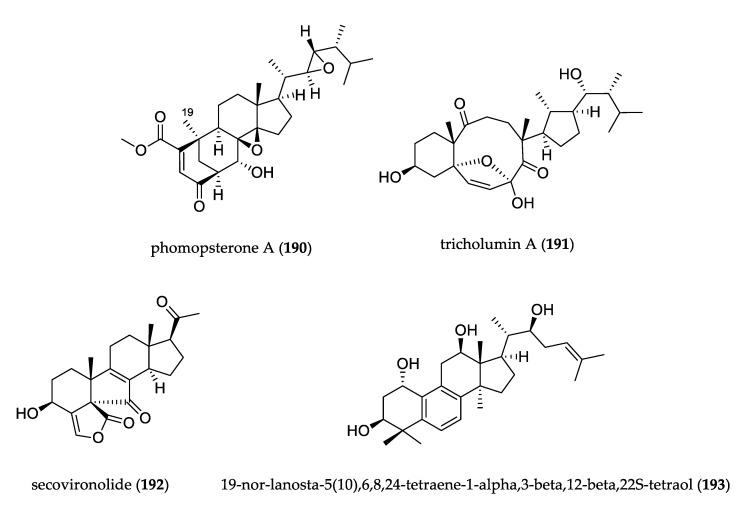
Structure of steroids.

The structures of uncommon steroids are shown in Figure 14. Phomopsterone A (**190**), isolated from endophytic fungus Phomopsis sp., is a highly oxygenated ergostane-type steroid possessing a unique bicyclo[3.3.1]nonane motif with an α-oriented CH_3_-19 group. This extremely unusual feature provides new insight into steroid biosynthesis. This compound was tested for in vitro anti-inflammatory activity, but did not show considerable activity [108]. Tricholumin A (**191**) has an unprecedented carbon skeleton of a highly transformed ergosterol derivative isolated from alga-endophytic Trichoderma asperellum and exhibited inhibition against some pathogenic microbes (*V. harveyi*, *V. splendidus*, and *Pseudoalteromonas citrea*), marine phytoplankton species (*Chattonella marina*, *Heterosigma akashiwo*, *Karlodinium veneficum*, *Prorocentrum donghaiense*), and antifungal activity against *Glomerella cingulata* [109]. Secovironolide (**192**) was isolated from *Talaromyces wortmannii* and represents the first example of a furanosteroid with a five-membered B-ring in the carbon scaffold. The compound showed weak inhibitory activity in a monoamine oxidase assay [110]. Lanostanoid (**193**) was isolated from *Diaporthe* sp. It presented an aromatic B ring with an unusual loss of CH_3_-19 during the aromatization of the B ring, and hydroxylation at C-1, C-3, C-12, and C-22. This compound is the second reported natural lanostane/cucurbitane derivative with an aromatized B ring. Compound **193** showed pronounced antibacterial efficacy against *S. aureus*, *E. coli*, *B. subtilis*, *P. aeruginosa*, and *S. pyogenes* [111].

### 3.6. Analysis of Neighbor Net and Diversity of Endophytic Fungi Producing Secondary Metabolites with Uncommon Structures

**Figure 15:**
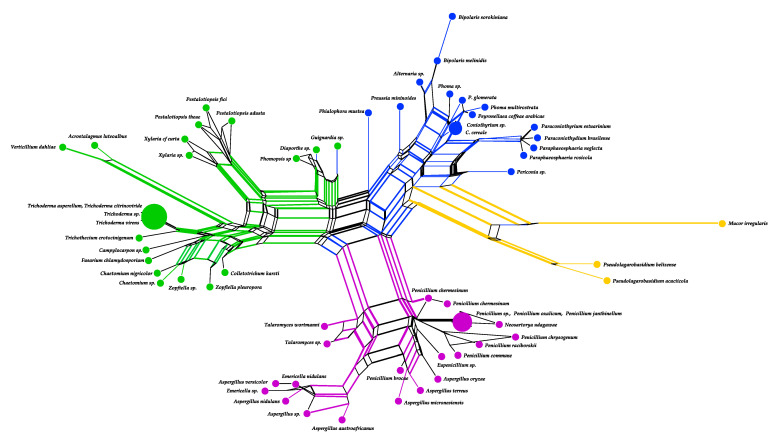
Analysis of neighbor net using nucleotide sequences of the internal transcribed spacer (ITS1) of the ribosomal gene of 5 classes of endophytic fungi: Eurotiomycetes in pink; Sordariomycetes in green; Dothideomycetes in blue; and Mucoromycetes and Agaricomycetes in yellow. Larger circles indicate the number of species with similar sequences in the network. GenBank accession numbers and the names of the analyzed species are in Supplementary Table S1.

The analyzed fungal species in the present review belong to classes Eurotiomycetes (pink), Sordariomycetes (green), Dothideomycetes (blue), and a minority corresponds to classes Mucoromycetes and Agaricomycetes (Figure 15, yellow). Phylogenetic-network analysis using the internal transcriber spacer (ITS1) of the ribosomal gene indicated that the production of secondary metabolites with uncommon structures is a widely distributed capacity within the seven classes of endophytic fungi analyzed here. Furthermore, endophytic fungal species with wide genetic divergence can generate similar compounds with different uncommon structures. For instance, alkaloids with novel structures were reported in a large number of species in these classes of fungi (Table S1, Figure 15). However, species belonging to the Eurotiomycetes class *Aspergillus* and *Penicillium* can biosynthesize a greater variety of these compounds, while the cytochalasin type were mainly isolated from species belonging to the Sordariomycetes class [112,113].

## 4. Discussion

Endophytic fungi producing structurally uncommon compounds listed in the present review were obtained mainly from two hosts: medicinal (37%) and mangrove (24%) plants. These values confirmed the current trend of studying medicinal plant-derived-endophytes because it is known that they could have the ability to produce the same metabolites as the host plant [10]. Due to the unique characteristic of the ecosystem, mangrove endophytes have been attractive to the pharma industry, as they produce metabolites that are structurally unique and biologically active [114]. From the 39 different genera reviewed, the majority of references were related to species of *Aspergillus* (13), *Penicillium* (11), *Trichoderma* (6), *Phomopsis* (5), *Chaetomium*, *Pestalotiopsis, Xylaria* (all three with 4) and *Talaromyces* (3). From the 84 samples of endophytic fungi mentioned in this review, only 57 have been determined to the level of species.

The 202 structures were distributed in six groups: 93 (46%) as alkaloids, 53 (26.2%) as terpenoids, 45 (22.3%) as polyketides, 7 (3.5%) as peptides, and 4 (2%) as steroids. Around 28% were isolated from two main genera, *Aspergillus* and *Penicillium*, with 31 and 25 compounds, respectively. For most of these compounds, a biological activity has been determined, including cytotoxic, antitumor, antimicrobial, antiviral, or anti-inflammatory. Table S2 provides information about the endophytes, their host plant, the culture parameters that lead to the production of the uncommon compounds and the biological activity reported.

There is the possibility that these structurally uncommon compounds were produced due to the long culture periods where the fungus starts to consume the first round of produced secondary metabolites to survive starving or a low-resources condition to produce novel and uncommon structures or skeletons via intra- or extramolecular coupling, fusion, or cyclization, such as those described above, in some cytochalasans or polyketides.

Based on the analyzed data, genes mainly involved in the biosynthesis of alkaloids are conserved in a wide range of endophytic fungal species. However, the synthesis of a specific uncommon structure is probably related to genetic variability and a particular epigenetic aspect of each particular species. Therefore, future research should be focused on the characterization of the biosynthetic gene cluster (BGC), responsible for producing these uncommon structures, to enhance biological synthesis as a tool to produce metabolites of interest. This review demonstrates the importance of endophytic fungi in the production of exclusive metabolites with biotechnological applications. Furthermore, it adequately highlights the value of biological resources for the development of biotrade.

## Data Availability

Not applicable.

## Supplementary Materials

The following are available online at https://www.mdpi.com/article/10.3390/jof7070570/s1. Table S1: Endophytic fungi examined in this study and access numbers in GenBank of their respective sequences of the internal transcribed spacer (ITS1) of the ribosomal gene. Table S2: Compilation of culture conditions for each fungi and biological activity reported for compounds reviewed.
