# Supplementary material for: Structurally Uncommon Secondary Metabolites Derived from Endophytic Fungi
Source: J Fungi (Basel). 2021 Jul 17;7(7):570. doi: 10.3390/jof7070570 (PMC8308102; doi:10.3390/jof7070570)
Supplement: Supplementary file 1 [file jof-07-00570-s001.zip › jof-1307300-supplementary.pdf]

Table S1. Endophytic fungi examined in this study and access number in GenBank of their respective sequences of the internal transcribed spacer (ITS1) of the ribosomal gene

No.	*Species*	Compounds	Compounds type	Family	Order	Class	Division	Access number GenBank
1	*Aspergillus micronesiensis*	Cytochalasans	Alkaloids	Trichocomaceae	Eurotiales	Eurotiomycetes	Ascomycota	MH938722.1
2	*Aspergillus terreus*	Indole Alkaloids	Alkaloids	Trichocomaceae	Eurotiales	Eurotiomycetes	Ascomycota	KJ439155
2	*Aspergillus terreus*	Diterpenoids and Sesterterpenoids /Meroterpenoids	Terpenoids	Trichocomaceae	Eurotiales	Eurotiomycetes	Ascomycota	KJ439155
3	*Aspergillus nidulans*	Indole Alkaloids	Alkaloids	Trichocomaceae	Eurotiales	Eurotiomycetes	Ascomycota	KP126907.1
4	*Aspergillus sp.*	Pyridone and pyridinyl derivatives / Diketopiperazine derivatives /	Alkaloids /Terpenoids	Trichocomaceae	Eurotiales	Eurotiomycetes	Ascomycota	MN428770.1
5	*Aspergillus versicolor*	Sesquiterpenoids	Terpenoids	Trichocomaceae	Eurotiales	Eurotiomycetes	Ascomycota	NR_131277.1
6	*Aspergillus oryzae*	Polyketides	Polyketides	Trichocomaceae	Eurotiales	Eurotiomycetes	Ascomycota	KM999948.1
7	*Aspergillus austroafricanus*	Polyketides	Polyketides	Trichocomaceae	Eurotiales	Eurotiomycetes	Ascomycota	KP013121.1
8	*Penicillium chrysogenum*	Cytochalasans	Alkaloids	Trichocomaceae	Eurotiales	Eurotiomycetes	Ascomycota	KX777253
9	*Penicillium oxalicum*	Indole Alkaloids /Pyridone and pyridinyl derivatives	Alkaloids	Trichocomaceae	Eurotiales	Eurotiomycetes	Ascomycota	KP768466.1
10	*Penicillium raciborskii*	Diketopiperazine derivatives	Alkaloids	Trichocomaceae	Eurotiales	Eurotiomycetes	Ascomycota	GQ266152.1
11	*Penicillium brocae*	Diketopiperazine derivatives	Alkaloids	Trichocomaceae	Eurotiales	Eurotiomycetes	Ascomycota	NR_111868.1
12	*Penicillium janthinellum*	Diketopiperazine derivatives	Alkaloids	Trichocomaceae	Eurotiales	Eurotiomycetes	Ascomycota	KM659023.1
13	*Penicillium sp.*	Other types of alkaloids	Alkaloids	Trichocomaceae	Eurotiales	Eurotiomycetes	Ascomycota	MN726655.1
14	*Penicillium purpurogeum*	Sesquiterpenoids	Terpenoids	Trichocomaceae	Eurotiales	Eurotiomycetes	Ascomycota	_
15	*Penicillium chermesinum*	Meroterpenoids	Terpenoids	Trichocomaceae	Eurotiales	Eurotiomycetes	Ascomycota	KR076794.1
16	*Penicillium chermesinum*	Polyketides	Polyketides	Trichocomaceae	Eurotiales	Eurotiomycetes	Ascomycota	NR_121310.1
17	*Penicillium commune*	Polyketides	Polyketides	Trichocomaceae	Eurotiales	Eurotiomycetes	Ascomycota	MG193677.1
18	*Eupenicillium sp.*	Other types of alkaloids	Alkaloids	Trichocomaceae	Eurotiales	Eurotiomycetes	Ascomycota	HQ649854.1
19	*Emericella nidulans*	Isoindolone derivatives	Alkaloids	Trichocomaceae	Eurotiales	Eurotiomycetes	Ascomycota	KU377335.1
20	*Emericella sp.*	Polyketides	Polyketides	Trichocomaceae	Eurotiales	Eurotiomycetes	Ascomycota	JQ268604.1
21	*Talaromyces sp.*	Pyrrolidone derivatives	Alkaloids	Trichocomaceae	Eurotiales	Eurotiomycetes	Ascomycota	KP975419
22	*Talaromyces wortmanni*	Meroterpenoids	Terpenoids	Trichocomaceae	Eurotiales	Eurotiomycetes	Ascomycota	NR_172039.1
22	*Talaromyces wortmanni*	Steroids	Steroids	Trichocomaceae	Eurotiales	Eurotiomycetes	Ascomycota	NR_172039.1
23	*Neosartorya udagawae*	Indole Alkaloids	Alkaloids	Trichocomaceae	Eurotiales	Eurotiomycetes	Ascomycota	KT326154.1
24	*Phialophora mustea*	Polyketides	Polyketides	Herpotrichiellaceae	Chaetothyriales	Eurotiomycetes	Ascomycota	AB190404.1

No.	*Species*	Compounds	Compounds type	Family	Order	Class	Division	Access number GenBank
25	*Phomopsis sp.*	Cytochalasans /Other types of alkaloids	Alkaloids	Diaportheaceae	Diaporthales	Sordariomycetes	Ascomycota	KU533636
25	*Phomopsis sp.*	Sesquiterpenoids	Terpenoids	Diaportheaceae	Diaporthales	Sordariomycetes	Ascomycota	KU533636
25	*Phomopsis sp.*	Polyketides	Polyketides	Diaportheaceae	Diaporthales	Sordariomycetes	Ascomycota	KU533636
25	*Phomopsis sp.*	Steroids	Steroids	Diaportheaceae	Diaporthales	Sordariomycetes	Ascomycota	KU533636
26	*Phoma multirostrata*	Cytochalasans	Alkaloids	Diaportheaceae	Diaporthales	Sordariomycetes	Ascomycota	EF585392
27	*Trichoderma sp.*	Diterpenoids and Sesterterpenoids	Terpenoids	Hypocreaceae	Hypocreales	Sordariomycetes	Ascomycota	MT079279.1
28	*Trichoderma gamsii*	Diketopiperazine derivatives /other nitrogen-containing compounds	Alkaloids	Hypocreaceae	Hypocreales	Sordariomycetes	Ascomycota	_
29	*Trichoderma atroviride*	Other nitrogen containing compounds	Alkaloids	Hypocreaceae	Hypocreales	Sordariomycetes	Ascomycota	HQ449987
30	*Trichoderma virens*	Sesquiterpenoids	Terpenoids	Hypocreaceae	Hypocreales	Sordariomycetes	Ascomycota	MN102106
31	*Trichoderma citrinoviride*	Diterpenoids and Sesterterpenoids	Terpenoids	Hypocreaceae	Hypocreales	Sordariomycetes	Ascomycota	KT259441
32	*Trichoderma asperellum*	Steroids	Steroids	Hypocreaceae	Hypocreales	Sordariomycetes	Ascomycota	NR_130668.1
33	*Trichoderma crotocinigenum*	sesquiterpenoids	Terpenoids	Hypocreaceae	Hypocreales	Sordariomycetes	Ascomycota	_
34	*Chaetomium sp.*	Peptides / Polyketides	Peptides	Chaetomiaceae	Sordariales	Sordariomycetes	Ascomycota	KP171518
35	*Chaetomium globosum*	Cytochalasans	Alkaloids	Chaetomiaceae	Sordariales	Sordariomycetes	Ascomycota	_
36	*Chaetomium nigricolor*	Cytochalasans	Alkaloids	Chaetomiaceae	Sordariales	Sordariomycetes	Ascomycota	KX146510.1
37	*Colletotrichum sp.*	Pyrrolidone derivatives	Alkaloids	Glomerellaceae	Glomerellales	Sordariomycetes	Ascomycota	_
37	*Colletotrichum sp.*	Peptides	Peptides	Glomerellaceae	Glomerellales	Sordariomycetes	Ascomycota	_
38	*Colletotrichum genus*	Peptides	Alkaloids	Glomerellaceae	Glomerellales	Sordariomycetes	Ascomycota	
39	*Colletotrichum salsolae*	Pyrrolidone derivatives	Alkaloids	Glomerellaceae	Glomerellales	Sordariomycetes	Ascomycota	_
40	*Colletotrichum karsti*	Peptides	Peptides	Glomerellaceae	Glomerellales	Sordariomycetes	Ascomycota	KX306754.1
41	*Xylaria sp.*	Cytochalasans	Alkaloids	Xylariaceae	Xylariales	Sordariomycetes	Ascomycota	MT997271.1
42	*Xylaria cf. curta*	Cytochalasans	Alkaloids	Xylariaceae	Xylariales	Sordariomycetes	Ascomycota	KX034338.1
43	*Annulohypoxylon sp*	Polyketides	Polyketides	Xylariaceae	Xylariales	Sordariomycetes	Ascomycota	
44	*Pestalotiopsis fici*	Meroterpenoids	Terpenoids	Xylariaceae	Xylariales	Sordariomycetes	Ascomycota	DQ789388.1
45	*Pestalotiopsis adusta*	Polyketides / Sesquiquipertenoids	Polyketides	Xylariaceae	Xylariales	Sordariomycetes	Ascomycota	AY687298.1
46	*Pestalotiopsis theae*	Polyketides	Polyketides	Xylariaceae	Xylariales	Sordariomycetes	Ascomycota	KF641183
47	*Pestalotiopsis vaccinni*	Meroterpenoids	Terpenoids	Xylariaceae	Xylariales	Sordariomycetes	Ascomycota	DQ813434.1
48	*Trichothecium crotocinigenum*	Sesquiterpenoids /Meroterpenoids	Terpenoids	incertae sedis	Hypocreales	Sordariomycetes	Ascomycota	KR709188.1
49	*Diaporthe sp.*	Indolimone derivatives	Alkaloids	Diaportheaceae	Diaporthales	Sordariomycetes	Ascomycota	KR091793.1
49	*Diaporthe sp.*	Steroids	Steroids	Diaportheaceae	Diaporthales	Sordariomycetes	Ascomycota	KR091793.1
50	*Cytospora rhizophorae*	Polyketides	Polyketides	Valsaceae	Diaporthales	Sordariomycetes	Ascomycota	_
51	*Fusarium chlamydosporium*	Peptides	Peptides	Nectriaceae	Hypocreales	Sordariomycetes	Ascomycota	KY092643.1
52	*Campylocarpon sp.*	Pyridone and pyridinyl derivatives	Alkaloids	Nectriaceae	Hypocreales	Sordariomycetes	Ascomycota	KU937938
53	*Acrostalagmus luteoalbus*	Diketopiperazine derivatives	Alkaloids	Hypocreaceae	Hypocreales	Sordariomycetes	Ascomycota	MH836621
54	*Cosmospora vilior*	Polyketides		Hypocreaceae	Hypocreales	Sordariomycetes	Ascomycota	
55	*Paecilomyces variotii*	Diketopiperazine derivatives	Alkaloids	Trichocomaceae	Eurotiales	Sordariomycetes	Ascomycota	_
56	*Verticillium dahliae*	Diketopiperazine derivatives	Alkaloids	Plectosphaerellaceae	Hypocreales	Sordariomycetes	Ascomycota	GU060637.1
57	*Zopfiella sp.*	Sesquiterpenoids	Terpenoids	Lasiosphaeriaceae	Sordariales	Sordariomycetes	Ascomycota	KR154941.1
58	*Zopfiella pleuropora*	Sesquiterpenoids	Terpenoids	Lasiosphaeriaceae	Sordariales	Sordariomycetes	Ascomycota	MH859827.1

No.	*Species*	Compounds	Compounds type	Family	Order	Class	Division	Access number GenBank
59	*Phoma sp.*	Polyketides	Polyketides	Didymellaceae	Pleosporales	Dothideomycetes	Ascomycota	NR_135967.1
60	*Phoma multirostrata*	Cytochalasans	Alkaloids	Didymellaceae	Pleosporales	Dothideomycetes	Ascomycota	EF585392.1
61	*Peyronellaea coffeae-arabicae*	Meroterpenoids	Terpenoids	Didymellaceae	Pleosporales	Dothideomycetes	Ascomycota	KU318713.1
62	*Peyronellaea glomerata*	Polyketides	_	Didymellaceae	Pleosporales	Dothideomycetes	Ascomycota	KF293971.1
63	*Preussia isomera*	Sesquiterpenoids	Terpenoids	Sporomiaceae	Pleosporales	Dothideomycetes	Ascomycota	_
64	*Preussia mininoides*	Polyketides	Polyketides	Sporomiaceae	Pleosporales	Dothideomycetes	Ascomycota	KF557658.1
65	*Preussia polymorpha*	_	_	Sporomiaceae	Pleosporales	Dothideomycetes	Ascomycota	_
66	*Coniothyrium cereale*	Diketopiperazine derivatives /other nitrogen-containing compounds	Alkaloids	Phaeosphaeriacea	Pleosporales	Dothideomycetes	Ascomycota	AJ293812.1
67	*Coniothyrium sp.*	_	_	Leptosphaeriaceae	Pleosporales	Dothideomycetes	Ascomycota	KP749188.1
68	*Paraconiothyrium brasiliense*	Diketopiperazine derivatives /other nitrogen-containing compounds	Alkaloids	Didymosphaeriaceae	Pleosporales	Dothideomycetes	Ascomycota	MH862698.1
69	*Paraconiothyrium estuarinum*	_	_	Didymosphaeriaceae	Pleosporales	Dothideomycetes	Ascomycota	MH862842.1
70	*Botryosphaeria mamane*	Diketopiperazine derivatives	Alkaloids	Botryosphaeriaceae	Botryosphaeriales	Dothideomycetes	Ascomycota	_
71	*Botryosphaeria dothidea*	Diketopiperazine derivatives	Alkaloids	Botryosphaeriaceae	Botryosphaeriales	Dothideomycetes	Ascomycota	_
72	*Lasiodiplodia theobromae*	Polyketides	Polyketides	Botryosphaeriaceae	Botryosphaeriales	Dothideomycetes	Ascomycota	_
73	*Bipolaris sorokiniana*	Other types of alkaloids	Alkaloids	Pleosporaceae	Pleosporales	Dothideomycetes	Ascomycota	KF494823
74	*Bipolaris melinidis*	_	_	Pleosporaceae	Pleosporales	Dothideomycetes	Ascomycota	JN192382.1
75	*Alternaria sp.*	Polyketides	Polyketides	Pleosporaceae	Pleosporales	Dothideomycetes	Ascomycota	KX270745.1
76	*Paraphaeosphaeria neglecta*	Pyrrolidone derivatives	Alkaloids	Didymosphaeriaceae	Pleosporales	Dothideomycetes	Ascomycota	KT715703.1
77	*Paraphaeosphaeria rosicola*	_	_	Didymosphaeriaceae	Pleosporales	Dothideomycetes	Ascomycota	MG828938.1
78	*Trematosphaeria terricola*	Polyketides		Melanommataceae	Pleosporales	Dothideomycetes	Ascomycota	
79	*Periconia sp.*	Pyrrolidone derivatives	Alkaloids	Periconeaceae	Pleosporales	Dothideomycetes	Ascomycota	NR_171873.1
79	*Periconia sp.*	Cytochalasans	Alkaloids	Periconeaceae	Pleosporales	Dothideomycetes	Ascomycota	NR_171873.1
80	*Guignardia sp*	Meroterpenoids		Botryosphaeriaceae	Botryosphaeriales	Dothideomycetes	Ascomycota	JN165708.1

No.	*Species*	Compounds	Compounds type	Family	Order	Class	Division	Access number GenBank
81	*Mucor irregularis*	Indole Alkaloids/ Peptides	Alkaloids	Mucoraceae	Mucorales	Mucoromycetes	Mucoromycota	HQ891659
82	*Pseudolagarobasidium acaciicola*	Sesquiterpenoids	Terpenoids	Phanerochaetaceae	Polyporales	Agaricomicetes	Basidiomicota	KF673346
83	*Pseudolagarobasidium belizense*	_	_	Phanerochaetaceae	Polyporales	Agaricomicetes	Basidiomicota	JQ070173.1

Table S2. Compilation of culture conditions for each fungi and biological activity reported for compounds reviewed.

Ref.	Host plants, Fungi, and fermentation methodology	Compounds	Biological activity of compounds
[1]	**Host plan**t: tissue of medicinal plant *Actinidia chinensis* Planch<br><br>**Fungus:** *Zopfiella sp*<br><br>**Fermentation:** Culture medium consists of glucose (5%), pork peptone (0.15 %), yeast (0.5 %), KH_2PO_4 (0.05 %) and $MgSO_4$ (0.05 %). Initial pH was adjusted to 6.0, the fermentation was initially implemented on an Erlenmeyer flask for 6 days until the mycelium biomass reached to the maximum. Then it was transferred to rice medium for 24 °C in dark culture for 30 days. Rice medium: 75 g of rice, 75 mL of water, placed in a 250 mL Erlenmeyer flask, sterilized at 121 °C for 15 min, a total of 200 bottles.	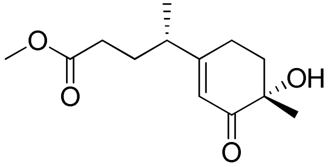 zopfiellin B (**98**) 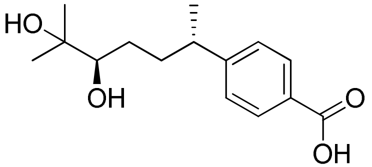 zopfiellin C (**99**)	These compounds displayed mild cytotoxicity against the A-549, MCF-7, and HeLa cell lines.
[2]	**Host plant:** mangrove plant *Kandelia candel* (L.) Druce<br><br>**Fungus:** *Colletrotrichum salsolae SCSIO 41021*<br><br>**Fermentation:** Strain stored on PDA slants at 4 °C was cultured on PDA agar plates and incubated for 7 days. Seed medium (potato 200 g, dextrose 20 g, NaCl 2.5 g, distilled water 1000 mL) in 50-mL Erlenmeyer flasks was inoculated with strain and incubated at 25 °C for 48 h on a rotating shaker (180 rpm). Production medium of 200-mL PDB in 1000 mL flasks was inoculated with 10 mL seed solution. Flasks were incubated at 25 °C under static stations and daylight. After 15 days, cultures from 100 flasks were harvested for the isolation of substances.	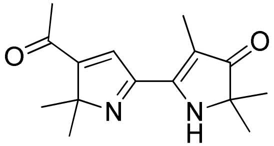 collacyclumine A (**42**)	It did not show cytotoxic activity.
[3]	**Host plant:** medicinal plant *Cephalotaxus fortune* Hook<br><br>**Fungus:** *Phomopsis* sp. CFS42<br><br>**Fermentation:** The fungus was inoculated on a PDA plate, then one colony was picked up into an Erlenmeyer flask with 200 ml YMG liquid medium to prepare seed culture. After cultivated at 28 °C, 140 rpm for one week, seed culture was inoculated in 20 Erlenmeyer with 80 g rice. The flasks was cultured at 28 °C for four weeks.	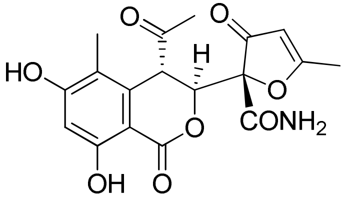 phomotide A (**150**)	The compound was tested for antibacterial activity but did not show any growth inhibition

[4]	**Host plant:** leaves of a mangrove plant *Kandelia candel*<br><br>**Fungus:** *Aspergillus* sp. SK-28<br><br>**Fermentation:** It was performed on solid wheat medium (50 g of wheat, 80 mL 0.3% of saline water, in each 1L Erlenmeyer flask) with PDB culture as seed broth. After incubation for 28 days at 25 °C	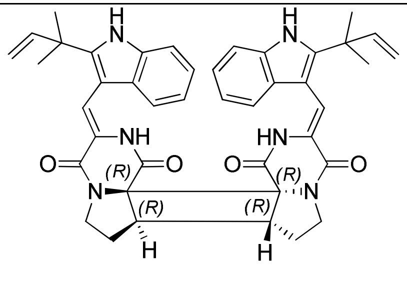 (-)-asperginulin A (**55**) 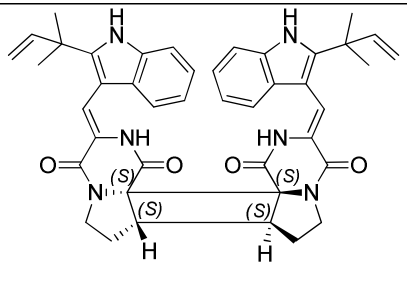 (+)-asperginulin A (**56**)	Compound **55** showed antifouling activity against *Balanus reticulatus*
[5]	**Host plant:** Chinese traditional plant *Hypericum perforatum*<br><br>**Fungus:** *Aspergillus* sp. TJ23<br><br>**Fermentation:** The fungus was grown on rice (rice–water, 1:0.9 m/v) cultural medium (20 kg) for 21 days	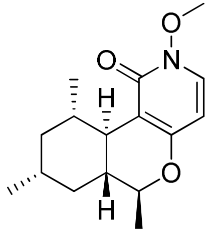 asperpyridone A (**46**)	This compound demonstrated potential use to develop agents for diabetes treatment due to the pronounced glucose uptake effect on liver HepG2 cells under normal and insulin-resistant conditions
[6]	**Host plant:** *Litchi chinensis* Sonn<br><br>**Fungus:** Trichoderma virens FY06<br><br>**Fermentation:** After the initial culture on potato dextrose agar plates, the fermentations were scaled up at 25 °C for 30 days in 75 × 1 L Erlenmeyer flasks statically, each containing autoclaved rice medium (120 mL water, 65 g rice).	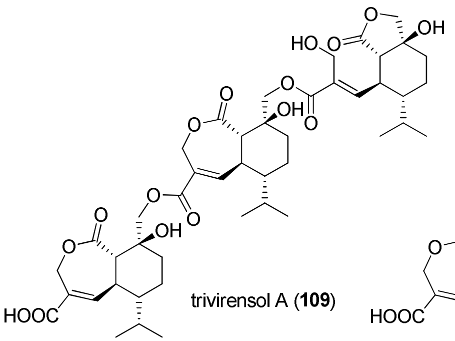 trivirensol A (**109**) 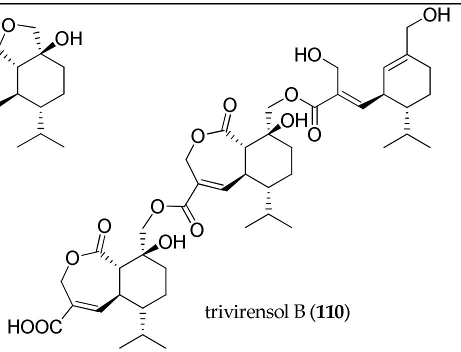 trivirensol B (**110**)	Both compounds showed strong inhibitory activities against phytopathogenic fungi such as *Penicillium italicum*, *Fusarium oxysporum*, *Fusarium graminearun*, *Colletotrichum musae*, and *Colletotrichum gloeosporioides*

[7]	**Host plant:** Chinese medicinal plant *Phyllanthus glaucus*<br><br>**Fungus:** *Aspergillus micronesiensis*<br><br>**Fermentation:** The strain was cultured on potato dextrose agar (PDA) for 5 days to prepare the seed culture. Agar plugs were inoculated into 150 sterilized Erlenmeyer flasks (1 L), each containing 200 g of rice and 200 mL of water. All flasks were incubated at 25 °C for 5 weeks.	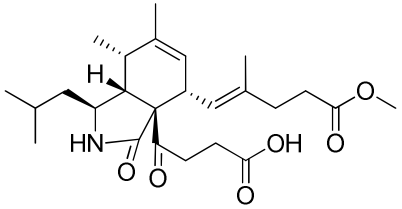 secochalasin A (**1**) 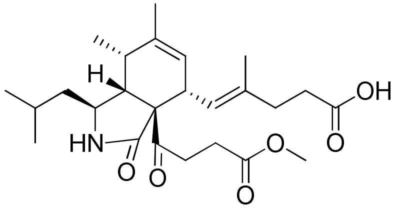 secochalasin B (**2**) 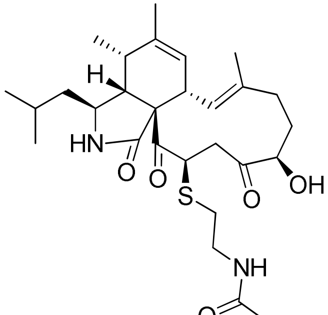 cyschalasin A (**7**) 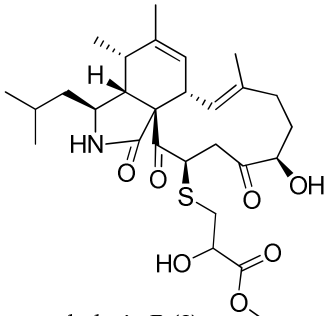 cyschalasin B (**8**)	Compounds **7** and **8** showed antimicrobial activity against Gram-positive bacteria and fungi (*Staphylococcus aureus*, MRSA, and *Candida albicans*), and moderate cytotoxicity against cancer cells (HL-60, A-549, Hep3B, MCF-7, and SW-480), while **1** and **2** were inactive
[8]	**Host plant:** *Solanum tuberosum*<br><br>**Fungus:** *Xylaria* cf. curta<br><br>**Fermentation:** This isolate was cultured in PDB for 7 days and then incubated in solid rice medium for 30 days (100 g rice, 100 mL tap water, 0.1 g $CaCl_2$, 0.1 g $FeSO_4 \cdot 7H_2O$, in 500 mL Erlenmeyer flask.	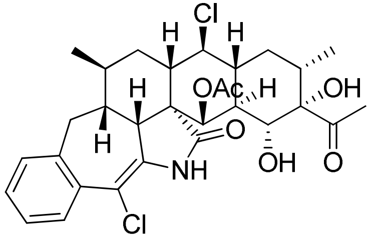 xylarichalasin A (**17**)	Compound **17** showed cytotoxicity against SMMC-7721 and MCF-7 cell lines better than that of the drug cisplatin
[9]	**Host plant:** stem tissues of potato (*Solanum tuberosum*)<br><br>**Fungus:** *Xylaria* cf. curta<br><br>**Fermentation:** Large scale fermentation of X. curta was performed in a 100 L fermentation tank with liquid medium (glucose 50 g, peptone 10 g, yeast extract 10 g, $FeSO_4$ 0.5 g, in 1 L tap water) for 20 days.	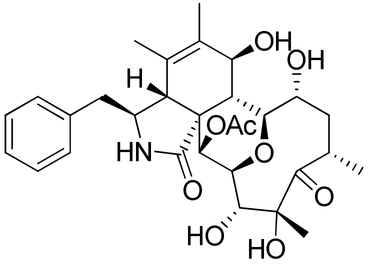 cytochalasin C1 (**5**) 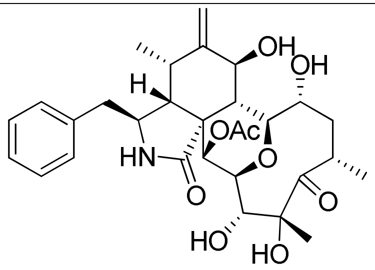 cytochalasin D1 (**6**)	These two compounds showed moderate cytotoxicity against the HL-60 cell line

[10]	**Host plant:** root of the grass *Axonopus leptostachyus*<br><br>**Fungus:** *Aspergillus terreus* P63<br><br>**Fermentation:** The endophytic fungus strain *A. terreus* P63 was cultivated in two 500 mL Erlenmeyer flasks, each containing 30 mL of distilled water and 25.0 g of rolled oats. The media were autoclaved two times (in two consecutive days) at 121 °C for 20 min. Following sterilization, approximately 4 small pieces (2 x 2 cm) of PDA medium, from the Petri dish containing biomass of the *A. terreus* P63 isolated was inoculated into each flask and the flasks were sealed with cotton to permit aerobic growth. The media inoculated with the endophyte were incubated while stationary at 25 °C for 30 days in the dark.	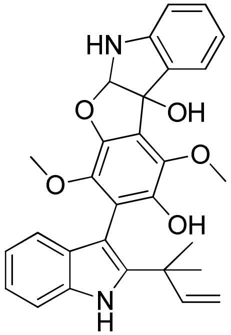<br>giluterrin (**24**)	It presented antiproliferative profile for prostate (PC-3) and kidney (786-0) cancer cell lines
[11]	**Host plant:** tissue of the marine green alga *Codium fragile*<br><br>**Fungus:** *Acrostalagmus luteoalbus* TK-43<br><br>**Fermentation:** the fresh mycelia of *A. luteoalbus* TK-43 were initially grown on PDA medium at 28 °C for 4 days and then were used as seed culture to be inoculated for 40 days at room temperature in 1 L conical flasks with solid rice medium (each flask contained 70 g rice, 0.2 g corn flour, 0.3 g peptone, 0.5 g yeast extract, 0.1 g sodium glutamate, 0.1 g methionine, 70 mg magnesium sulfate heptahydrate, 2 mg ferrous sulfate heptahydrate, and 100 mL naturally sourced and filtered seawater, which was obtained from the Huiquan Gulf of the Yellow Sea near the campus of IOCAS, pH 6.5–7.0).	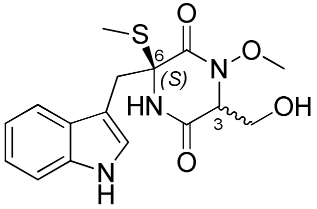<br>(+)-acrozine A 3*R*, 6*S* (**52**)<br>(+)-acrozine B 3*S*, 6*S* (**53**)<br>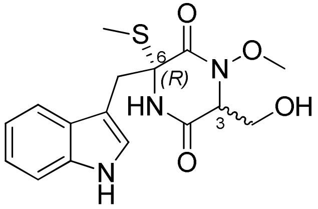<br>(-)-acrozine A 3*S*, 6*R* (**52**)<br>(-)-acrozine B 3*R*, 6*R* (**53**)<br>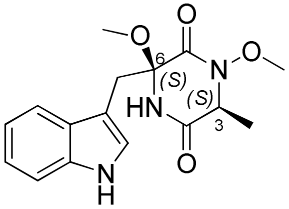<br>(+)-acrozine C 3*S*, 6*S* (**54**)<br>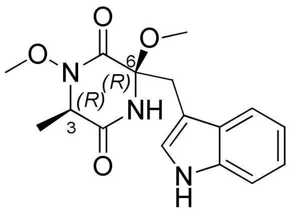<br>(-)-acrozine C 3*R*, 6*R* (**54**)	Pair compounds (+)–**53**/(–)–**53**, showed antifungal activity, and (+)–**52** displayed better inhibitory activity against acetylcholinesterase than (–)–**52** did.

[12]	**Host plant:** Medicinal plant *Tripterygium wilfordii* Hook. f. (*Celastraceae*)<br><br>**Fungus:** *Aspergillus terreus* Thom<br><br>**Fermentation:** The fungal strain was fermented on a potato dextrose agar (PDA) medium at 28 °C for 5 days to perform the seed cultures. Then, the PDA medium was cut into small pieces (approximately 0.3 cm × 0.3 cm × 0.3 cm) and inoculated into 200 × 1000 mL sterilized Erlenmeyer flasks containing 300 mL distilled water and 300 g rice, and cultured at 28 °C for 30 days.	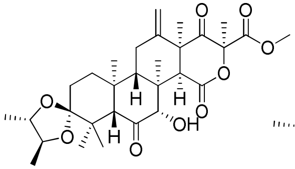 spiroterreusnoid A (**118**) 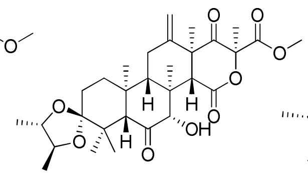 spiroterreusnoid B (**119**) 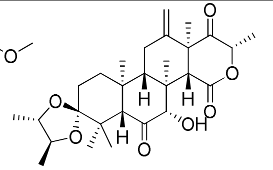 spiroterreusnoid C (**120**)<br>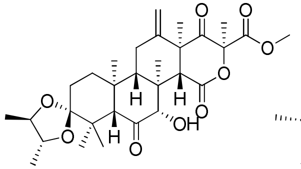 spiroterreusnoid D (**121**) 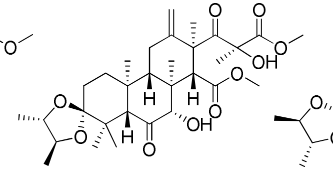 spiroterreusnoid E (**122**) 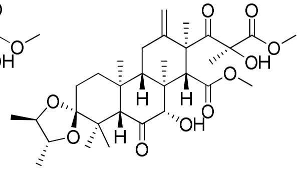 spiroterreusnoid F (**123**)	These compounds exhibited potential abilities in inhibiting BACE1 and acetylcholinesterase (AchE) that might provide a new template for the develpoment of new anti-Alzheimer´s disease drugs.
[13]	**Host plant:** stems of *Rubia podantha*<br><br>**Fungus:** *Colletotrichum* sp. S8<br><br>**Fermentation:** The fungal strain was cultured on potato dextrose agar (PDA) medium at 28 °C. After 4 days, the agar plugs were cut into small fragments in aseptic conditions, inoculated to 28 Erlenmeyer flasks containing rice medium (220 mL deionized water and 180 g rice, sterilized in 121 °C for 30 min), and then fermented at 28 °C for 35 days.	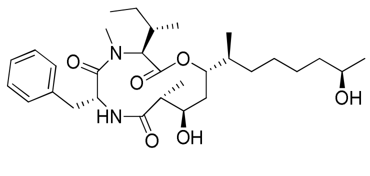 colletopeptide A (**91**) 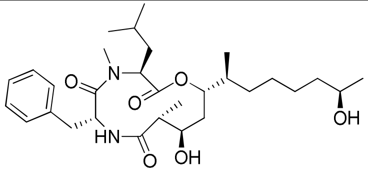 colletopeptide B (**92**)<br>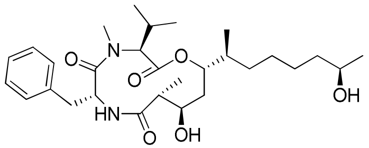 colletopeptide C (**93**) 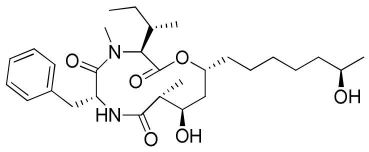 colletopeptide D (**94**)	These compounds exhibited anti-inflammatory activity by inhibiting the production of nitric oxide in RAW264.7 macrophages induced by lipopolysaccharide (LPS); compound **91** also inhibited the production of inflammatory factors IL-6 and TNF- and decreased the phosphorylation of NF- B-associated proteins I B and p65.
[14]	**Host plant:** leaves of the Thai *Xylocarpus granatum* (mangrove)<br><br>**Fungus:** *Phomopsis* sp. xy21<br><br>**Fermentation:** Agar plug (5 mm i.d.) of the fungal strain was inoculated into flasks (500 mL) containing 200 mL of potato dextrose broth (PDB; 20 g glucose and 3 g sea salt in 1 L of potato infusion). Flasks with the inoculated fungal strain were incubated on a rotary shaker (150 rpm) at 28 °C for two days to prepare the seed culture. Large fermentation was performed on solid rice medium in 200 Erlenmeyer flasks (50 g rice, 0.225 g sea salt, and 75 mL distilled water per flask, autoclaved at 121 °C for 25 min before inoculation). Each flask was inoculated with 1.0 mL of the spore inoculum and incubated at 28 °C for 25 days under static conditions.	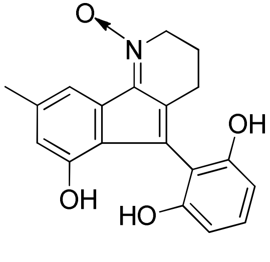 phomopsol A (**90**) 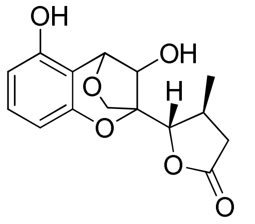 phomopsol B (**184**)	The compound **90** showed neuroprotective effects against corticosterone-induce injury in PC12 cells. Compound **184** did not show a neuroprotective effect.

[15]	**Host plant:** medicinal plant *Mahonia fortune*<br><br>**Fungus:** *Chaetomium nigricolor* F5<br><br>**Fermentation:** This strain was cultured on potato dextrose agar (PDA) medium at 28 °C for 2 weeks. Then, agar plugs (0.5 × 0.5 cm) were inoculated in 20 flasks (500 mL) each containing 80 g of rice, 120 mL of water, and 0.3% peptone. The cultures were further incubated at 28 °C for two months.	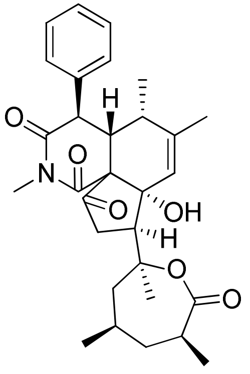<br>chamiside A (**18**)	It exhibited antibacterial activity against *S. aureus*
[16]	**Host plant:** desert plant *Artemisia desertorum*<br><br>**Fungus:** *Trematosphaeria terricola*<br><br>**Fermentation:** The fungal strain was cultured on slants of potato dextrose agar (PDA) at 25 °C for 10 days. The agar plugs were used to inoculate 250 mL Erlenmeyer flasks, each containing 40 mL of media (0.4% glucose, 1% malt extract, and 0.4% yeast extract), and the final pH of the media was adjusted to 6.5 before sterilization. Flask cultures were incubated at 25 °C on a rotary shaker at 170 rpm for five days. Fermentation was carried out in Fernbach flasks (500 mL) each containing 80 g of rice. Spore inoculum was prepared by suspension in sterile, distilled H_2O to give a final spore/cell suspension of 1 × 10^6/mL. Distilled H_2O (100 mL) was added to each flask, and the contents were soaked overnight before autoclaving at 15 lb/in^2 for 30 min. After cooling to room temperature, each flask was inoculated with 5.0 mL of seed culture and cultivated at 25 °C for 40 days.	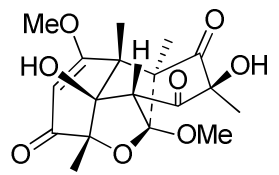<br>trematosphone A (**173**)<br>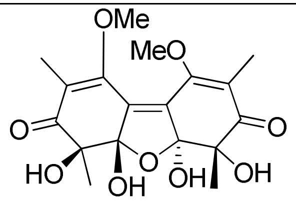<br>trematosphone B (**174**)	Compound **173** showed protective activity against corticosterone-induced damage in PC12 cells. While **174** displayed no activity in the same assay
[17]	**Host plant:** stem of medicinal plant *Isodon sculponeatus*<br><br>**Fungus:** *Alternaria* sp. hh930<br><br>**Fermentation:** The fungal strain was cultured on slants of potato dextrose agar at 28 °C for 7 days. Agar plugs were cut into small pieces (about 0.5 × 0.5 × 0.5 cm^3) under aseptic conditions, and 10 pieces were used to inoculate three Erlenmeyer flasks (250 mL), each containing 50 mL of media (0.4% glucose, 1% malt extract, and 0.4% yeast extract); the final pH of the media was adjusted to 7.0, and the flasks were sterilized by autoclave. Three flasks of the inoculated media were incubated at 28 °C on a rotary shaker at 170 rpm for 5 days to prepare the seed culture. Spore inoculum was prepared in sterile, distilled H_2O to give a final spore/cell	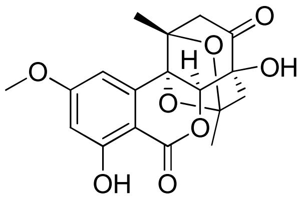<br>(+)-alternarilactone A (**151**)<br>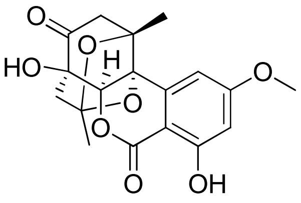<br>(-)-alternarilactone A (**151**)	This pair possessed weak protective activity against corticosterone-induced apoptosis in PC12 cells, and did not show significant antibacterial or antifungal effects against some Gram-positive or -negative pathogens

	suspension of 1 × 10^6/mL. Fermentation was carried out on solid rice medium in 30 Fernbach flasks (500mL, 90mL distilled water was added to 80g rice and kept overnight before autoclaving). Each flask was inoculated with 5.0 mL of the spore inoculum and incubated for 40 days at 28 °C in a static incubator.		
[18]	**Host plant:** stems of medicinal plant *Panax notoginseng*<br><br>**Fungus:** *Preussia isomera* XL-1326<br><br>**Fermentation:** The seed culture of *Preussia isomera* XL-1326 was prepared in Malt Extract Broth (MEB) medium (sucrose 2%, malt extract 2%, peptone 0.1%) and incubated at 28 °C, 160 rpm for 7 days. Large-scale solid fermentation was performed in 100 Erlenmeyer flasks (500 mL), each containing 60 g of rice and 60 mL of MEB medium, as well as 10 mL seed culture. Each flask was cultivated at 28 °C for 48 days at room temperature.	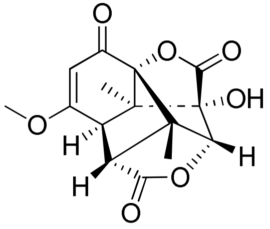 (+)-preuisolactone A (**104**) 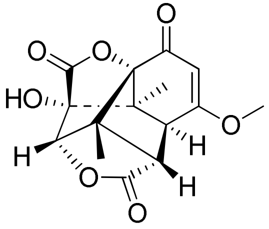 (-)-preuisolactone A (**105**)	Compounds **104** and **105** showed antibacterial activity against *Micrococcus luteus*
[19]	**Host plant:** medicinal plant *Morinda officinalis*<br><br>**Fungus:** *Cytospora rhizophorae* A761<br><br>**Fermentation:** The fungus was cultured in potato dextrose broth (potato 20%, glucose 2%, K_2HPO_4 0.3%, $MgSO_4{\cdot}7H_2O$ 0.15%, vitamin B 10 mg/L). The fungus C. rhizophorae A761 was maintained on potato dextrose agar (PDA) medium at 28 °C for 5 days, and then three pieces (0.5 × 0.5 cm2) of mycelial agar plugs were inoculated into 20 × 500 mL Erlenmeyer flasks, each containing 250 mL potato dextrose broth. After 4 days of incubation at 28 °C on a rotary shaker at 120 rpm, 25 mL seed cultures was aseptically transferred into the 1000 mL flask (a total of 150 flasks), which was contained 500 mL of potato dextrose broth. The liquid cultivation that followed was kept for 7 days at 28 °C and 120 r/m on a rotary shaker.	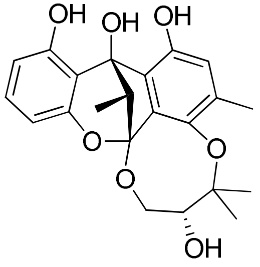 cytorhizin A (**175**) 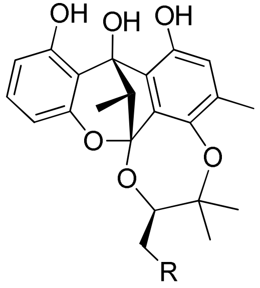 cytorhizin B (**176**): R = Cl<br>cytorhizin C (**177**): R = OMe<br>cytorhizin D (**178**): R = OAc	The compounds were evaluated for cytotoxicity against cancer cell lines, and compounds **176** and **178** showed weak cytoxicity against the HepG-2, MCF-7, SF-268, and A-549 cell lines
[20]	**Host plant:** healthy stem tissues of potato (*Solanum tuberosum*)<br><br>**Fungus:** *Xylaria* cf. curta<br><br>**Fermentation:** The fungus was cultured in PDB for 7 days and then incubated in 100 L broth (50 g glucose, 10 g peptone, 5 g yeast extract, 0.5 g KH_2PO_4, 0.5 g $MgSO_4$, in 1 L) in a fermentation tank to ferment for 30 days.	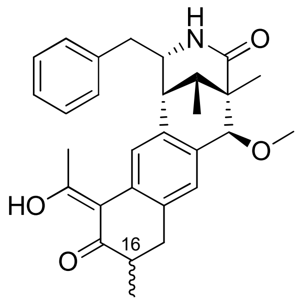 curtachalasin C: 16*S* (**21**)<br>curtachalasin D: 16*R* (**22**) 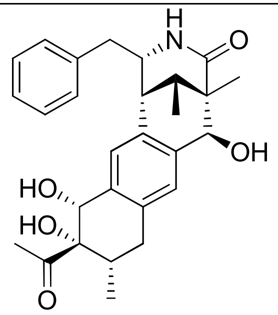 curtachalasin E (**23**)	Compound **21** showed significant resistance reversal activity against fluconazole-resistant *C. albicans*

[21]	**Host plant:** healthy stem tissues of potato (*Solanum tuberosum*)<br><br>**Fungus:** *Xylaria* cf. curta<br><br>**Fermentation:** The fungus was cultured in PDB for 7 days and then incubated in 100 L broth (50 g glucose, 10 g peptone, 5 g yeast extract, 0.5 g KH_2PO_4, 0.5 g $MgSO_4$, in 1 L) in a fermentation tank to ferment for 30 days.	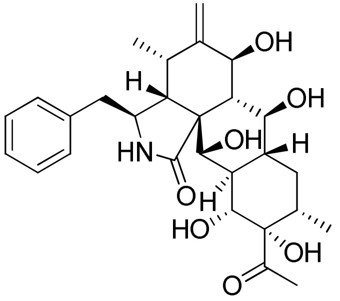 curtachalasin A (**19**) 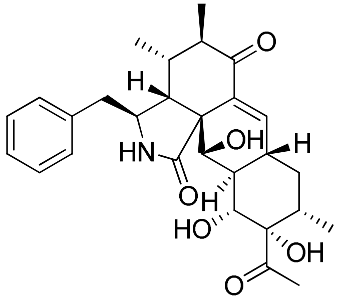 curtachalasin B (**20**)	They showed weak antifungal activity against *Microsporum gypseum*
[22]	**Host plant:** leaves of *Bixa Orellana*<br><br>**Fungus:** *Botryosphaeria mamane* E224<br><br>**Fermentation:** Agar plugs of *B. mamane* E224 were used to inoculate 250 × 250 mL Erlenmeyer flasks, each containing 50 mL of MEA culture medium (prepared with 20 g malt extract, 20 g glucose, 20 g agar, 1 g peptone, 0.005 g $CuSO_4{\cdot}5H_2O$, 0.01 g $ZnSO_4{\cdot}7H_2O$ per L of distilled water). Flask cultures were incubated under static conditions and natural light at 27 °C for 14 days.	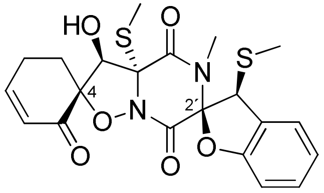 botryosulfuranol A (**65**) 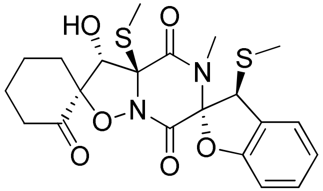 botryosulfuranol B (**66**)	They were evaluated for the cell-growth inhibition of HepG2, HT-2, Caco-2 and HeLa cells. Compound **65** was the most active
[23]	**Host plant:** fresh and healthy potato tissue<br><br>**Fungus:** *Trichothecium crotocinigenum*<br><br>**Fermentation:** This fungal strain was cultured on potato dextrose agar (PDA) medium at 24 °C for 10 days. The agar plugs were inoculated in 500 mL Erlenmeyer flasks, each containing 100 mL of potato dextrose media. Flask cultures were incubated at 27 °C on a rotary shaker at 160 rpm for two days as seed culture. Eighty 500-mL Erlenmeyer flasks each containing 150 mL of potato dextrose broth (PDB) were individually inoculated with 25 mL of seed culture, and were incubated at 27 °C on a rotary shaker at 160 rpm for 25 days.	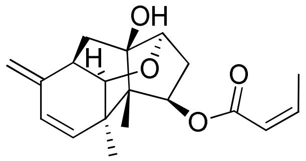 trichothecrotocin A (**111**) 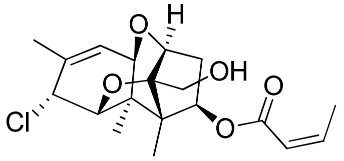 trichothecrotocin B (**112**) 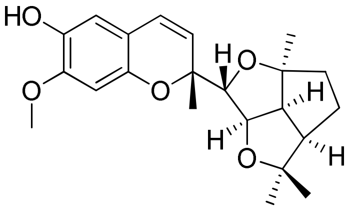 (+)-trichothecrotocin C (**130**) 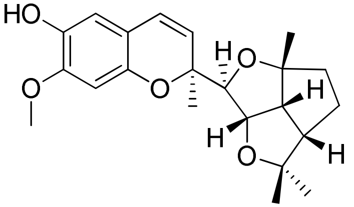 (-)-trichothecrotocin C (**130**)	Compounds **111** and **112** showed potent inhibitory effects against *Alternaria solani* and *Fusarium oxysporum*. And **130** showed potent inhibitory effects against *Alternaria solani*, *Fusarium oxysporum*, and weakly inhibited *Phytophthora infestans* and *Rhizoctonia solani*

[24]	**Host plant:** leaves of medicinal plant *Panax notoginseng*<br><br>**Fungus:** *Emericella* sp. XL 029<br><br>**Fermentation:** The fungus was incubated and cultivated on potato dextrose agar (PDA) at 28 °C for one week. After that, the agar plugs were cut into small pieces and transferred into ten 500 mL flasks consisting of 300 mL PYJ medium (peptone 1%, yeast extract 1%, glucose 1%), and then incubated at 160 rpm, 28 °C for 5 days to get the seed culture. The large scale culture was performed into 150 flasks (500 mL flask), each flask contains 80 g of rice plus 20 mL of malt extract broth (MEB) medium (sucrose 2%, malt extract 2%, peptone 0.1%) and was inoculated with 5 mL the seed culture. After incubation at room temperature for 50 days, fermented cultures from 150 flasks were harvested for chemical study.	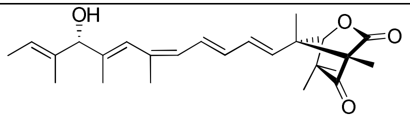<br>emericelactone A (**160**)<br>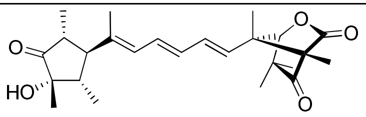<br>emericelactone B (**161**)<br>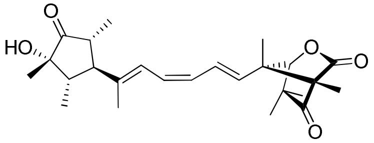<br>emericelactone C (**162**)<br>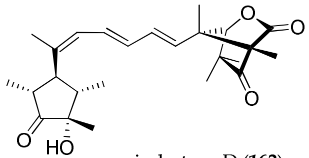<br>emericelactone D (**163**)	All four compounds showed moderate antimicrobial activities against *Verticillium dahliae, R. solani, Gibberella saubinetii, Micrococcus lysodeikticus,* and *Salmonella typhi*
[25]	**Host plant:** leaf tissue of the medicinal plant *Edgeworthia chrysantha*<br><br>**Fungus:** *Penicillium purpurogenum* IMM003<br><br>**Fermentation:** The fungus was inoculated in the solid medium of sterile rice in 30 × 1 L conical flasks (each containing 150 g of rice and 0.2 L of tap water, sterilization at 115 °C/68 kPa for 30 min in an autoclave). After incubation at 28 °C for 40 days.	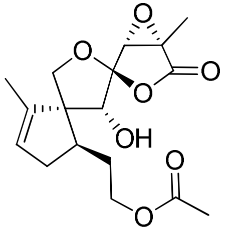<br>purpurolide A (**107**)	It displayed potent inhibition against pancreatic lipase
[26]	**Host plant:** The healthy leaves of *Elaeocarpus decipiens* Hemsl<br><br>**Fungus:** *Aspergillus versicolor*<br><br>**Fermentation:** The fungus was cultured on a solid rice medium (140 g rice + 70 g bran + 200 mL tap water) in ten 3000 mL Erlenmeyer flasks at room temperature for 40 days.	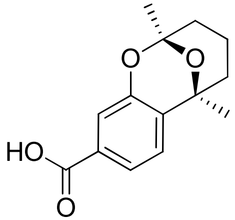<br>aspergoterpenin (**100**)	This compound displayed antimicrobial activity against *Erwinia carotovora* sub sp.
[27]	**Host plant:** marine brown alga *Sargassum* sp.<br><br>**Fungus:** *Trichoderma asperellum* cf44-2<br><br>**Fermentation:** Its fermentation was performed statically at room temperature for 30 days in 200 × 1 L Erlenmeyer flasks, each containing 300 mL of media prepared by addition of 500 mL potato (200 g) broth, 20 g glucose, 5 g peptone, and 5 g yeast extract powder into 500 mL natural seawater from the coast of Yantai	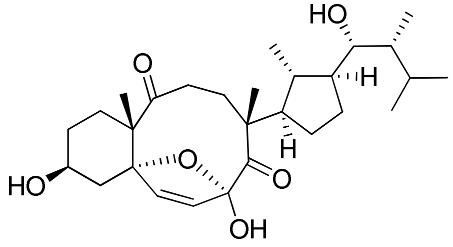<br>tricholumin A (**191**)	It exhibited inhibition against some pathogenic microbes *(V. harveyi, V. splendidus, and Pseudoalteromonas citrea),* marine phytoplankton species *(Chattonella marina, Heterosigma akashiwo, Karlodinium veneficum, Prorocentrum donghaiense),* and antifungal activity against *Glomerella cingulata*

[28]	**Host plant:** fruit of the mangrove plant *Kandelia candel*<br><br>**Fungus:** *Penicillium commune* QQF-3<br><br>**Fermentation:** The fungus was cultured on autoclaved rice medium in 30 Erlenmeyer flasks (1 L), each containing 50 g of rice and 50 mL of 0.3% saline water. After incubation for 28 days at 25 °C, the mycelia and solid rice medium were dried and soaked in EtOAc	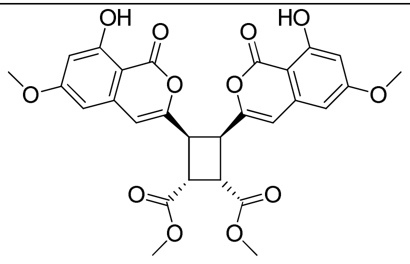<br>peniisocoumarin A (**158**)<br>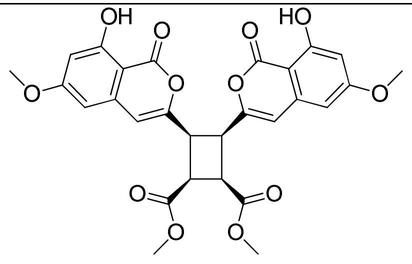<br>peniisocoumarin B (**159**)	These compounds showed an inhibitory effect against α-glucosidase values under 45% at 200 μM
[29]	**Host plant:** the tuber of *Pinellia ternata*<br><br>**Fungus:** *Chaetomium globosum*<br><br>**Fermentation:** After cultivation on PDA medium at 28 °C for 5 days, fresh mycelium was transferred aseptically into a 500mL Erlenmeyer flask containing 150 mL of potato dextrose liquid medium, incubated on a rotary shaker at 28 °C and 150 rpm for 5 days to prepare seed culture. Solid fermentation was carried out in 10 Erlenmeyer flasks (500 mL) each containing 80 g of sterilized rice and 10 mL of seed culture at 28 °C for 30 days	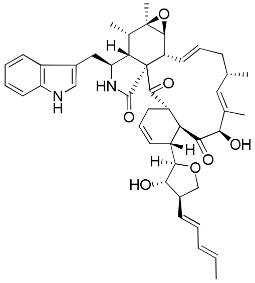<br>aureochaeglobosin A (**11**)<br>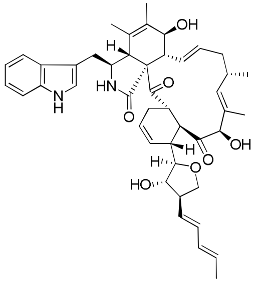<br>aureochaeglobosin B (**12**)<br>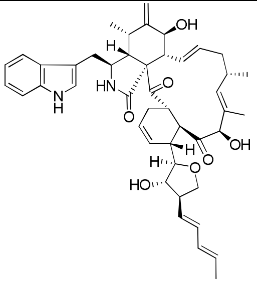<br>aureochaeglobosin C (**13**)	Compounds **12–13** showed significant cytotoxicity against MDA-MB-231 cancer cells
[30]	**Host plant:** leaves of medicinal plant *Phyllanthus glaucus*<br><br>**Fungus:** *Phomopsis* sp.<br><br>**Fermentation:** The fungus was cultured on potato dextrose agar at 28 °C for 5 days as seed cultures before subculturing in 1 L Erlenmeyer flasks with liquid medium (g/L: yeast 3, peptone 5, glucose 10, and maltose 3); the total amount of production medium was 150 L. The cultures were incubated in a rotary shaker incubator at 28 °C at 150 rpm for 10 days.	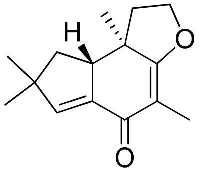<br>phomophyllin A (**108**)	Compound **108** displayed β-site amyloid precursor protein cleaving enzyme 1 (BACE1) inhibitory activity
[31]	**Host plant:** The fresh bark of *Ilex cornuta*<br><br>**Fungus:** *Peyronellaea glomerata*<br><br>**Fermentation:** The fungus was cultivated on PDA for five days, and 32 pieces of the agar were added into eight Erlenmeyer flasks (500 mL) each containing 100 mL of potato dextrose liquid medium. The cultures were incubated using a rotatory shaker at 28 °C and 120 rpm for seven days to prepare the seed culture. Solid fermentation was carried out in 50 Erlenmeyer flasks (500 mL) each containing 100 g of rice and 150 mL of water, which were sterilized at 115 °C for 30 min. After being incubated with 20 mL of seed culture, the flasks were cultivated at 28 °C for 30 days.	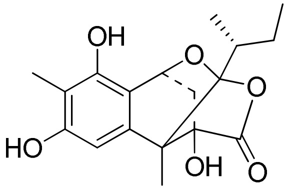<br>peyronellone A (**171**)<br>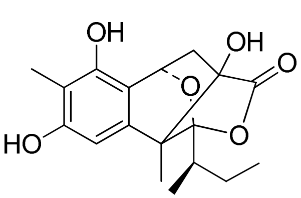<br>peyronellone B (**172**)	Both compounds showed potent antioxidant effects, and compound **172** significantly protects hypoxia/reoxygenation (H/R)-treated human umbilical vein endothelial cells

[32]	**Host plant:** *Cephalotaxus fortunei*<br><br>**Fungus:** *Trichoderma atroviride* S361<br><br>**Fermentation:** After cultured on PDA medium in Petri dish for one week, the fungal mycelium in PDA medium (5 mm in diameter) were punched out and inoculated to 100 Erlenmeyer flasks (1000 mL) using a self-designed cutter. Each shake flask was filled with rice solid medium (250 g rice and 300 mL distilled water), which was subsequently sterilized at 125 °C for 20 min. All flasks were incubated at 25 °C for 30 days.	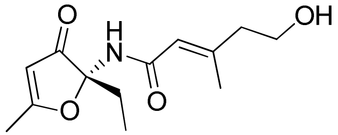<br>(-)-trichodermadione A (**76**)<br>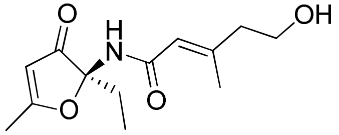<br>(+)-trichodermadione A (**77**)	The two enantiomers did not show activity in anti-inflammatory assay or cytotoxicity test against human prostate cancer cell lines DU-145 and PC-3
[33]	**Host plant:** stem bark of *Sinopodophyllum hexandrum* (Royle) Ying.<br><br>**Fungus:** *Pestalotiopsis adusta*<br><br>**Fermentation:** The strain was grown on a plate of potato dextrose medium consisting of 20 g glucose, 200 g potato, and 20 g agar in 1000 mL distilled water. After incubating at 28 °C for 7 days, a mycelium was inoculated into each of the 100 mL Erlenmeyer flask containing 30 mL of liquid PDB medium consisting of 20 g glucose and 200 g potato in 1000 mL distilled water to obtain the seed strain. The flasks were incubated at 28 °C on a rotary shaker (120 rpm) for 3 days. A suspension (500 μL) of the seed liquid was inoculated aseptically to 500 mL Erlenmeyer flask each containing 40 g rice and 60 mL distilled water, a total of 200 flasks. The flasks were incubated at 28 °C for 30 days.	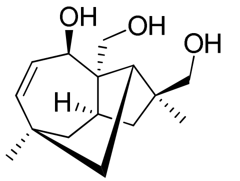<br>pestalustaine A (**106**)<br>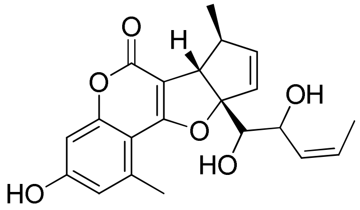<br>pestalustaine B (**155**)	Compounds **106** exhibited weak-to-moderate cytotoxic activity against the HeLa, HCT-116, and A-549 cell lines. And **155** showed weak-to-moderate cytotoxic activity against the HeLa, HCT-116, and A-549 cell lines
[34]	**Host plant:** mangrove *Rhizophora racemosa*<br><br>**Fungus:** *Annulohypoxylon* sp.<br><br>**Fermentation:** The fungus was cultivated on solid rice medium in 10 Erlenmeyer flasks. Solid rice medium was prepared by adding demineralized water (110 mL) to rice (100 g) in an Erlenmeyer flask, followed by autoclaving (121 °C, 20 min). The fungus, which nearly covered the whole surface of a petri dish, was inoculated onto this sterile rice medium under the clean bench and was allowed to grow (20 °C) for 25 days.	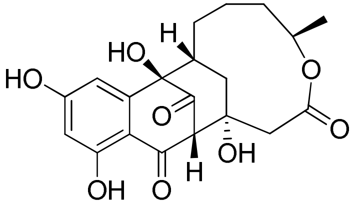<br>hypoxylide (**179**)	Compound **179** did not show cytotoxic and antibacterial activity

[35]	**Host plant:** leaf of the marine mangrove *Kandelia candel*<br><br>**Fungus:** *Ascomycota* sp. SK2YWS-L<br><br>**Fermentation:** The fungus was grown on solid cultured medium in 80 Erlenmeyer flasks (each containing 100g rice and 150 mL 0.3% saline) for 35 days at room temperature under static condition.	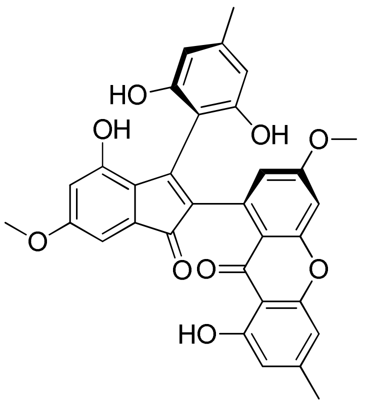 (+)-ascomindone D (**183**) 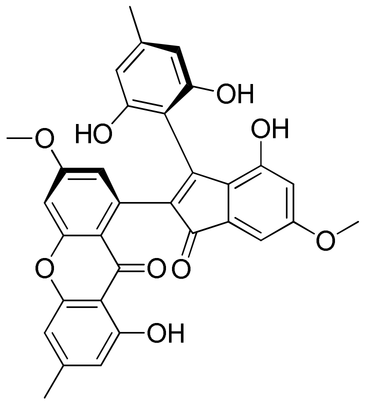 (-)-ascomindone D (**183**)	They showed potential anti-inflammatory effects by inhibiting the production of NO in LPS-activated RAW246.7 cells
[36]	**Host plant:** stem of *Pharbitis nil*<br><br>**Fungus:** *Chaetomium* sp. NF00754<br><br>**Fermentation:** Samples of a five-day fresh mycelium of the fungus grown on the PDA agar plate at 28 °C were inoculated to Erlenmeyer flasks (1000 ml), each containing 400 ml of Martin medium (consisting of 20 g/L sucrose, 0.5 g/L $MgSO_4 \cdot 7H_2O$, 1.0 g/L KH_2PO_4, 1.0 g/L yeast extract, 5.0 g/L peptone and deionized water). After 3 days of incubation at 28 °C with 120 rpm agitation, the seed cultures were used to inoculate 150 flasks each containing 400 ml Martin medium. The fermentation was continued for 14 days at 26 °C with 120 rpm agitation.	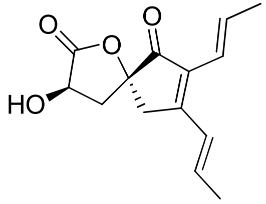 chaetospirolactone (**181**)	Compound **181** was tested in an acetylcholinesterase inhibitory assay but did not show activity
[37]	**Host plant:** *Anvillea garcinii* (Burm.f.) DC. leaves (*Asteraceae*)<br><br>**Fungus:** *Fusarium chlamydosporium*<br><br>**Fermentation:** A sample from preserved fungus was cultivated on solid rice cultures (10 Erlenmeyer flasks 1L). The cultures were incu- bated at room temperature under a septic condition for 30 days.	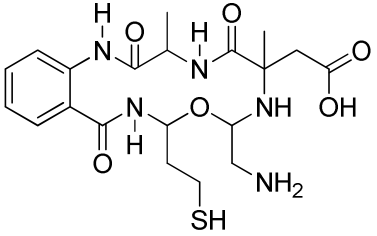 fusarithioamide B (**95**)	It showed selective antifungal activity against *C. albicans*, and moderate activity against *Geotrichum candidum*. Moreover, displayed high antibacterial potential towards *E. coli*, *B. cereus*, and *S. aureus*, a selective and potent effect towards BT-549, MCF-7, SKV-3 and HCT-116, and moderate activity towards the KB and SK-MEL cell lines

[38]	**Host plant:** mangrove plant *Kanelia obovata*<br><br>**Fungus:** *Ascomycota* sp. SK2YWS-L<br><br>**Fermentation:** The fungus was grown in PDA dishes for five days, and then cut into pieces (0.5 cm2) and inoculated in sixty 1 L Erlenmeyer flasks (each containing 100 g rice and 100 mL 0.3% saline water) for 31 days at room temperature under static conditions.	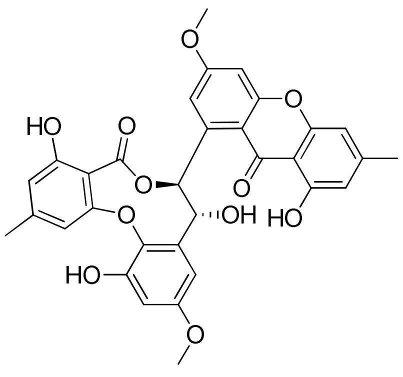<br>(+)-ascomlactone A (**182**)<br>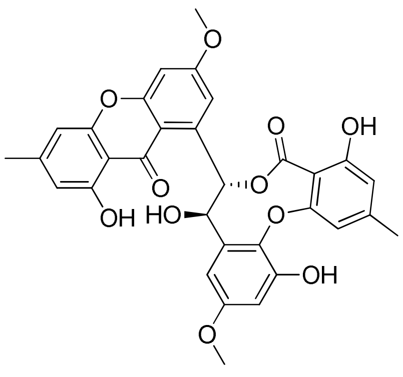<br>(-)-ascomlactone A (**182**)	Both enantiomers showed significant inhibition effects against α-glucosidase
[39]	**Host plant:** medicinal plant *Kadsura longipedunculata*<br><br>**Fungus:** *Aspergillus* sp. CPCC 400735<br><br>**Fermentation:** The fungus was grown on a solid autoclaved rice substrate medium (sixty 500 mL Erlenmeyer flasks, each containing 50 g of rice and 50 mL of 0.3% of saline water) for 28 days at room temperature.	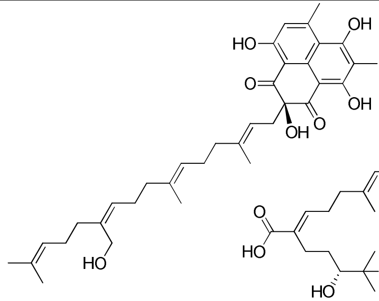<br>asperphenalenone A (**144**)<br>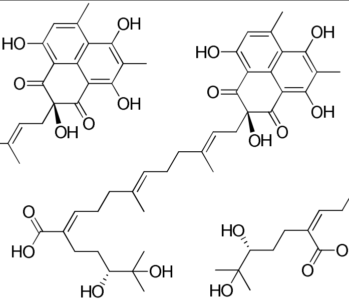<br>asperphenalenone B (**145**)<br>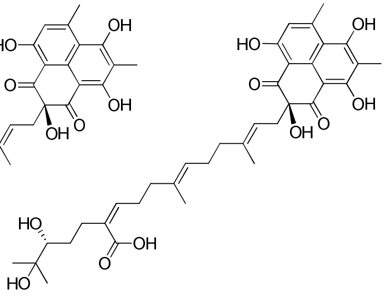<br>asperphenalenone C (**146**)<br>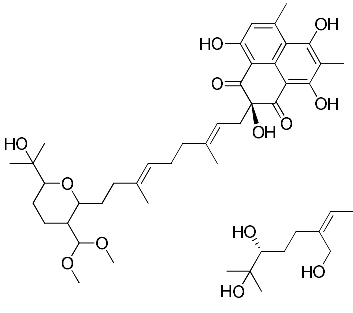<br>asperphenalenone D (**147**)<br>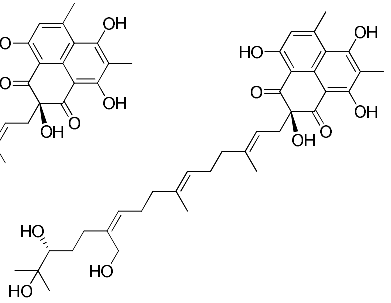<br>asperphenalenone E (**148**)	Compounds **144** and **147** exhibited anti-HIV activity

[40]	**Host plant:** medicinal plant *Myoporum bontioides* A. Gray known as a semi-mangrove plant<br><br>**Fungus:** Penicillium chrysogenum V11<br><br>**Fermentation:** The strain was cultured on PDA (0.3% potato extract, 2% glucose, 1.5% agar power) at 28 °C for 5 days. Under aseptic conditions, agar plugs were cut into small pieces and inoculated in 500 mL Erlenmeyer flasks, each containing 100 mL of seed medium [glucose 2%, sucrose 1%, soybean powder 0.2%, peptone 1%, K_2HPO_4 0.03%, poly(ethylene glycol) 0.25%, $NaNO_3$ 0.3%, and $(NH_4)_2SO_4$ 0.3%; the final pH of the medium was adjusted to 6.0 before sterilization with an autoclave at 121 °C for 20 min]. Cultures were incubated in flasks at 28 °C on a rotary shaker at 190 rpm for 2 days to prepare the seed culture. We filled each of 32 500 mL Erlenmeyer flasks with 80 g of rice and 120 mL of distilled H_2O. After soaking for 5 h, the contents were sterilized with an autoclave at 121 °C for 20 min. After cooling to room temperature, each flask was inoculated with 10 mL of seed culture and incubated at 28 °C for 40 days	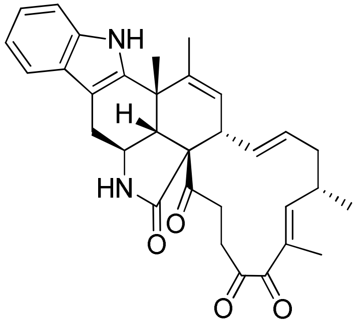<br>penochalasin K (**10**)	It showed cytotoxicity against MDA-MB-435 and SGC-7901 cells, and inhibitory activity against *Rhizoctonia solani* and *Colletotrichum gloeosporioides*
[41]	**Host plant:** mangrove plant *Excoecaria agallocha*<br><br>**Fungus:** *Diaporthe* sp. SYSU-HQ3<br><br>**Fermentation:** The strain was grown on an autoclaved rice medium (100 mL water, 100 g rice, 0.3 g crude sea salt each) in Erlenmeyer flasks (1 L × 100) at 28 °C and grown for 30 days under static conditions.	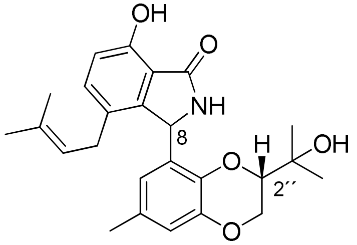<br>diaporisoindole A: 8*S*, 2′′*S* (**39**)<br>diaporisoindole B: 8*R*, 2′′*S* (**40**)<br>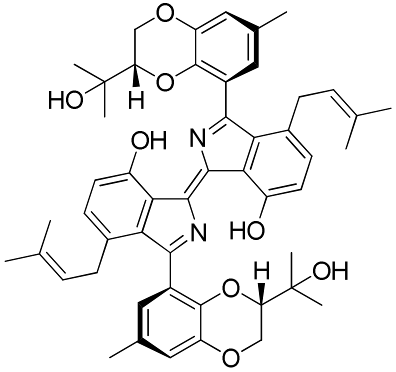<br>diaporisoindole C (**41**)	Compound **41** exhibited inhibitory activity against *Mycobacterium tuberculosis* protein tyrosine phosphatase B
[42]	**Host plant:** root of *Cymbidium goeringii*<br><br>**Fungus:** *Chaetomium* sp.<br><br>**Fermentation:** The fungus was grown on a solid autoclaved rice substrate medium (sixty 500 mL Erlenmeyer flasks, each containing 50 g of rice and 50 mL of 0.3% of saline water) for 28 days at room temperature.	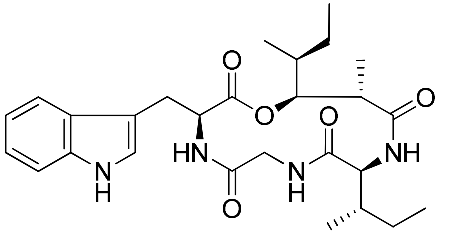<br>chaetomiamide A (**96**)	It did not show cytotoxic activity

[43]	**Host plant:** leaf of the marine mangrove *A. ilicifolius*<br><br>**Fungus:** *Lasiodiplodia theobromae* ZJ-HQ1<br><br>**Fermentation:** After cultivation on potato dextrose agar (PDA) at 28 °C for 4 days, agar plugs were cut into small pieces (about 0.5 × 0.5 × 0.5 cm^3) and shifted to an Erlenmeyer flask (500mL) containing 200 mL potato dextrose broth, which was incubated on a rotary shaker at 180 rpm for 5 days to prepare the seed culture. Fermentation was carried out in ten 500 mL Erlenmeyer flasks containing 80 g rice and 120 mL distilled H_2O. Each flask was inoculated with 20 mL seed liquid after autoclaving, and cultivated under stationary conditions at 28 °C for 40 days.	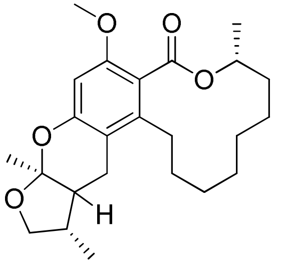 lasiodiplactone A (**180**)	It showed anti-inflammatory activity by inhibiting NO production in LPS-activated RAW264.7 cells
[44]	**Host plant:** *Pinellia ternate* (medicinal plant)<br><br>**Fungus:** *Aspergillus* sp.<br><br>**Fermentation:** After culturing on potato dextrose agar (PDA) at 28 °C for 7 days, sixteen pieces of the agar were transferred into four Erlenmeyer flasks (250 mL) containing 80 mL of potato dextrose liquid medium, which were further incubated at 120 rpm for 6 days to prepare the seed culture. A total of twelve Erlenmeyer flasks (500 mL), each containing 80 g of rice and 120 mL of distilled water were taken for fermentation. All flasks were sterilized at 115 °C for 20 min and incubated with 20 mL of seed culture to ferment at 28 °C for 30 days.	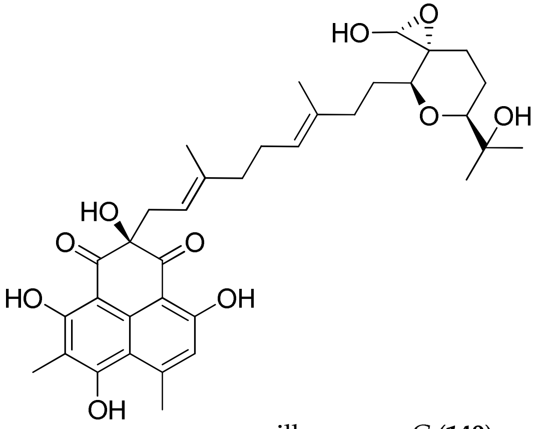 aspergillussanone C (**149**)	Compound **149** did not show antimicrobial activity
[45]	**Host plant:** mangrove plant *Kandelia candel* (L.) Druce (Rhizophoraceae)<br><br>**Fungus:** *Pestalotiopsis vaccinii* (cgmcc3.9199)<br><br>**Fermentation:** The fungus was grown under static conditions at 26 °C for 28 days in one hundred 1000 mL conical flasks containing liquid medium (300 mL/flask) composed of mannitol (20 g/L), maltose (20 g/L), glucose (10 g/L), monosodium glutamate (10 g/L), KH_2PO_4 (0.5 g/L), $MgSO_4 \cdot 7H_2O$ (0.3 g/L), yeast extract (3 g/L), and tap water after adjusting its pH to 7.5. The fermented whole broth (40 L) was filtered through cheesecloth to separate it into filtrate and mycelia	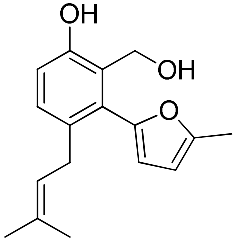 vaccinol J (**143**)	Compound **143** exhibited in vitro antienterovirus 71 activity that was 5.7 times greater than that of assayed positive-control ribavirin

[46]	**Host plant:** Chinese medicinal plant *Panax notoginseng* (BurK.) F.H. Chen.<br><br>**Fungus:** *Trichoderma gamsii*<br><br>**Fermentation:** After culturing on potato dextrose agar (PDA) at 28 °C for 7 days, sixteen pieces of the agar were transferred into four Erlenmeyer flasks (250 mL) containing 80 mL of potato dextrose liquid medium, which were further incubated at 120 rpm for 6 days to prepare the seed culture. A total of twelve Erlenmeyer flasks (500 mL), each containing 80 g of rice and 120 mL of distilled water were taken for fermentation. All flasks were sterilized at 115 °C for 20 min and incubated with 20 mL of seed culture to ferment at 28 °C for 30 days.	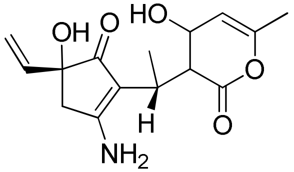<br>trichoderpyrone (**82**)	This compound displayed weak cytotoxicity against the A-549, HepG2, and HeLa cancer cell lines
[47]	**Host plant:** healthy leaves of the marine mangrove *Kandelia obovate*<br><br>**Fungus:** *Talaromyces* sp. (HZ-YX1)<br><br>**Fermentation:** The fungus was cultured on autoclaved rice solid-substrate media (sixty 500 mL Erlenmeyer flasks; each containing 50 g of rice, 1.5 g of artificial sea salts, and 50 mL of distilled H_2O) at room temperature under static conditions and daylight for 28 days.	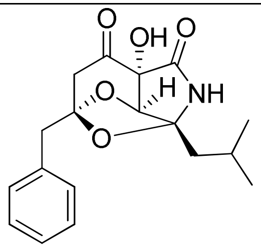<br>talaramide A (**44**)	It displayed promising inhibition of mycobacterial PknG activity
[48]	**Host plant:** Chinese medicinal plant *Xanthium sibiricum*<br><br>**Fungus:** *Eupenicillium* sp. LG41<br><br>**Fermentation:** The fungus was grown under static condition at room temperature for 30 days in 1 L conical flask containing the liquid medium (300 mL/flask) composed of mannitol (20 g/L), sucrose (20 g/L), monosodium glutamate (5 g/L), KH_2PO_4 (0.5 g/L), $MgSO_4$·7H2O (0.3 g/L), yeast extract (3 g/L), corn steep liquor (2 g/L) at pH 6.5.	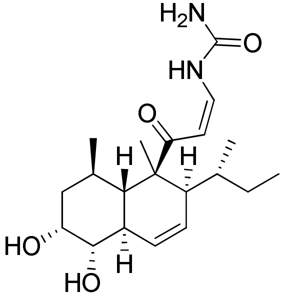<br>eupenicinicol C (**85**)<br>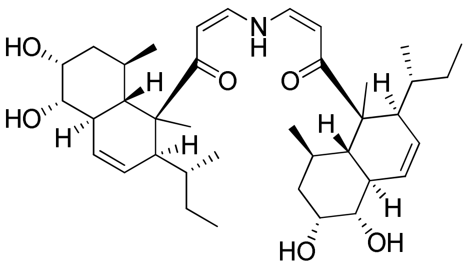<br>eupenicinicol D (**86**)	They displayed antimicrobial activity against *S. aureus* and cytotoxicity against the THP-1 cell line

[49]	**Host plant:** plant *Sumbaviospsis* J. J. Smith<br><br>**Fungus:** *Phoma* sp. YN02-P-3<br><br>**Fermentation:** The fungal strain was cultured on slants of potato dextrose agar at 25 °C for 10 days. Agar plugs were inoculated in 500 mL Erlenmeyer flask containing 120 mL of media (0.4% glucose,1% malt extract, and 0.4% yeast extract; the final pH of the media was adjusted to 6.5) before sterilization, and incubated at 25 °C on a rotary shaker at 170 rpm for one week. Large scale fermentation was carried out in one hundred and fifty 500 mL Fernbach flasks each containing 80 g of rice and 120 mL of distilled H_2O. Each flask was inoculated with 5.0 mL of the culture medium and incubated at 25 °C for 40 days.	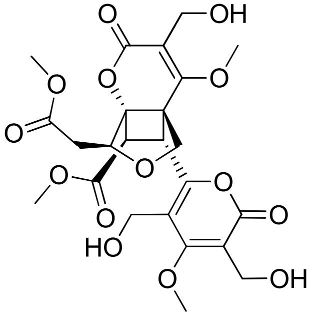 (+)-phomone A (**153**) 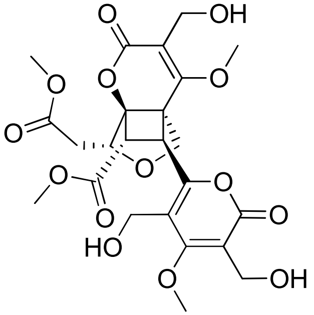 (-)-phomone A (**153**)<br>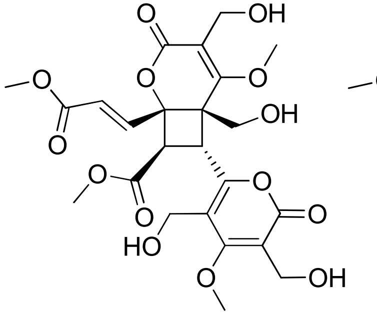 (+)-phomone B (**154**) 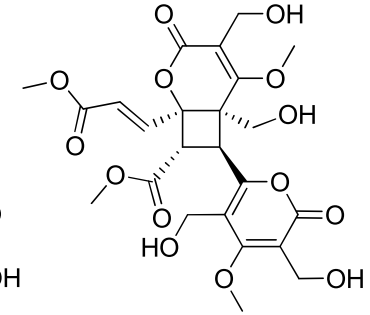 (-)-phomone B (**154**)	They did not show cytotoxicity against three human cancer cells, but the acetylated product of **153** showed moderate cytotoxicity against the HL-60 cell line
[50]	**Host plant:** medically important plant *Phyllanthus glaucus*<br><br>**Fungus:** *Phomopsis* sp. TJ507A<br><br>**Fermentation:** The fungus was cultivated on potato dextrose agar (PDA) at 28 °C for 1 week to prepare the seed cultures. Agar plugs were cut into small pieces (approximately 0.3 × 0.3 cm^2) and inoculated into 150 × 500 mL Erlenmeyer flasks, initially sterilized by autoclaving, each containing 250 g of rice and 250 mL of distilled water.	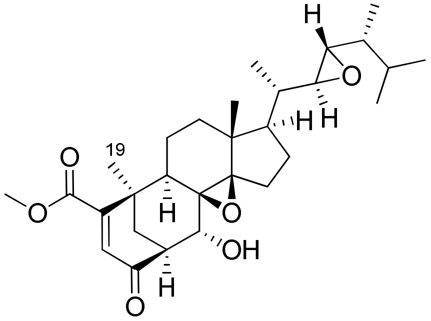<br>phomopsterone A (**190**)	This compound was tested for in vitro anti-inflammatory activity, but did not show considerable activity

[51]	**Host plant:** mangrove root of *Sonneratia caseolaris*<br><br>**Fungus:** *Penicillium janthinellum* HDN13-309<br><br>**Fermentation:** The fungus was cultured under static conditions at 28 °C in 1000 mL Erlenmeyer flasks containing 300 mL of liquid medium containing maltose (2%), mannitol (2%), glucose (1%), sodium glutamate (1%), yeast extract (0.3%), corn syrup (0.1%), KH_2PO_4 (0.05%), and $MgSO_4{\cdot}7H_2O$ (0.03%) dissolved in naturally collected seawater (Huiquan Bay, Yellow Sea, Qiangdao, China).	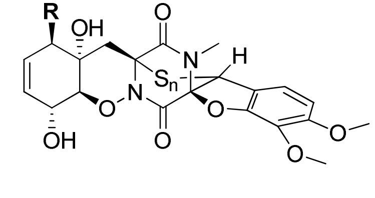 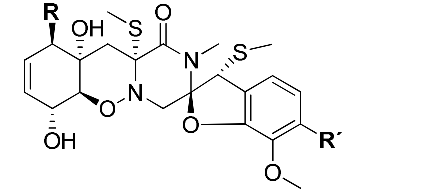<br>penicisulfuranol A: R = Cl, n = 2 (**67**)<br>penicisulfuranol B: R = OH, n = 2 (**68**)<br>penicisulfuranol C: R = OH, n = 3 (**69**)<br>penicisulfuranol D: R = Cl, R′ = OMe (**70**)<br>penicisulfuranol E: R = OH, R′ = OMe (**71**)<br>penicisulfuranol F: R = OH, R′ = H (**72**)	Compounds **67–69** showed cytotoxicity against the HeLa and HL-60 cell lines
[52]	**Host plant:** leaves of *Tapiscia sinensis* Oliv<br><br>**Fungus:** *Penicillium* sp. (NO. 24)<br><br>**Fermentation:** Samples of a five-day fresh mycelium of the fungus grown on the PDA agar plate at 28 °C were inoculated to Erlenmeyer flasks (1000 mL), each containing 400 ml of Martin medium (consisting of 20 g/L sucrose, 0.5 g/L $MgSO_4{\cdot}7H_2O$, 1.0 g/L KH_2PO_4, 1.0 g/L yeast extract, 5.0 g/L peptone and deionized water). After 3 days of incubation at 28 °C with 120 rpm agitation, the seed cultures were used to inoculate 150 flasks each containing 400 ml Martin medium. The fermentation was continued for 14 days at 26 °C with 120 rpm agitation.	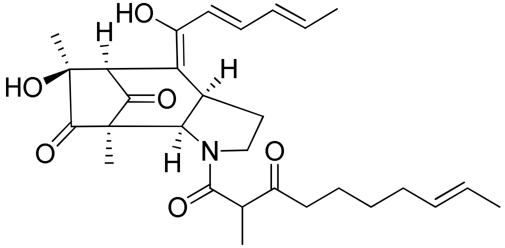<br>penicitroamide (**87**)	It displayed antibacterial activity against *Erwinia carotovora* and *Sclerotium rolfsii*
[53]	**Host plant:** medicinal plant *Hintonia latiflora*<br><br>**Fungus:** *Preussia minimoides*<br><br>**Fermentation:** Erlenmeyer flasks with 50 mL of potato-dextrose broth (Difco) sterilized by autoclaving at 121 °C for 15 min were individually inoculated with 1 cm^3 agar taken from a stock culture of *P. minimoides* on PDA plates. Cultures grown for 1 week were used to inoculate eight 2.8 L Fernbach flasks containing solid rice medium (200 g of white rice and 400 mL of H_2O). *P. minimoides* was cultured at room temperature for 45 days in static conditions with a 12/12 h daily light–dark period.	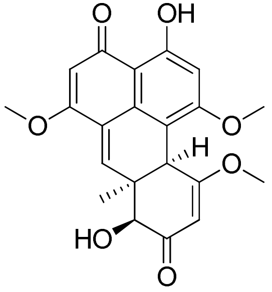 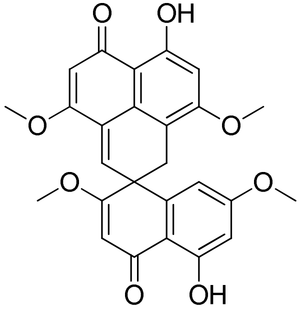<br>minimoidione A (**187**) minimoidione B (**188**)	They inhibited yeast α-glucosidase (αGHY)

[54]	**Host plant:** medicinal plant *Myoporum bontioides*<br><br>**Fungus:** *Penicillium chrysogenum* V11<br><br>**Fermentation:** The fungus was fermented with a rice medium (100 mL water, 100 g rice, 0.3 g crude sea salt) in Erlenmeyer flasks (1 L × 100) at 28 ◦C for 30 days under static conditions.	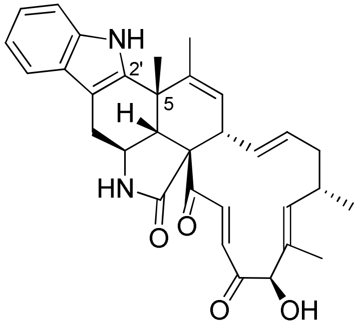<br>penochalasin I (**9**)	It showed cytotoxicity against MDA-MB-435 and SGC-7901 cells, and inhibitory activity against *Rhizoctonia solani* and *Colletotrichum gloeosporioides*
[55]	**Host plant:** mangrove plant *Sonneratia alba*<br><br>**Fungus:** *Cosmospora vilior* IM2-155<br><br>**Fermentation:** This strain was cultivated on sterile steamed unpolished rice with 2% NaCl in water (total 540 g, 20 g/petri dish x 27) at 25° C for three weeks.	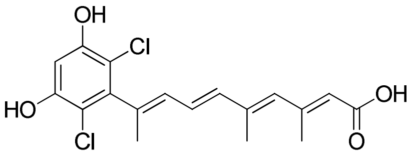<br>cosmochlorin A (**164**)<br>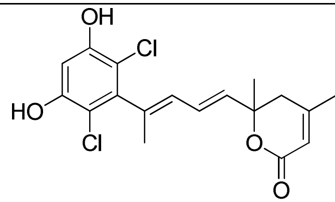<br>cosmochlorin B (**165**)<br>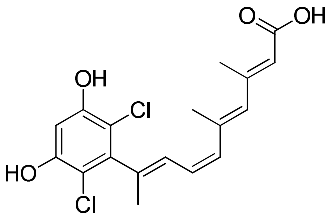<br>cosmochlorin C (**166**)	Compounds **164** and **165** partially restored the growth inhibition caused by hyperactivated Ca^{2+} signaling in mutant yeast, and showed glycogen synthase kinase (GSK)-3β inhibition activity. Additionally, compound **165** increased osteoclast formation in RAW264,7 cells compared to the receptor activator of nuclear factor-κβ ligand (RANKL) alone
[56]	**Host plant:** *Camellia sinensis*<br><br>**Fungus:** *Pestalotiopsis theae* (N635)<br><br>**Fermentation:** The strain was cultured on slants of potato dextrose agar (PDA) at 25 °C for 10 days. Agar plugs were cut into small pieces (about 0.5 × 0.5 × 0.5 cm^3) under aseptic conditions, and 15 pieces were used to inoculate three Erlenmeyer flasks (250 mL), each containing 50 mL of media (0.4% glucose, 1% malt extract, and 0.4% yeast extract); the final pH of the media was adjusted to 6.5 and sterilized by autoclave. Three flasks of the inoculated media were incubated at 25 °C on a rotary shaker at 170 rpm for 5 days to prepare the seed culture. Spore inoculum was prepared by suspension in sterile, distilled H_2O to give a final spore/cell suspension of 1 × 10^6/mL. Fermentation was carried out in 24 Fernbach flasks (500 mL), each containing 80 g of rice.	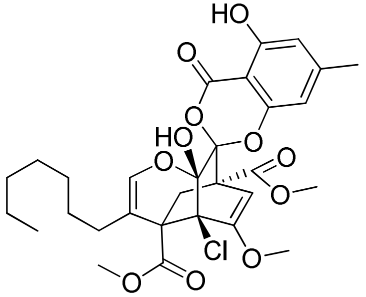<br>chlorotheolide A (**185**)<br>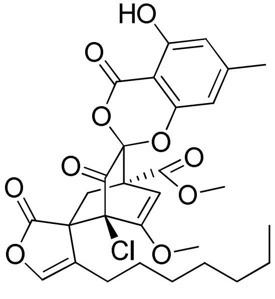<br>chlorotheolide B (**186**)	Compound **186** showed an antiproliferative effect against HeLa and induced an autophagic process in the cells

	Distilled H_2O (120 mL) was added to each flask, and the contents were soaked overnight before autoclaving at 15 psi for 30 min. After cooling to room temperature, each flask was inoculated with 5.0 mL of the spore inoculum and incubated at 25 °C for 40 days. In addition, the strain was cultured on 50 plates of PDA at 25 °C for 7 days.		
[57]	**Host plant:** tissue of the marine mangrove plant *Avicennia marina*<br><br>**Fungus:** *Penicillium brocae* MA-231<br><br>**Fermentation:** The fresh mycelium was inoculated into 1 L flask preloaded with 400 mL of Czapek medium (consisting of 14 g glucose, 9.6 g mannitol, 1.4 g $NaNO_3$, 0.6 g KH_2PO_4, 0.3 g $MgSO_4$, 1.2 g sodium glutamate, 0.6 g sodium tartrate, 6 g sea salt) followed by a four-day culture at 200 rpm/min and 28 o C. The whole liquid (10 × 400 mL) collected from the flask was inoculated onto a 50-L seed tank containing 38 L of sterilized Czapek medium (antifoam 0.5‰, aeration rate 2000NL/h, 150 rpm/min) After a subsequent fermentation at 28 o C for 24 h, the afforded liquid was transferred as seed into a 500 L fermentor preloaded with 300 L of sterilized Czapek medium prior to cultivation at 28 o C for 4 days (antifoam 0.5‰, aeration rate 6000NL/h, 150 rpm/min)	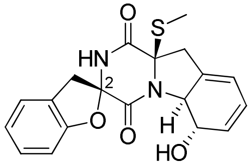 spirobrocazine A (**62**) 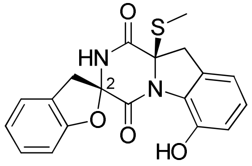 spirobrocazine B (**63**) 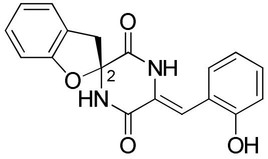 spirobrocazine C (**64**)	Compound **62** exhibited moderate antibacterial activities against *E. coli*, *S. aureus,* and *Vibrio harveyi*; compound **64** showed moderate activity against A-2780 cells, and antibacterial activities against *E. coli*, *Aeromonas hydrophilia,* and *V. harveyi*
[58]	**Host plant:** medicinal plant *Tripterygium wilfordii*<br><br>**Fungus:** *Talaromyces wortmannii* LGT-4<br><br>**Fermentation:** The fungus grown on the potato dextrose agar (PDA) medium (potato 200 g, glucose 20 g, agar 20 g, distilled water 1 L; the media were autoclaved at 121 °C for 20 min) was transferred to twenty 500 mL Erlenmeyer flasks containing 80 g of corn and 100 mL of water (after having been autoclaved) under sterile conditions. Cultures were kept for 45 days at 28 °C under static condition.	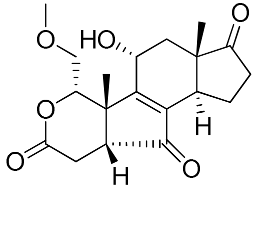 wortmannine A (**134**) 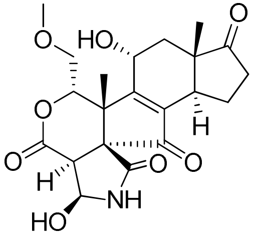 wortmannine B (**135**) 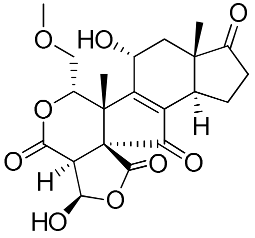 wortmannine C (**136**)	Compounds **134–136** were evaluated for cytotoxicity but showed no activity

[59]	**Host plant:** leaves of medicinal plant *Tamarix chinensis* Lour<br><br>**Fungus:** *Emericella nidulans* HDN12-249<br><br>**Fermentation:** The fungus was cultured under shaking conditions at 28 °C for 9 days in 500 mL Erlenmeyer flasks containing 150 mL liquid culture medium, composed of glucose (1%), maltose (2%), mannitol (2%), monosodium glutamate (1%), KH_2PO_4 (0.05%), $MgSO_4{\cdot}7H_2O$ (0.03%), and yeast extract (0.3%) after adjusting its pH to 6.5.	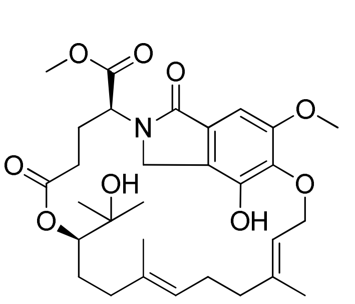<br>emericellolide A (**33**)<br>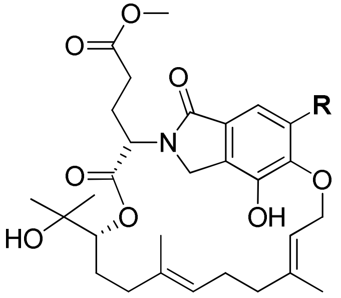<br>emericellolide B: **R** = OCH_3 (**34**)<br>emericellolide C: **R** = OH (**35**)<br>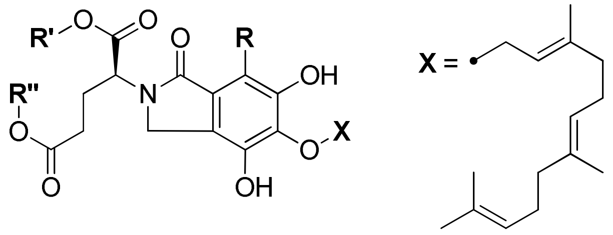<br>emeriphenolicin E: **R** = H, **R'** = X, **R''** = CH_3 (**36**)<br>emeriphenolicin F: **R** = H, **R'** = CH_3, **R''** = X (**37**)<br>emeriphenolicin G: **R** = X, **R'** = CH_3, **R''** = CH_3 (**38**)	Only compound **36** showed cytotoxicity against the HeLa, A-549, and HCT-116 human cancer cell lines
[60]	**Host plant:** root of mangrove plant *Sonneratia caseolaris*<br><br>**Fungus:** *Campylocarpon* sp. HDN13-307<br><br>**Fermentation:** The fungus was cultured under static conditions at 28 °C in 1000 mL Erlenmeyer flasks containing 300 mL liquid medium containing maltose (2%), mannitol (2%), glucose (1%), sodium glutamate (1%), yeast extract (0.3%), corn syrup (0.1%), KH_2PO_4 (0.05%), and $MgSO_4{\cdot}7H_2O$ (0.03%) dissolved in naturally collected seawater (Huiquan Bay, Yellow Sea, Qiangdao, China).	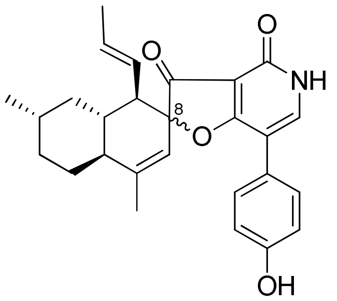<br>campyridone A: 8*R* (**47**)<br>campyridone B: 8*S* (**48**)<br>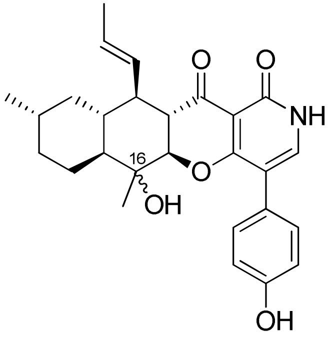<br>campyridone C: 16*R* (**49**)<br>campyridone D: 16*S* (**50**)	Only compound **50** showed cytotoxicity against HeLa cells

[61]	**Host plant:** tissue of the marine mangrove plant *Rhizophora stylosa*<br><br>**Fungus:** *Mucor irregularis* QEN-189<br><br>**Fermentation:** The fungus was grown in a modified Czapek medium (dextrose 10 g, mannitol 20 g, maltose 20 g, yeast extract 3 g, corn syrup 1 g, monosodium glutamate 10 g, tryptophan 0.5 g, K_2HPO_4 0.5 g, $MgSO_4{\cdot}7H_2O$ 0.3 g, 1 L natural seawater, pH = 7.2 - 7.8 adjusted with 10% NaOH or 36.5% HCl) at room temperature under static conditions for 30 days.	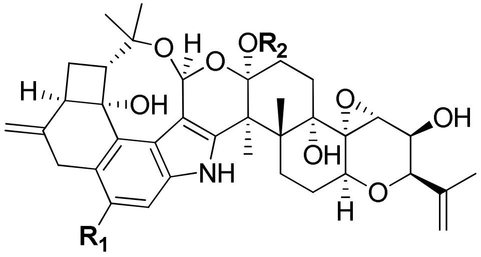<br>rhizovarin A: R_1 = Cl, R_2 = H (**30**)<br>rhizovarin B: R_1 = Cl, R_2 = CH_3 (**31**)<br>rhizovarin C: R_1 = H, R_2 = CH_3 (**32**)	Compounds **30–31** showed activity against the human A-549 and HL-60 cancer cell lines, and **32** showed weak or no activity
[62]	**Host plant:** Baltic Sea algae *Enteromorpha* sp<br><br>**Fungus:** *Coniothyrium cereal*<br><br>**Fermentation:** The fungal strain was cultivated for 40 days on 10 L solid BMS medium (Gesundheitsprodukte GmbH, Kirn, Germany) with agar (15 g/L) at room temperature in 40 Fernbach flasks.	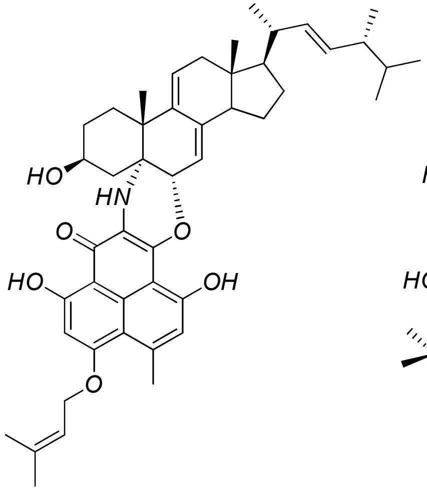 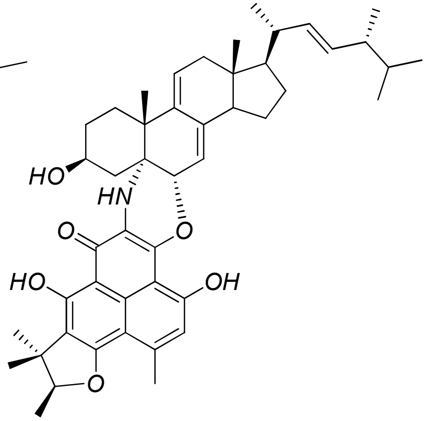<br>conio-azasterol (**83**) *S*-dehydroazasirosterol (**84**)	They did not show antimicrobial and cytotoxic activity
[63]	**Host plant:** *Pritchardia lowreyana*<br><br>**Fungus:** *Peyronellaea coffeae-arabicae* FT238<br><br>**Fermentation:** The fungus was cultured under static conditions at room temperature for 30 days in five conical flask (1 L) containing 300 mL/flask liquid medium. The medium was composed of mannitol (20 g), glucose (10 g), monosodium glutamate (5 g), KH_2PO_4 (0.5 g), $MgSO_4{\cdot}7H_2O$ (0.3 g), yeast extract (3 g), corn steep liquor (2 mL) for 1L distilled water; pH 6.5 prior sterilization.	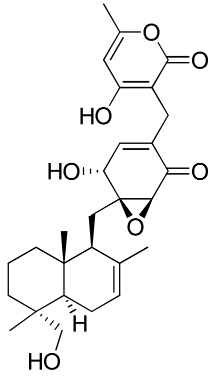 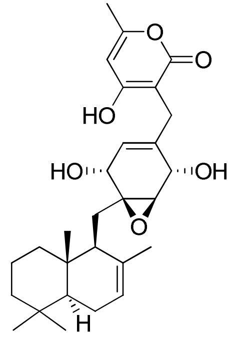 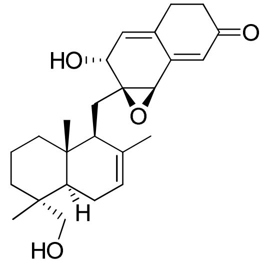<br>peyronellin A (**131**) peyronellin B (**132**) peyronellin C (**133**)	Only compound **131** showed antiproliferative activity against the A-2780 and A-2780 CisR cancer cell lines

[64]	**Host plant:** leaves of the aquatic plant *Eichhornia crassipes*<br><br>**Fungus:** *Aspergillus austroafricanus*<br><br>**Fermentation:** the fungal strain was cultivated axenically on solid rice medium, prepared by autoclaving 100 g of rice and 100 mL of water. Fermentation was carried out for 21 days at 25 °C under static conditions.	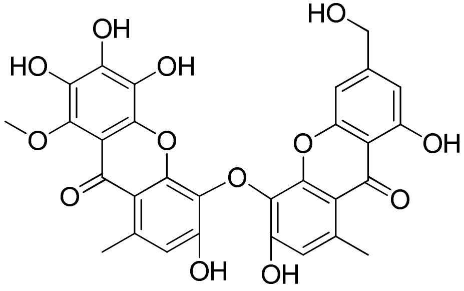<br>austradixanthone (**189**)	It did not show cytotoxicity or antibacterial activity
[65]	**Host plant:** *Rhododendron tomentosum* (medicinal plant)<br><br>**Fungus:** *Penicillium raciborskii*<br><br>**Fermentation:** the endophyte was fermented on malt extract broth medium for 4 to 6 weeks at 23 °C under static conditions in two replicates	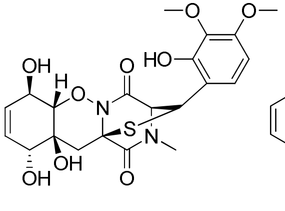<br>outovirin A (**73**)<br>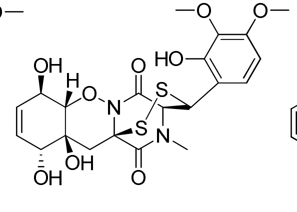<br>outovirin B (**74**)<br>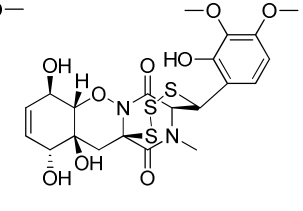<br>outovirin C (**75**)	Compound **75** showed antifungal activity against *Botrytis cinerea* and *Verticillium dahliae*
[66]	**Host plant:** tissue of the marine red alga *Pterocladiella tenuis*<br><br>**Fungus:** *Penicillium chermesinum* EN-480<br><br>**Fermentation:** The fungal strain was grown on PDA medium at 28 °C for 4 days, which was inoculated statically for 30 days in 90 × 1 L Erlenmeyer flasks with rice solid medium at room temperature (each flask contained 70 g of rice, 0.1 g of corn flour, 0.3 g of peptone, 0.1 g of sodium glutamate, and 100 mL of natural, filtered seawater that was obtained from the Huiquan Gulf of the Yellow Sea near the campus of IOCAS, pH 6.5–7.0).	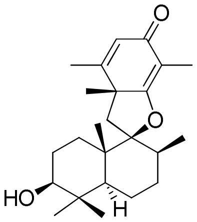<br>chermesin A (**124**)<br>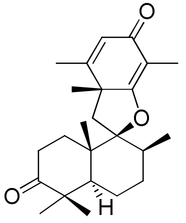<br>chermesin B (**125**)<br>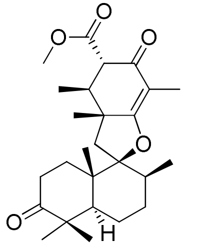<br>chermesin C (**126**)<br>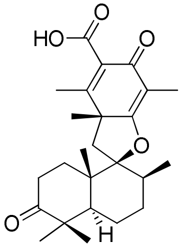<br>chermesin D (**127**)	Compounds **124** and **125** exhibited antibacterial activity against *Micrococcus luteus*
[67]	**Host plant:** medicinal plant *Pogostemon cablin*<br><br>**Fungus:** *Bipolaris sorokiniana* A606<br><br>**Fermentation:** The fungus was grown on potato-dextrose agar (PDA) medium at 28 °C for 5 days and then inoculated into 5 flasks (500 mL) containing potato-dextrose (PD) medium (250 mL). After 5 days of incubation at 28 °C on a rotary shaker at 120 r/m, a portion of the liquid culture was aseptically transferred into each of a total of 240 flasks (1000 mL) containing PDA medium (500 mL). Following 7 days of cultivation at 28 °C and 120 r/m on a rotary shaker, the culture (total of 120 L) was filtered to give the filtrate and mycelia.	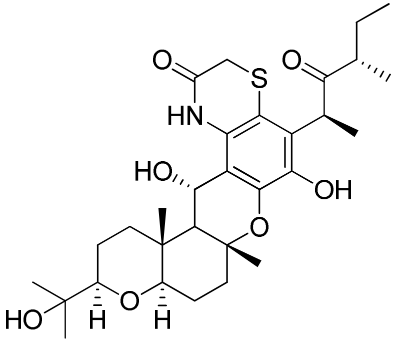<br>isocochlioquinone D (**88**)<br>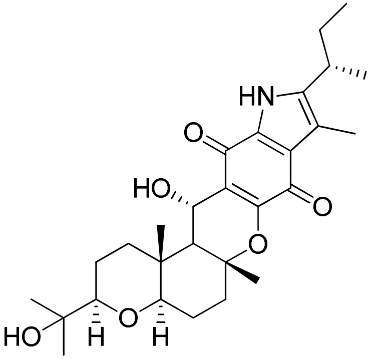<br>cochlioquinone G (**89**)	Compounds **88** and **89** showed cytotoxic effects against the SF-268, MCF-7, NCI-H460 and HepG-2 cell lines

[68]	**Host plant:** medicinal plant *Annona muricata*<br><br>**Fungus:** *Periconia* sp. F-31<br><br>**Fermentation:** The cultivation was performed in Erlenmeyer flasks (1000 mL) containing 300 mL of PDB liquid medium on a shaker at 130 rpm at 25 °C for 10 days.	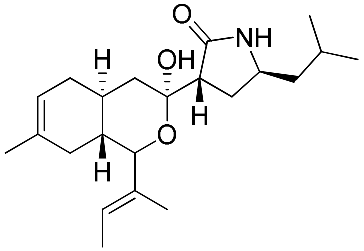<br>pericoannosin B (**45**)	Compound **1** was biologically assayed for cytotoxic (camptothecin as the positive control) and anti-HIV (efavirenz as the positive control) activities, and, it displayed low cytotoxic and anti-inflammatory activities with the IC_{50} values >100 μmol/L.
[69]	**Host plant:** leaves, stems and peels of mangrove plant *X. granatum*<br><br>**Fungus:** *Trichoderma* sp. Xy24<br><br>**Fermentation:** Seed cultures were performed in Erlenmeyer flasks (250 mL) containing 100 mL of PDA liquid medium on a shaker at 150 rpm at 25 °C for 2 days, after that 5 mL seed cultures were inoculated into each 1000 mL flask with 300 mL medium and cultivated for 14 days (150 rpm, 25 °C).	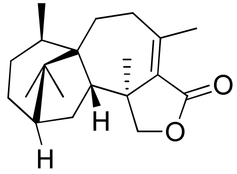<br>harzianelactone (**113**)<br>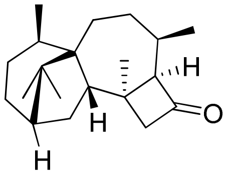<br>(9*R*, 10*R*)-dihydro-harzianone (**114**)	Compound **114** exhibited cytotoxicity against the HeLa and MCF-7 cell lines
[70]	**Host plant:** leaf of the marine mangrove *Kandelia obovata*<br><br>**Fungus:** *Aspergillus terreus* H010<br><br>**Fermentation:** The fungus was grown on liquid cultured medium (composed of maltose (20.0 g/L), mannitol (20.0 g/L), glucose (10.0 g/L), monosodium glutamate (10.0 g/L), $MgSO_4·7H_2O$ (0.3 g/L), KH_2PO_4 (0.5 g/L), yeast extract (3.0 g/L), corn steep liquor (1.0 g/L)) in 80 Erlenmeyer flasks for 45 days at room temperature under static condition.	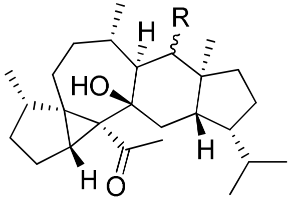<br>aspterpenacid A: R = -$OCOCH_3$, 1*S* (**116**)<br>aspterpenacid B: R = OH, 1*R* (**117**)	Both compounds were tested for antibacterial and cytotoxicity activity, but neither exhibited significant results

[71]	**Host plant:** stems of *Isodon eriocalyx* var. laxiflora. (medicinal plant)<br><br>**Fungus:** *Phomopsis* sp. shj2<br><br>**Fermentation:** The fungal strain was cultured on slants of potato dextrose agar at 25 °C for 7 days. Agar plugs were cut into small pieces (about 0.5 × 0.5 × 0.5 cm^3) under aseptic conditions, and 15 pieces were used to inoculate three Erlenmeyer flasks (250 mL), each containing 50 mL of media (0.4% glucose, 1% malt extract, and 0.4% yeast extract); the final pH of the media was adjusted to 6.5, and the flasks were sterilized by autoclave. Three flasks of the inoculated media were incubated at 25 °C on a rotary shaker at 170 rpm for five days to prepare the seed culture. Fermentation was carried out in 20 Fernbach flasks (500 mL), each containing 80 g of rice. Spore inoculum was prepared in sterile, distilled H2O to give a final spore/cell suspension of 1 × 10^6/mL. Distilled H_2O (120 mL) was added to each flask, and the contents were soaked overnight before autoclaving at 15 psi for 30 min. After cooling to room temperature, each flask was inoculated with 5.0 mL of the spore inoculum and incubated at 28 °C for 40 days.	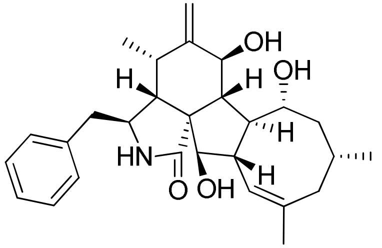<br>phomopchalasin A (**14**)<br>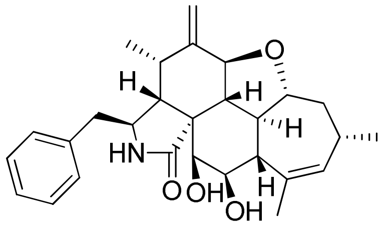<br>phomopchalasin B (**15**)<br>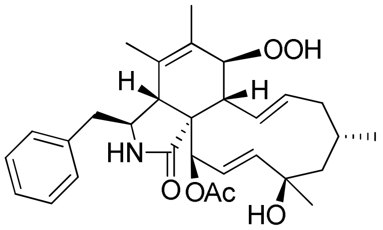<br>phomopchalasin C (**4**)	Compound **4** shows moderate cytotoxicity against H-60, SMMC-7721, and A-549 cell lines, and inhibitory activity against NO production in LPS-activated RAW 264.7 macrophages. And compound **15** exhibited an antimigratory effect against MDA-MB-231 in vitro
[72]	**Host plant:** medicinal plant *Moringa stenopetala*<br><br>**Fungus:** *Mucor irregularis*<br><br>**Fermentation:** It was carried out in two flasks (1 L each). The fungus was grown on rice medium (to 100 g commercially available rice was added 110 mL of distilled water and kept overnight prior to autoclaving), at room temperature under static conditions for 40 days.	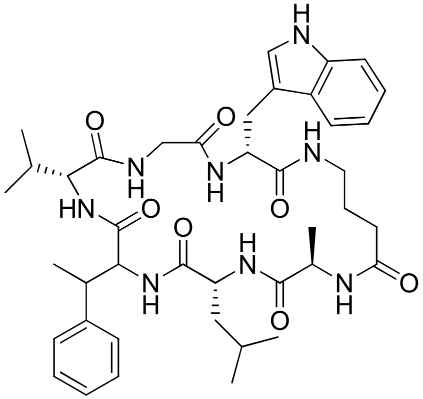<br>unguisin F (**97**)	It did not show biological activity in an antibacterial assay

[73]	**Host plant:** rhizome of medicinal plant *P. polyphylla* var. yunnanensis<br><br>**Fungus:** *Aspergillus oryzae*<br><br>**Fermentation:** The fungus was cultivated at room temperature for 7 days on potato dextrose agar at 28 °C. Agar plugs were inoculated into 250 mL Erlenmeyer flasks each containing 100 mL potato dextrose broth and cultured at 28 °C on a rotary shaker at 180 rpm for 5 days. Large-scale fermentation was carried out in 150 Fernbach flasks (500 mL) each containing 100 g of rice and 120 mL of distilled H_2O. Each flask was inoculated with 5.0 mL of cultured broth and incubated at 25 °C for 30 days.	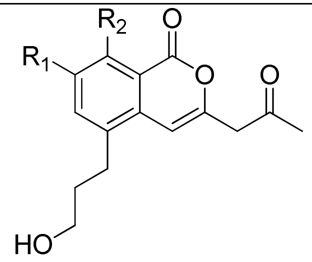<br>oryzaein A: R_1 = OH, R_2 = H (**156**)<br>oryzaein B: R_1 = H, R_2 = OMe (**157**)	Both compounds displayed moderate antitobacco mosaic viral activities
[74]	**Host plant:** mangrove tree, *Bruguiera gymnorrhiza*<br><br>**Fungus:** *Pseudolagarobasidium acaciicola*<br><br>**Fermentation:** The fungus was cultured on Potato Dextrose Agar (PDA) (HiMedia Laboratories, Mumbai, India) containing 10% of seawater (collected from Bang Saen Beach, Chonburi Province) for 1 week. The agar was then inoculated into 500 mL of Potato Dextrose Broth (PDB) (HiMedia Laboratories, Mumbai, India) containing 10% of seawater. After incubation for 21 days, broth (total volume of 500 mL) and fungal mycelia were separated by filtration using filter paper (Whatman, Grade 1).	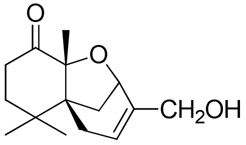<br>acaciicolide A (**101**)<br>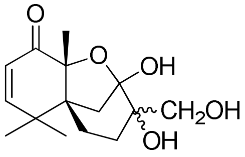<br>acaciicolide B (**102**)<br>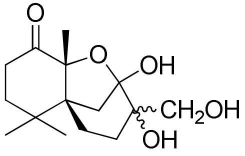<br>acaciicolide C (**103**)	They did not show cytotoxic activity
[75]	**Host plant:** the medicinal plant *Annona muricata*<br><br>**Fungus:** *Periconia* sp<br><br>**Fermentation:** The cultivation was performed in Erlenmeyer flasks (1000 ml) containing 300 ml of PDA liquid medium on a shaker at 130 rpm at 25 °C for 10 days.	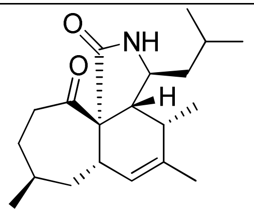<br>periconiasin G (**3**)	It showed weak anti-HIV activity

[76]	**Host plant:** root of the mangrove plant *Aricennia marina*<br><br>**Fungus:** *Neosartorya udagawae* HDN13-313<br><br>**Fermentation:** The fungus was cultured at 28 °C on a rotary platform shaker at 180 rpm for 9 days in 500 mL Erlenmeyer flasks containing 150 mL of liquid culture medium, composed of maltose (20.0 g/L), mannitol (20.0 g/L), glucose (10.0 g/L), monosodium glutamate (10.0 g/L), $MgSO_4{\cdot}7H_2O$ (0.3 g/L), KH_2PO_4 (0.5 g/L), yeast extract (3.0 g/L), corn steep liquor (1.0 g/L), and seawater (Huiquan Bay, Yellow Sea). After cultivation, 40 L of whole broth was filtered through cheesecloth to separate the supernatant from the mycelia.	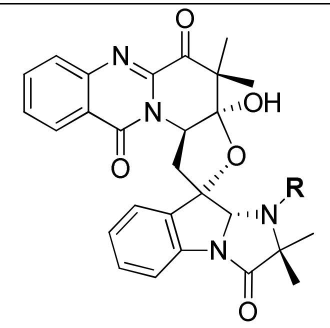<br>neosartoryadin A: R = H (**26**)<br>neosartoryadin B: R = OH (**27**)	Compounds **26** and **27**displaye d anti-influenza virus A (H1N1) activities with IC50 values of 66 and 58 μM, respectively (ribavirin as positive control, IC50 = 94 μM)
[77]	**Host plant:** tissue of the surface-sterilised marine brown alga *D. prolifera*<br><br>**Fungus:** *Trichoderma citrinoviride* cf-27<br><br>**Fermentation:** The fresh mycelia were grown on the potato dextrose agar plates. Then, pieces of mycelia were cut into small segments and aseptically inoculated into 200 Erlenmeyer flasks (1 L), each containing 100 g rice, 0.6 g peptone and 100 mL natural sea water. Static fermentations were performed at room temperature for 30 days.	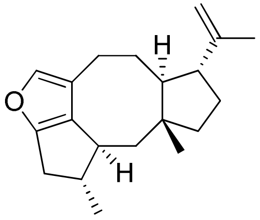<br>trichocitrin (**115**)	It exhibited inhibition against *E. coli*
[78]	**Host plant:** *Euphorbia sieboldiana* (medicinal plant)<br><br>**Fungus:** *Guignardia* sp.<br><br>**Fermentation:** After cultivation on potato dextrose agar (PDA) at 28 °C for 7 days, two pieces of the agar (about 1.0 cm3) were added to an Erlenmeyer flask (500 mL) with 150 mL of potato dextrose liquid medium, and the flask was incubated on a rotary shaker at 28 °C and 150 rpm for 5 days to prepare seed culture. Solid fermentation was carried out in 12 Erlenmeyer flasks (500 mL) each containing 120 g of sterilized rice and 10 mL of seed culture, and the flaks were cultivated at 28 °C for 30 days.	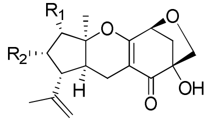<br>13-hydroxylated guignardone A: R_1 = H, R_2 = OH (**137**)<br>12-hydroxylated guignardone A: R_1 = OH, R_2 = H (**138**)<br>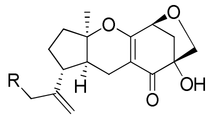<br>17-hydroxylated guignardone A: R = OH (**139**)<br>guignardone M: R = OAc (**140**)<br>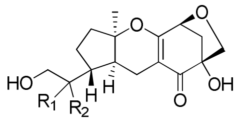<br>guignardone N: R_1 = α-OH, R_2 = β-CH_3 (**141**)<br>guignardone O: R_1 = α-CH3, R_2 = β-OH (**142**)	Compound **137** exhibited moderate antibacterial activity against *Pseudomonas aeruginosa*, while **141** showed prominent inhibition on the growth of *C. albicans*

[79]	**Host plant:** *Crocus sativus*<br><br>**Fungus:** *Phialophora mustea*<br><br>**Fermentation:** A week old culture medium (1%) was used to inoculate the 50 L stirrer tank reactor (Scigenic India Ltd), containing 25 L of potato dextrose broth (HiMedia, India). The fermentor was run with 100 RPM agitation at 25 °C and 15 LPM air for 10 days.	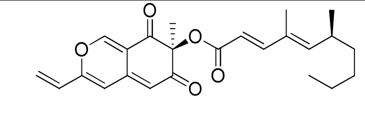<br>phialomustin A (**167**)<br>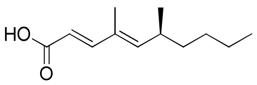<br>phialomustin B (**168**)<br>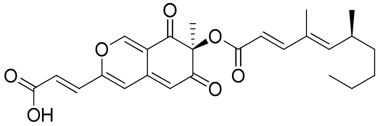<br>phialomustin C (**169**)<br>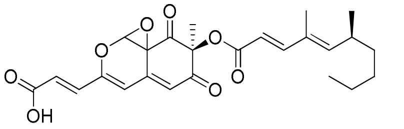<br>phialomustin D (**170**)	Compound **168** exhibited cytotoxic activity against the T47D cell line; compounds **169** and **170** showed promisory antifungal activity against *C. albicans*
[80]	**Host plant:** rhizomes of *Tripterygium wilfordii* (medicinal plant)<br><br>**Fungus:** *Talaromyces wortmanni*<br><br>**Fermentation:** The fungus was grown on potato dextrose agar (PDA) at 28°C for five days. Mycelia were inoculated into 500 mL Erlenmeyer flasks containing 300 mL potato dextrose broth (PDB) at 28°C for five days in a rotary shaker. The culture (2 L) was inoculated into 25 L fermentation tank containing 20 L potato dextrose broth (PDB) at room temperature for three weeks.	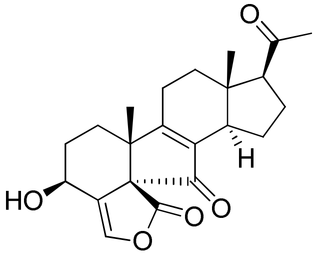<br>secovironolide (**192**)	It showed weak inhibitory activity in a monoamine oxidase assay
[81]	**Host plant:** tissue of the fresh leaves of marine mangrove plant Rhizophora stylosa<br><br>**Fungus:** *Penicillium oxalicum* EN-201<br><br>**Fermentation:** The culture was grown by solid-substrate fermentation on rice (carried out in 1 L Erlenmeyer flasks, each containing 70 g of rice and 100 mL seawater). After incubation for 30 days at room temperature, the whole culture was extracted exhaustively with EtOAc.	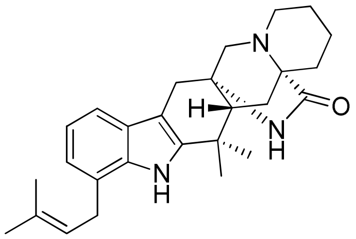<br>penioxamide A (**25**)<br>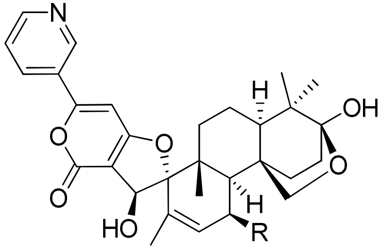<br>18-hydroxydecaturin B: R = OH (**51**)	Compound **25** showed potent brine-shrimp lethality with LD_{50} value of 5.6 μM. And **51** showed brine-shrimp lethality with LD_{50} value of 2.3 μM
[82]	**Host plant:** branches of *Camellia sinensis* (*Theaceae*)<br><br>**Fungus:** *Pestalotiopsis fici*<br><br>**Fermentation:** The fungal strain was cultured on slants of potato dextrose agar (PDA) at 25 °C for 10 days. Agar plugs were cut into small pieces (about 0.5 × 0.5 × 0.5 cm^3) under aseptic conditions and 15 pieces were used to inoculate three Erlenmeyer flasks (250 mL), each containing 50 mL of media (0.4 % glucose, 1 % malt extract, and 0.4 % yeast extract); the final pH of the media was adjusted to 6.5 and sterilized by autoclave. Three flasks of the inoculated media were incubated at 25 °C on a rotary shaker at 170 rpm for five days to prepare the seed culture. Spore inoculum was prepared by	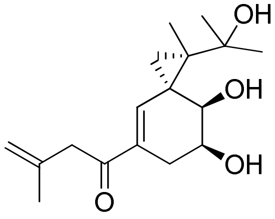<br>pestalotriol A (**128**)<br>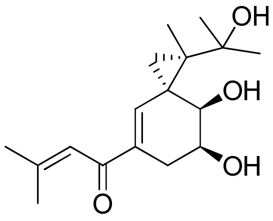<br>pestalotriol B (**129**)	Only compound **129** showed weak cytotoxicity against HeLa cells

	suspension in sterile, distilled H_2O to give a final spore/cell suspension of 1 × 10^6/mL. Fermentation was carried out in 36 Fernbach flasks (500 mL), each containing 80 g of rice. Distilled H_2O (120 mL) was added to each flask, and the contents were soaked overnight before autoclaving at 15 psi for 30 min. After cooling to room temperature, each flask was inoculated with 5.0 mL of the spore inoculum and incubated at 25 °C for 40 days.		
[83]	**Host plant:** branches of *Acer truncatum* Bunge<br><br>**Fungus:** *Paraconiothynium brasiliense* Verkley<br><br>**Fermentation:** The fungal strain was cultured on slants of potato dextrose agar at 25 °C for 10 days. Agar plugs were cut into small pieces (about 0.5 × 0.5 × 0.5 cm^3) under aseptic conditions, 15 pieces were used to inoculate in three Erlenmeyer flasks (250 mL), each containing 50 mL of media (0.4 % glucose, 1% malt extract, and 0.4% yeast extract), and the final pH of the media was adjusted to 6.5. After sterilization, three flasks of the inoculated media were incubated at 25 °C on a rotary shaker at 170 rpm for five days to prepare the seed culture. Spore inoculum was prepared by suspending the seed culture in sterile, distilled H_2O to give a final spore/cell suspension of 1 × 10^6/mL. Fermentation was carried out in 12 Fernbach flasks (500 mL), each containing 80 g of rice. Distilled H_2O (120 mL) was added to each flask, and the contents were soaked overnight before autoclaving at 15 psi for 30 min. After cooling to room temperature, each flask was inoculated with 5.0 mL of the spore inoculum and incubated at 25 °C for 40 days.	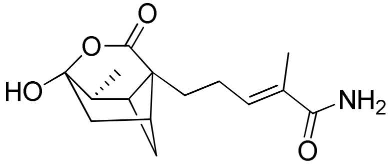<br>brasilamide K (**78**)<br>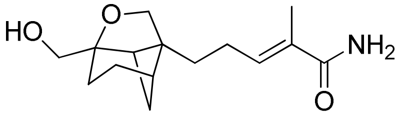<br>brasilamide L (**79**)<br>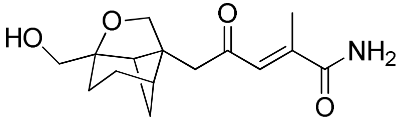<br>brasilamide M (**80**)<br>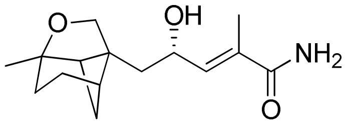<br>brasilamide N (**81**)	These compounds were tested against eight human tumor cell lines, but did not show detectable cytotoxicity
[84]	**Host plant:** a root of a mangrove tree, *Heritiera littoralis*<br><br>**Fungus:** Penicillium chermesinum<br><br>**Fermentation:** The fungus was grown on potato dextrose agar (PDA) for nine days and then cultivated into 1 L Erlenmeyer flasks (40 flasks), each flask containing 250 mL of potato dextrose broth (PDB) and incubated for 31 days at room temperature.	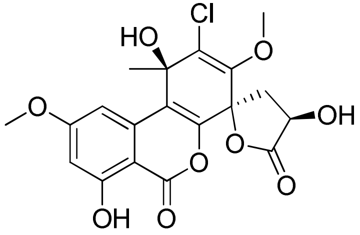<br>penicilliumolide A (**152**)	It did not show cytotoxicity activity

[85]	**Host plant:** leaves of the Chinese medicinal plant *Mahonia fortune*<br><br>**Fungus:** *Diaporthe* sp. LG23<br><br>**Fermentation:** Agar plugs from a week-old culture plate (grown on PDA at 28 ± 2 °C) were cut into small pieces under aseptic conditions, and 40 pieces were used to inoculate 20 flasks (1 L) each containing 80 g of rice, 120 mL of water, and 0.3% peptone. The cultures were incubated at room temperature for two months.	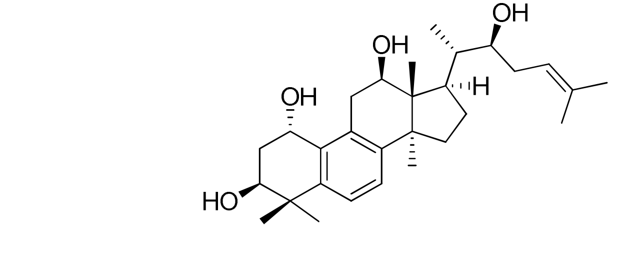<br>19-nor-lanosta-5(10),6,8,24-tetraene-1-alpha,3-beta,12-beta,22S-tetraol (**193**)	Compound **193** showed pronounced antibacterial efficacy against *S. aureus, E. coli, B. subtilis, P. aeruginosa,* and *S. pyogenes*
[86]	**Host plant:** *Lycopodiella cernua* (L.) Pic. Serm (medicinal plant)<br><br>**Fungus:** *Paraphaeosphaeria neglecta* FT462<br><br>**Fermentation:** The fungus was cultured under static conditions at room temperature for 30 days in five conical flask (1 L) containing 300 mL/flask liquid medium. The medium was composed of mannitol (20 g), glucose (10 g), monosodium glutamate (5 g), KH_2PO_4 (0.5 g), $MgSO_4·7H_2O$ (0.3 g), yeast extract (3 g), corn steep liquor (2 mL) for 1L distilled water; pH 6.5 prior sterilization	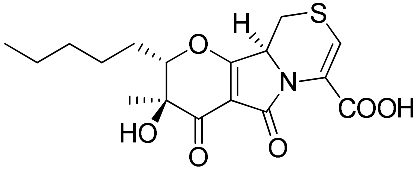<br>paraphaeosphaeride A (**43**)	No activity was reported
[87]	**Host plant:** stem bark of *Melia azedarach* L.<br><br>**Fungus:** *Aspergillus* sp<br><br>**Fermentation:** The fungal strain was cultured in potato-dextrose broth medium (500 mL Erlenmeyer flasks containing 150 mL of medium) at 28 °C with a rotary shaker at 150 rpm for seven days. Then, 10 ml of seed was carried out on solid culture which was consist of 80 g of rice and 125 ml of distilled water.	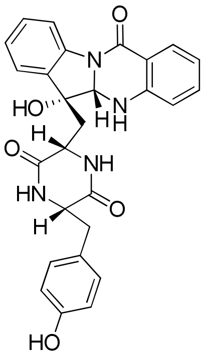<br>aspertryptanthrin A (**57**)<br>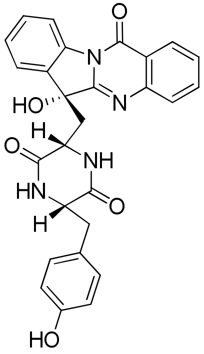<br>aspertryptanthrin B (**58**)<br>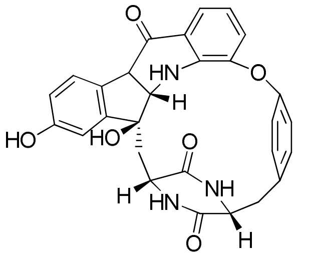<br>aspertryptanthrin C (**59**)	They were evaluated against the U-20S, MCF-7, HepG2, and HeLa cell lines, but did not show activity
[88]	**Host plant:** marine red alga *Polysiphonia scopulorum* var. villum<br><br>**Fungus:** *Aspergillus nidulans* EN-330<br><br>**Fermentation:** The fungus was statically fermented on PDB (2% mannitol, 1% glucose, 0.3% peptone, 0.5% yeast extract) medium in 1 L Erlenmeyer flasks (300 mL/flask, 60 flasks) and incubated for 30 days at room temperature.	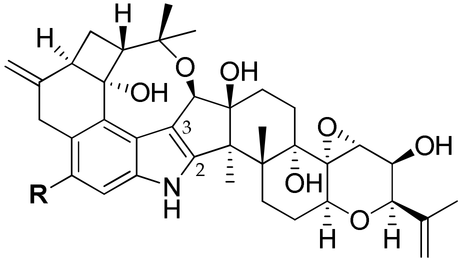<br>19-hydroxypenitrem A: R = Cl (**28**)<br>19-hydroxypenitrem E: R = H (**29**)	They showed cytotoxic activity against brine shrimp, and antimicrobial activity against both human (*E. coli, S. aureus*) and aqua pathogens (*Edwardsiella tarda, Vibrio anguillarum*)

[89]	**Host plant:** *Eupatorium adenophorum*<br><br>**Fungus:** *Phoma multirostrata* EA-12<br><br>**Fermentation:** This fungal strain was cultured on potato dextrose agar medium at 25 °C for 10 days. The agar plugs were inoculated in 500-ml Erlenmeyer flasks, each containing 100 ml of potato dextrose media. Flask cultures were incubated at 28 °C on a rotary shaker at 160 r.p.m. for 2 days as seed culture. Sixty 500-ml Erlenmeyer flasks each containing 150 ml of potato dextrose broth were individually inoculated with 25 ml of seed culture and incubated at 25 °C on a rotary shaker at 160 r.p.m. for 15 days.	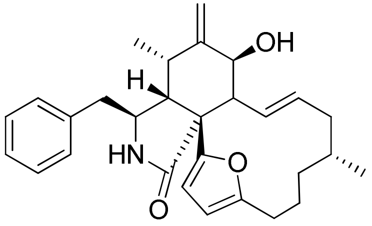<br>multirostratin A (**16**)	It showed cytotoxicity against the HL-60, A-54, SMMC-7721, MCF-7, and SW-480 tumor cell lines
[90]	**Host plant:** *Grateloupia turuturu*, a marine red alga<br><br>**Fungus:** *Paecilomyces variotii* EN-291<br><br>**Fermentation:** The fungal strain was statically fermented in a 1000-mL Erlenmeyer flask containing 300 mL of the PDB medium (potato dextrose broth: 2% mannitol, 1% glucose, 0.3% peptone, 0.5% yeast extract, and seawater added up to 300 mL, pH 6.5–7.0, adjusted with 10% NaOH/flask, 60 flasks) at room temperature for 30 days.	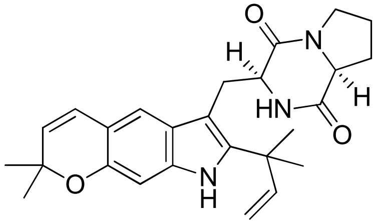 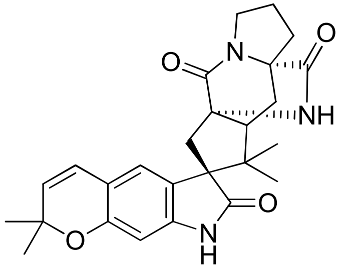<br>dihydrocarneamide A (**60**) *iso*-notoamide B (**61**)	Both compounds displayed weak activity against the NCI-H460 cell line

References

1. Sun, L.T.; Chen, Y.; Yang, H.X.; Li, Z.H.; Liu, J.K.; Wang, G.K.; Feng, T. Bisabolane sesquiterpenes and α-pyrone derivative from endophytic fungus *Zopfiella* sp. *Phytochem. Lett.* **2020**, *37*, 29–32.

2. Lin, X.; Ai, W.; Li, M.; Zhou, X.; Liao, S.; Wang, J.; Liu, J.; Yang, B.; Liu, Y. Collacyclumines A–D from the endophytic fungus *Colletotrichum salsolae* SCSIO 41021 isolated from the mangrove *Kandelia candel*. *Phytochemistry* **2020**, *171*, 112237.

3. Ma, K.L.; Wei, W.J.; Li, H.Y.; Wang, L. Di; Dong, S.H.; Gao, K. Phomotide A, a novel polyketide, from the endophytic fungus *Phomopsis* sp. CFS42. *Tetrahedron Lett.* **2020**, *61*, 151468.

4. Cai, R.; Jiang, H.; Xiao, Z.; Cao, W.; Yan, T.; Liu, Z.; Lin, S.; Long, Y.; She, Z. (-)- And (+)-Asperginulin A, a Pair of Indole Diketopiperazine Alkaloid Dimers with a 6/5/4/5/6 Pentacyclic Skeleton from the Mangrove Endophytic Fungus *Aspergillus* sp. SK-28. *Org. Lett.* **2019**, *21*, 9633–9636.

5. Qiao, Y.; Xu, Q.; Feng, W.; Tao, L.; Li, X.N.; Liu, J.; Zhu, H.; Lu, Y.; Wang, J.; Qi, C.; et al. Asperpyridone A: An Unusual Pyridone Alkaloid Exerts Hypoglycemic Activity through the Insulin Signaling Pathway. *J. Nat. Prod.* **2019**, *82*, 2925–2930.

6. Hu, Z.; Tao, Y.; Tao, X.; Su, Q.; Cai, J.; Qin, C.; Ding, W.; Li, C. Sesquiterpenes with Phytopathogenic Fungi Inhibitory Activities from Fungus *Trichoderma virens* from *Litchi chinensis* Sonn. *J. Agric. Food Chem.* **2019**, *67*, 10646–10652.

7. Wu, Z.; Zhang, X.; Anbari, W.H. Al; Zhou, Q.; Zhou, P.; Zhang, M.; Zeng, F.; Chen, C.; Tong, Q.; Wang, J.; et al. Cysteine Residue Containing Merocytochalasans and 17,18-seco-Aspochalasins from *Aspergillus micronesiensis*. *J. Nat. Prod.* **2019**, *82*, 2653–2658.

8. Wang, W.X.; Lei, X.; Yang, Y.L.; Li, Z.H.; Ai, H.L.; Li, J.; Feng, T.; Liu, J.K. Xylarichalasin A, a Halogenated Hexacyclic Cytochalasan from the Fungus *Xylaria* cf. curta. *Org. Lett.* **2019**, *21*, 6957–6960.

9. Wang, W.-X.; Feng, T.; Li, Z.-H.; Li, J.; Ai, H.-L.; Liu, J.-K. Cytochalasins D1 and C1, unique cytochalasans from endophytic fungus *Xylaria* cf. *curta*. *Tetrahedron Lett.* **2019**, *60*, 150952.

10. Gubiani, J.R.; Oliveira, M.C.S.; Neponuceno, R.A.R.; Camargo, M.J.; Garcez, W.S.; Biz, A.R.; Soares, M.A.; Araujo, A.R.; Bolzani, V. da S.; Lisboa, H.C.F.; et al. Cytotoxic prenylated indole alkaloid produced by the endophytic fungus *Aspergillus terreus* P63. *Phytochem. Lett.* **2019**, *32*, 162–167.

11. Cao, J.; Li, X.-M.; Meng, L.-H.; Konuklugil, B.; Li, X.; Li, H.-L.; Wang, B.-G. Isolation and characterization of three pairs of indolediketopiperazine enantiomers containing infrequent N-methoxy substitution from the marine algal-derived endophytic fungus *Acrostalagmus luteoalbus* TK-43. *Bioorg. Chem.* **2019**, *90*, 103030.

12. Qi, C.; Zhou, Q.; Gao, W.; Liu, M.; Chen, C.; Li, X.N.; Lai, Y.; Zhou, Y.; Li, D.; Hu, Z.; et al. Anti-BACE1 and anti-AchE activities of undescribed spiro-dioxolane-containing meroterpenoids from the endophytic fungus *Aspergillus terreus* Thom. *Phytochemistry* **2019**, *165*, 112041.

13. Feng, L.; Wang, J.; Liu, S.; Zhang, X.J.; Bi, Q.R.; Hu, Y.Y.; Wang, Z.; Tan, N.H. Colletopeptides A-D, Anti-inflammatory Cyclic Tridepsipeptides from the Plant Endophytic Fungus *Colletotrichum* sp. S8. *J. Nat. Prod.* **2019**, *82*, 1434–1441.

14. Li, W.S.; Hu, H.B.; Huang, Z.H.; Yan, R.J.; Tian, L.W.; Wu, J. Phomopsols A and B from the Mangrove Endophytic Fungus *Phomopsis* sp. xy21: Structures, Neuroprotective Effects, and Biogenetic Relationships. *Org. Lett.* 2019, *21*, 7919–7922.

15. Wang, H.H.; Li, G.; Qiao, Y.N.; Sun, Y.; Peng, X.P.; Lou, H.X. Chamiside A, a Cytochalasan with a Tricyclic Core Skeleton from the Endophytic Fungus *Chaetomium nigricolor* F5. *Org. Lett.* **2019**, *21*, 3319–3322.

16. Song, B.; Li, L.Y.; Shang, H.; Liu, Y.; Yu, M.; Ding, G.; Zou, Z.M. Trematosphones A and B, two unique dimeric structures from the desert plant endophytic fungus *trematosphaeria terricola*. *Org. Lett.* **2019**, *21*, 2139–2142.

17. Tang, J.-W.; Xu, H.-C.; Wang, W.-G.; Hu, K.; Zhou, Y.-F.; Chen, R.; Li, X.-N.; Du, X.; Sun, H.-D.; Puno, P.-T. (+)- and (-)-Alternarilactone A: Enantiomers with a Diepoxy-Cage-like Scaffold from an Endophytic *Alternaria* sp. *J. Nat. Prod.* **2019**, *82*, 735–740.

18. Xu, L.-L.; Chen, H.-L.; Hai, P.; Gao, Y.; Xie, C.-D.; Yang, X.-L.; Abe, I. (+)- and (−)-Preuisolactone A: A Pair of Caged Norsesquiterpenoidal Enantiomers with a Tricyclo[4.4.0^{1,6}.0^{2,8}]decane Carbon Skeleton from the Endophytic Fungus *Preussia isomera*. *Org. Lett.* **2019**, *21*, 1078–1081.

19. Liu, H.; Tan, H.; Chen, Y.; Guo, X.; Wang, W.; Guo, H.; Liu, Z.; Zhang, W. Cytorhizins A-D, Four Highly Structure-Combined Benzophenones from the Endophytic Fungus *Cytospora rhizophorae*. *Org. Lett.* **2019**, *21*, 1063–1067.

20. Wang, W.X.; Lei, X.; Ai, H.L.; Bai, X.; Li, J.; He, J.; Li, Z.H.; Zheng, Y.S.; Feng, T.; Liu, J.K. Cytochalasans from the Endophytic Fungus *Xylaria* cf. curta with Resistance Reversal Activity against Fluconazole-Resistant *Candida albicans*. *Org. Lett.* **2019**, *21*, 1108–1111.

21. Wang, W.X.; Li, Z.H.; Feng, T.; Li, J.; Sun, H.; Huang, R.; Yuan, Q.X.; Ai, H.L.; Liu, J.K. Curtachalasins A and B, Two Cytochalasans with a Tetracyclic Skeleton from the Endophytic Fungus *Xylaria curta* E10. *Org. Lett.* **2018**, *20*, 7758–7761.

22. Barakat, F.; Vansteelandt, M.; Triastuti, A.; Jargeat, P.; Jacquemin, D.; Graton, J.; Mejia, K.; Cabanillas, B.; Vendier, L.; Stigliani, J.L.; et al. Thiodiketopiperazines with two spirocyclic centers extracted from *Botryosphaeria mamane*, an endophytic fungus isolated from *Bixa orellana* L. *Phytochemistry* **2019**, *158*, 142–148.

23. Yang, H.X.; Ai, H.L.; Feng, T.; Wang, W.X.; Wu, B.; Zheng, Y.S.; Sun, H.; He, J.; Li, Z.H.; Liu, J.K. Trichothecrotocins A-C, Antiphytopathogenic Agents from Potato Endophytic Fungus *Trichothecium crotocinigenum*. *Org. Lett.* **2018**, *20*, 8069–8072.

24. Pang, X.-J.; Zhang, S.-B.; Chen, H.-L.; Zhao, W.-T.; Yang, D.-F.; Xian, P.-J.; Xu, L.-L.; Tao, Y.-D.; Fu, H.-Y.; Yang, X.-L. Emericelactones A-D: Four novel polyketides produced

by *Emericella* sp. XL 029, a fungus associated the leaves of *Panax notoginseng*. *Tetrahedron Lett.* **2018**, *59*, 4566–4570.

25. Wang, Y.N.; Xia, G.Y.; Wang, L.Y.; Ge, G.B.; Zhang, H.W.; Zhang, J.F.; Wu, Y.Z.; Lin, S. Purpurolide A, 5/5/5 Spirocyclic Sesquiterpene Lactone in Nature from the Endophytic Fungus *Penicillium purpurogenum*. *Org. Lett.* **2018**, *20*, 7341–7344.
26. Guo, Z.-Y.; Tan, M.-H.; Liu, C.-X.; Lv, M.-M.; Deng, Z.-S.; Cao, F.; Zou, K.; Proksch, P. Aspergoterpenins A–D: Four New Antimicrobial Bisabolane Sesquiterpenoid Derivatives from an Endophytic Fungus *Aspergillus versicolor*. *Molecules* **2018**, *23*, 1291.
27. Song, Y.P.; Shi, Z.Z.; Miao, F.P.; Fang, S.T.; Yin, X.L.; Ji, N.Y. Tricholumin A, a Highly Transformed Ergosterol Derivative from the Alga-Endophytic Fungus *Trichoderma asperellum*. *Org. Lett.* **2018**, *20*, 6306–6309.
28. Cai, R.; Wu, Y.; Chen, S.; Cui, H.; Liu, Z.; Li, C.; She, Z. Peniisocoumarins A-J: Isocoumarins from *Penicillium commune* QQF-3, an Endophytic Fungus of the Mangrove Plant *Kandelia candel*. *J. Nat. Prod.* **2018**, *81*, 1376–1383.
29. Yang, M.-H.; Gu, M.-L.; Han, C.; Guo, X.-J.; Yin, G.-P.; Yu, P.; Kong, L.-Y. Aureochaeglobosins A–C, Three [4 + 2] Adducts of Chaetoglobosin and Aureonitol Derivatives from *Chaetomium globosum*. *Org. Lett.* **2018**, *20*, 3345–3348.
30. Xie, S.; Wu, Y.; Qiao, Y.; Guo, Y.; Wang, J.; Hu, Z.; Zhang, Q.; Li, X.; Huang, J.; Zhou, Q.; et al. Protoilludane, Illudalane, and Botryane Sesquiterpenoids from the Endophytic Fungus *Phomopsis* sp. TJ507A. *J. Nat. Prod.* **2018**, *81*, 1311–1320.
31. Li, T.X.; Liu, R.H.; Wang, X.B.; Luo, J.; Luo, J.G.; Kong, L.Y.; Yang, M.H. Hypoxia-Protective Azaphilone Adducts from *Peyronellaea glomerata*. *J. Nat. Prod.* **2018**, *81*, 1148–1153.
32. Kong, Z.; Jing, R.; Wu, Y.; Guo, Y.; Geng, Y.; Ji, J.; Qin, L.; Zheng, C. Trichodermadiones A and B from the solid culture of *Trichoderma atroviride* S361, an endophytic fungus in *Cephalotaxus fortunei*. *Fitoterapia* **2018**, *127*, 362–366.
33. Xiao, J.; Lin, L. Bin; Hu, J.Y.; Duan, D.Z.; Shi, W.; Zhang, Q.; Han, W.B.; Wang, L.; Wang, X.L. Pestalustaines A and B, unprecedented sesquiterpene and coumarin derivatives from endophytic fungus *Pestalotiopsis adusta*. *Tetrahedron Lett.* **2018**, *59*, 1772–1775.
34. Liu, Y.; Kurtán, T.; Mándi, A.; Weber, H.; Wang, C.; Hartmann, R.; Lin, W.; Daletos, G.; Proksch, P. A novel 10-membered macrocyclic lactone from the mangrove-derived endophytic fungus *Annulohypoxylon* sp. *Tetrahedron Lett.* **2018**, *59*, 632–636.
35. Liu, Z.; Qiu, P.; Li, J.; Chen, G.; Chen, Y.; Liu, H.; She, Z. Anti-inflammatory polyketides from the mangrove-derived fungus *Ascomycota* sp. SK2YWS-L. *Tetrahedron* **2018**, *74*, 746–751.
36. Xu, Q.L.; Xiao, Y.S.; Shen, Y.; Wu, H.M.; Zhang, X.; Deng, X.Z.; Wang, T.T.; Li, W.; Tan, R.X.; Jiao, R.H.; et al. Novel chaetospirolactone and orsellide F from an endophytic fungus *Chaetomium* sp. *J. Asian Nat. Prod. Res.* **2018**, *20*, 234–241.
37. Ibrahim, S.R.M.; Mohamed, G.A.; Al Haidari, R.A.; Zayed, M.F.; El-Kholy, A.A.; Elkhayat, E.S.; Ross, S.A. Fusarithioamide B, a new benzamide derivative from the endophytic fungus *Fusarium chlamydosporium* with potent cytotoxic and antimicrobial activities. *Bioorganic Med. Chem.* **2018**, *26*, 786–790.
38. Liu, Z.; Chen, S.; Qiu, P.; Tan, C.; Long, Y.; Lu, Y.; She, Z. (+)- and (-)-Ascomlactone A: A pair of novel dimeric polyketides from a mangrove endophytic fungus: *Ascomycota* sp. SK2YWS-L. *Org. Biomol. Chem.* **2017**, *15*, 10276–10280.
39. Pang, X.; Zhao, J.Y.; Fang, X.M.; Zhang, T.; Zhang, D.W.; Liu, H.Y.; Su, J.; Cen, S.; Yu, L.Y. Metabolites from the Plant Endophytic Fungus *Aspergillus* sp. CPCC 400735 and Their Anti-HIV Activities. *J. Nat. Prod.* **2017**, *80*, 2595–2601.
40. Zhu, X.; Zhou, D.; Liang, F.; Wu, Z.; She, Z.; Li, C. Penochalasin K, a new unusual chaetoglobosin from the mangrove endophytic fungus *Penicillium chrysogenum* V11 and its effective semi-synthesis. *Fitoterapia* **2017**, *123*, 23–28.
41. Cui, H.; Lin, Y.; Luo, M.; Lu, Y.; Huang, X.; She, Z. Diaporisoindoles A−C: Three Isoprenylisoindole Alkaloid Derivatives from the Mangrove Endophytic Fungus *Diaporthe* sp.

SYSU-HQ3. *Org. Lett.* **2017**, *19*, 5621–5624.

42. Wang, F.; Jiang, J.; Hu, S.; Ma, H.; Zhu, H.; Tong, Q.; Cheng, L.; Hao, X.; Zhang, G.; Zhang, Y. Secondary metabolites from endophytic fungus *Chaetomium* sp. induce colon cancer cell apoptotic death. *Fitoterapia* **2017**, *121*, 86–93.

43. Chen, S.; Liu, Z.; Liu, H.; Long, Y.; Chen, D.; Lu, Y.; She, Z. Lasiodiplactone A, a novel lactone from the mangrove endophytic fungus *Lasiodiplodia theobromae* ZJ-HQ1. *Org. Biomol. Chem.* **2017**, *15*, 6338–6341.

44. Gombodorj, S.; Yang, M.H.; Shang, Z.C.; Liu, R.H.; Li, T.X.; Yin, G.P.; Kong, L.Y. New phenalenone derivatives from *Pinellia ternata* tubers derived *Aspergillus* sp. *Fitoterapia* **2017**, *120*, 72–78.

45. Wang, J.-F.; Liang, R.; Liao, S.-R.; Yang, B.; Tu, Z.-C.; Lin, X.-P.; Wang, B.-G.; Liu, Y. Vaccinols J–S, ten new salicyloid derivatives from the marine mangrove-derived endophytic fungus *Pestalotiopsis vaccinii*. *Fitoterapia* **2017**, *120*, 164–170.

46. Chen, L.; Niu, S. Bin; Li, L.; Ding, G.; Yu, M.; Zhang, G.S.; Wang, M.H.; Li, L.Y.; Zhang, T.; Jia, H.M.; et al. Trichoderpyrone, a Unique Polyketide Hybrid with a Cyclopentenone-Pyrone Skeleton from the Plant Endophytic Fungus *Trichoderma gamsii*. *J. Nat. Prod.* **2017**, *80*, 1944–1947.

47. Chen, S.; He, L.; Chen, D.; Cai, R.; Long, Y.; Lu, Y.; She, Z. Talaramide A, an unusual alkaloid from the mangrove endophytic fungus: *Talaromyces* sp. (Hz-YX1) as an inhibitor of mycobacterial PknG. *New J. Chem.* **2017**, *41*, 4273–4276.

48. Li, G.; Kusari, S.; Golz, C.; Laatsch, H.; Strohmann, C.; Spiteller, M. Epigenetic Modulation of Endophytic *Eupenicillium* sp. LG41 by a Histone Deacetylase Inhibitor for Production of Decalin-Containing Compounds. *J. Nat. Prod.* **2017**, *80*, 983–988.

49. Sang, X.N.; Chen, S.F.; Chen, G.; An, X.; Li, S.G.; Lu, X.J.; Zhao, D.; Bai, J.; Wang, H.F.; Pei, Y.H. Two pairs of enantiomeric α-pyrone dimers from the endophytic fungus *Phoma* sp. YN02-P-3. *RSC Adv.* **2017**, *7*, 1943–1946.

50. Hu, Z.; Wu, Y.; Xie, S.; Sun, W.; Guo, Y.; Li, X.N.; Liu, J.; Li, H.; Wang, J.; Luo, Z.; et al. Phomopsterones A and B, two functionalized ergostane-type steroids from the endophytic fungus *Phomopsis* sp. TJ507A. *Org. Lett.* **2017**, *19*, 258–261.

51. Zhu, M.; Zhang, X.; Feng, H.; Dai, J.; Li, J.; Che, Q.; Gu, Q.; Zhu, T.; Li, D. Penicisulfuranols A-F, alkaloids from the mangrove endophytic fungus *Penicillium janthinellum* HDN13-309. *J. Nat. Prod.* **2017**, *80*, 71–75.

52. Feng, Z.-W.; Lv, M.-M.; Li, X.-S.; Zhang, L.; Liu, C.-X.; Guo, Z.-Y.; Deng, Z.-S.; Zou, K.; Proksch, P. Penicitroamide, an Antimicrobial Metabolite with High Carbonylization from the Endophytic Fungus *Penicillium* sp. (NO. 24). *Molecules* **2016**, *21*, 1438.

53. Rangel-Grimaldo, M.; Rivero-Cruz, I.; Madariaga-Mazón, A.; Figueroa, M.; Mata, R. α-Glucosidase Inhibitors from *Preussia minimoides*. *J. Nat. Prod.* **2017**, *80*, 582–587.

54. Huang, S.; Chen, H.; Li, W.; Zhu, X.; Ding, W.; Li, C. Bioactive Chaetoglobosins from the Mangrove Endophytic Fungus *Penicillium chrysogenum*. *Mar. Drugs* **2016**, *14*, 172.

55. Shiono, Y.; Miyazaki, N.; Murayama, T.; Koseki, T.; Harizon; Katja, D.G.; Supratman, U.; Nakata, J.; Kakihara, Y.; Saeki, M.; et al. GSK-3β inhibitory activities of novel dichroloresorcinol derivatives from *Cosmospora vilior* isolated from a mangrove plant. *Phytochem. Lett.* **2016**, *18*, 122–127.

56. Liu, L.; Han, Y.; Xiao, J.; Li, L.; Guo, L.; Jiang, X.; Kong, L.; Che, Y. Chlorotheolides A and B, spiroketals generated via Diels-Alder reactions in the endophytic fungus *Pestalotiopsis theae*. *J. Nat. Prod.* **2016**, *79*, 2616–2623.

57. Meng, L.H.; Wang, C.Y.; Mándi, A.; Li, X.M.; Hu, X.Y.; Kassack, M.U.; Kurtán, T.; Wang, B.G. Three Diketopiperazine Alkaloids with Spirocyclic Skeletons and One Bisthiodiketopiperazine Derivative from the Mangrove-Derived Endophytic Fungus *Penicillium brocae* MA-231. *Org. Lett.* **2016**, *18*, 5304–5307.

58. Fu, G.C.; Yang, Z.D.; Zhou, S.Y.; Li, X.M.; Yu, H.T.; Yao, X.J.; Fang, J.G.; Shu, Z.M.; Xue, H.Y.; Wang, Y.G. Wortmannines A–C, three novel wortmannin derivatives with an unusual five-membered B ring from the endophytic fungus *Talaromyces wortmannii* LGT-4. *Tetrahedron Lett.* **2016**, *57*, 4608–4611.

59. Zhou, H.; Sun, X.; Li, N.; Che, Q.; Zhu, T.; Gu, Q.; Li, D. Isoindolone-Containing Meroperpenoids from the Endophytic Fungus *Emericella nidulans* HDN12-249. *Org. Lett.* **2016**, *18*, 4670–4673.

60. Zhu, M.; Zhang, X.; Feng, H.; Che, Q.; Zhu, T.; Gu, Q.; Li, D. Campyridones A–D, pyridone alkaloids from a mangrove endophytic fungus *Campylocarpon* sp. HDN13-307. *Tetrahedron* **2016**, *72*, 5679–5683.

61. Gao, S.-S.; Li, X.-M.; Williams, K.; Proksch, P.; Ji, N.-Y.; Wang, B.-G. Rhizovarins A−F, Indole-Diterpenes from the Mangrove-Derived Endophytic Fungus *Mucor irregularis* QEN-189. *J. Nat. Prod.* **2016**, *79*, 2066–2074.

62. Elsebai, M.; Ghabbour, H.; Mehiri, M. Unusual Nitrogenous Phenalenone Derivatives from the Marine-Derived Fungus *Coniothyrium cereale*. *Molecules* **2016**, *21*, 178.

63. Li, C.S.; Ren, G.; Yang, B.J.; Miklossy, G.; Turkson, J.; Fei, P.; Ding, Y.; Walker, L.A.; Cao, S. Meroterpenoids with Antiproliferative Activity from a Hawaiian-Plant Associated Fungus *Peyronellaea coffeae-arabicae* FT238. *Org. Lett.* **2016**, *18*, 2335–2338.

64. Ebrahim, W.; El-Neketi, M.; Lewald, L.I.; Orfali, R.S.; Lin, W.; Rehberg, N.; Kalscheuer, R.; Daletos, G.; Proksch, P. Metabolites from the Fungal Endophyte *Aspergillus austroafricanus* in Axenic Culture and in Fungal-Bacterial Mixed Cultures. *J. Nat. Prod.* **2016**, *79*, 914–922.

65. Kajula, M.; Ward, J.M.; Turpeinen, A.; Tejesvi, M. V.; Hokkanen, J.; Tolonen, A.; Häkkänen, H.; Picart, P.; Ihalainen, J.; Sahl, H.G.; et al. Bridged Epipolythiodiketopiperazines from *Penicillium raciborskii*, an Endophytic Fungus of *Rhododendron tomentosum* Harmaja. *J. Nat. Prod.* **2016**, *79*, 685–690.

66. Liu, H.; Li, X.M.; Liu, Y.; Zhang, P.; Wang, J.N.; Wang, B.G. Chermesins A-D: Meroterpenoids with a Drimane-Type Spirosesquiterpene Skeleton from the Marine Algal-Derived Endophytic Fungus *Penicillium chermesinum* EN-480. *J. Nat. Prod.* **2016**, *79*, 806–811.

67. Wang, M.; Sun, Z.H.; Chen, Y.C.; Liu, H.X.; Li, H.H.; Tan, G.H.; Li, S.N.; Guo, X.L.; Zhang, W.M. Cytotoxic cochlioquinone derivatives from the endophytic fungus *Bipolaris sorokiniana* derived from *Pogostemon cablin*. *Fitoterapia* **2016**, *110*, 77–82.

68. Zhang, D.W.; Tao, X.Y.; Liu, J.M.; Chen, R.D.; Zhang, M.; Fang, X.M.; Yu, L.Y.; Dai, J.G. A new polyketide synthase-nonribosomal peptide synthetase hybrid metabolite from plant endophytic fungus *Periconia* sp. *Chinese Chem. Lett.* **2016**, *27*, 640–642.

69. Zhang, M.; Liu, J.-M.; Zhao, J.-L.; Li, N.; Chen, R.-D.; Xie, K.-B.; Zhang, W.-J.; Feng, K.-P.; Yan, Z.; Wang, N.; et al. Two new diterpenoids from the endophytic fungus *Trichoderma* sp. Xy24 isolated from mangrove plant *Xylocarpus granatum*. *Chinese Chem. Lett.* **2016**, *27*, 957–960.

70. Liu, Z.; Chen, Y.; Chen, S.; Liu, Y.; Lu, Y.; Chen, D.; Lin, Y.; Huang, X.; She, Z. Aspterpenacids A and B, Two Sesterterpenoids from a Mangrove Endophytic Fungus *Aspergillus terreus* H010. *Org. Lett.* **2016**, *18*, 1406–1409.

71. Yan, B.C.; Wang, W.G.; Hu, D.B.; Sun, X.; Kong, L.M.; Li, X.N.; Du, X.; Luo, S.H.; Liu, Y.; Li, Y.; et al. Phomopchalasins A and B, Two Cytochalasans with Polycyclic-Fused Skeletons from the Endophytic Fungus *Phomopsis* sp. shj2. *Org. Lett.* **2016**, *18*, 1108–1111.

72. Akone, S.H.; Daletos, G.; Lin, W.; Proksch, P. Unguisin F, a new cyclic peptide from the endophytic fungus *Mucor irregularis*. *Zeitschrift fur Naturforsch. - Sect. C J. Biosci.* **2016**, *71*, 15–19.

73. Zhou, M.; Zhou, K.; He, P.; Wang, K.M.; Zhu, R.Z.; Wang, Y. De; Dong, W.; Li, G.P.; Yang, H.Y.; Ye, Y.Q.; et al. Antiviral and Cytotoxic Isocoumarin Derivatives from an Endophytic Fungus *Aspergillus oryzae*. *Planta Med.* **2016**, *82*, 414–417.

74. Wibowo, M.; Prachyawarakorn, V.; Aree, T.; Mahidol, C.; Ruchirawat, S.; Kittakoop, P. Cytotoxic sesquiterpenes from the endophytic fungus *Pseudolagarobasidium acaciicola*. *Phytochemistry* **2016**, *122*, 126–138.

75. Zhang, D.; Tao, X.; Liu, J.; Chen, R.; Zhang, M.; Li, L.; Fang, X.; Yu, L.Y.; Dai, J. Periconiasin G, a new cytochalasan with unprecedented 7/6/5 tricyclic ring system from the endophytic fungus *Periconia* sp. *Tetrahedron Lett.* **2016**, *57*, 796–799.

76. Yu, G.; Zhou, G.; Zhu, M.; Wang, W.; Zhu, T.; Gu, Q.; Li, D. Neosartoryadins A and B, Fumiquinazoline Alkaloids from a Mangrove-Derived Fungus *Neosartorya udagawae* HDN13-313. *Org. Lett.* **2016**, *18*, 244–247.

77. Liang, X.-R.; Miao, F.-P.; Song, Y.-P.; Guo, Z.-Y.; Ji, N.-Y. Trichocitrin, a new fusicoccane diterpene from the marine brown alga-endophytic fungus *Trichoderma citrinoviride* cf-27. *Nat. Prod. Res.* **2016**, *30*, 1605–1610.

78. Li, T.X.; Yang, M.H.; Wang, X.B.; Wang, Y.; Kong, L.Y. Synergistic Antifungal Meroterpenes and Dioxolanone Derivatives from the Endophytic Fungus *Guignardia* sp. *J. Nat. Prod.* **2015**, *78*, 2511–2520.

79. Nalli, Y.; Mirza, D.N.; Wani, Z.A.; Wadhwa, B.; Mallik, F.A.; Raina, C.; Chaubey, A.; Riyaz-Ul-Hassan, S.; Ali, A. Phialomustin A-D, new antimicrobial and cytotoxic metabolites from an endophytic fungus, *Phialophora mustea*. *RSC Adv.* **2015**, *5*, 95307–95312.

80. Ding, H.E.; Yang, Z.D.; Sheng, L.; Zhou, S.Y.; Li, S.; Yao, X.J.; Zhi, K.K.; Wang, Y.G.; Zhang, F. Secovironolide, a novel furanosteroid scaffold with a five-membered B ring from the endophytic fungus *Talaromyces wortmannii* LGT-4. *Tetrahedron Lett.* **2015**, *56*, 6754–6757.

81. Zhang, P.; Li, X.M.; Liu, H.; Li, X.; Wang, B.G. Two new alkaloids from *Penicillium oxalicum* EN-201, an endophytic fungus derived from the marine mangrove plant *Rhizophora stylosa*. *Phytochem. Lett.* **2015**, *13*, 160–164.

82. Liu, L.; Zhao, C.; Li, L.; Guo, L.; Che, Y. Pestalotriols A and B, new spiro[2.5]octane derivatives from the endophytic fungus *Pestalotiopsis fici*. *RSC Adv.* **2015**, *5*, 78708–78711.

83. Guo, Z.; Ren, F.; Che, Y.; Liu, G.; Liu, L. New Bergamotane Sesquiterpenoids from the Plant Endophytic Fungus *Paraconiothyrium brasiliense*. *Molecules* **2015**, *20*, 14611–14620.

84. Darsih, C.; Prachyawarakorn, V.; Wiyakrutta, S.; Mahidol, C.; Ruchirawat, S.; Kittakoop, P. Cytotoxic metabolites from the endophytic fungus *Penicillium chermesinum*: Discovery of a cysteine-targeted Michael acceptor as a pharmacophore for fragment-based drug discovery, bioconjugation and click reactions. *RSC Adv.* **2015**, *5*, 70595–70603.

85. Li, G.; Kusari, S.; Kusari, P.; Kayser, O.; Spiteller, M. Endophytic *Diaporthe* sp. LG23 Produces a Potent Antibacterial Tetracyclic Triterpenoid. *J. Nat. Prod.* **2015**, *78*, 2128–2132.

86. Li, C.S.; Ding, Y.; Yang, B.J.; Miklossy, G.; Yin, H.Q.; Walker, L.A.; Turkson, J.; Cao, S. A New Metabolite with a Unique 4-Pyranone-γ-Lactam-1,4-Thiazine Moiety from a Hawaiian-Plant Associated Fungus. *Org. Lett.* **2015**, *17*, 3556–3559.

87. Lhamo, S.; Wang, X.B.; Li, T.X.; Wang, Y.; Li, Z.R.; Shi, Y.M.; Yang, M.H.; Kong, L.Y. Three unusual indole diketopiperazine alkaloids from a terrestrial-derived endophytic fungus, *Aspergillus* sp. *Tetrahedron Lett.* **2015**, *56*, 2823–2826.

88. Zhang, P.; Li, X.M.; Li, X.; Wang, B.G. New indole-diterpenoids from the algal-associated fungus *Aspergillus nidulans*. *Phytochem. Lett.* **2015**, *12*, 182–185.

89. Chen, Z.M.; Chen, H.P.; Li, Y.; Feng, T.; Liu, J.K. Cytochalasins from cultures of endophytic fungus *Phoma multirostrata* EA-12. *J. Antibiot. (Tokyo).* **2015**, *68*, 23–26.

90. Zhang, P.; Li, X.-M.; Wang, J.-N.; Li, X.; Wang, B.-G. Prenylated indole alkaloids from the marine-derived fungus *Paecilomyces variotii*. *Chinese Chem. Lett.* **2015**, *26*, 313–316.
